# Developing a fast and catalyst-free protocol to form C=N double bond with high functional group tolerance

**DOI:** 10.1098/rsos.231263

**Published:** 2023-10-04

**Authors:** Bin Zhong, Feng Chen, Yushu Ge, Dan Liu

**Affiliations:** ^1^ Heifei National Laboratory for Physical Sciences at Microscale, the CAS Key Laboratory of Innate Immunity and Chronic Disease, School of Basic Medical Sciences, Division of Life Sciences and Medicine, University of Science and Technology of China, Hefei, 230027, People's Republic of China; ^2^ The First Affiliated Hospital of University of Science and Technology of China, Hefei, Anhui 230001, People's Republic of China

**Keywords:** no catalyst, mild condition, C=N forming, high functional group tolerance, no addition

## Abstract

The carbon–nitrogen double bond (C=N) is a fundamentally important functional group in organic chemistry. This is largely due to the fact that C=N acts as electrophilic synthon to give nitrogen-containing compounds. Here, we report the condensation of primary amine or hydrazine with very electron-deficient aldehyde to form C=N bond in the absence of any catalysts (metals and acids). The protocol performs at room temperature and applies water as co-solvent. Two hundred examples are presented here. With its intrinsic advantages of wide substrate scopes, excellent efficiency (high yields and short reaction time), operational simplicity, mild condition (room temperature as reaction temperature, no catalysts, no additions, water as co-solvent and opening to air) and available starting materials, the protocol can be compatible with various drugs, prodrugs, dyes and pharmacophores containing primary amino group. In addition, we also successfully apply this protocol to rapidly synthesize the core scaffolds of bioactive molecules.

## 1. Introduction

**Figure 1.**
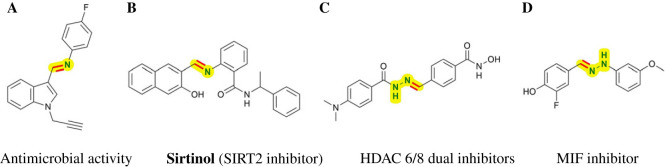
Selected examples of bioactive molecules containing C=N bond.

The carbon–nitrogen double bond is present in many natural products and bioactive molecules with valuable biological activities including anti-tumour, antiviral, antifungal and antibacterial [1–4]. As shown in figure 1, compound **A** possesses significant antimicrobial activity, and shows even more potent than the standard drugs and is more influential against the bacterial than the fungal strains [5]. Sirtinol **B** has been known as a human sirtuin-2 (SIRT2) inhibitor [6], which is reported to induce senescence-like growth arrest in human breast cancer MCF-7 cells and lung cancer H1299 cells [7]. Histone deacetylase 6/8 (HDAC 6/8) dual inhibitor **C** exhibits important anti-tumour activities against hepatocellular carcinoma cells and induces cell cycle arrest in the G2/M phase and eventual cell death in HepG2 cells [8]. Compound **D** has been reported as a migration inhibitory factor (MIF) inhibitor, and a relatively low concentration of **D** can effectively improve survival in sepsis [9]. More importantly, C=N bonds of the imines and hydrazones are versatile electrophiles in organic chemistry that give rise to nitrogen-containing compounds by reduction, cyclization and addition reactions [10–25], which are ubiquitous in natural products, pharmaceuticals, organic materials, dyes and biomolecules [26–28]. Very recently, the C=N unit as linkage has been applied to construct covalent organic frameworks [29].

**Scheme 1.**
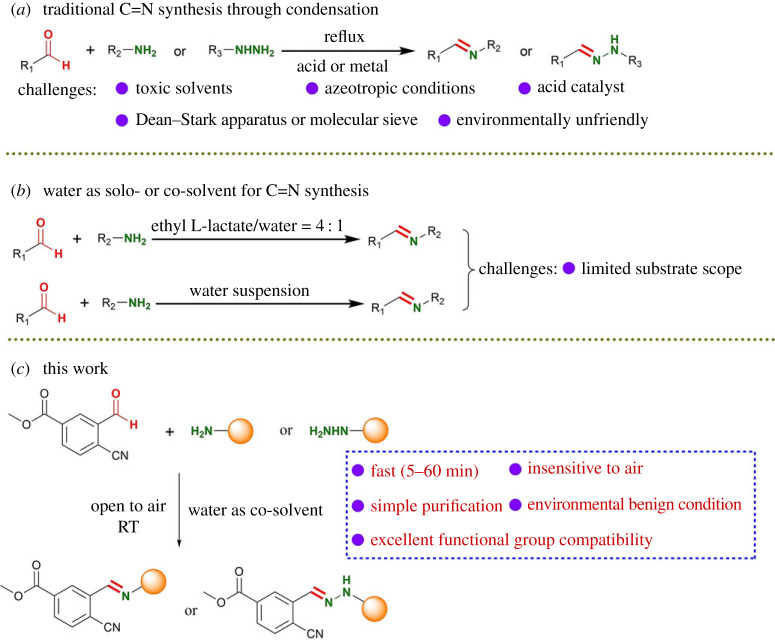
Strategies for the formation of C=N bond and our work.

Traditionally, C=N bond formation is a simple reaction which involves condensation of primary amino group with carbonyl group along with the loss of H_2_O (scheme 1*a*) [30–32]. There are various factors which influence the formation of C=N bond. These factors include concentration, steric effect, electronic effect, pH, temperature and solvents. According to Le Chatelier's principle, adding H_2_O to imine leads to hydrolysis of the C=N to recover the starting materials [33]. Therefore, the direct formation of C=N bond is usually executed in dry solvents (benzene, toluene and CHCl_3_) and needs acid or metal to serve as Lewis acids to catalyse the nucleophilic attack of the amine on the carbonyl group [34]. The equilibrium in this reaction usually favours the reverse reaction, so that drying agents (e.g. molecular sieve) or azeotropic distillation by Dean–Stark apparatus are acquired to remove H_2_O as it is formed in the reaction mixture to drive the reaction towards completion. In view of environmental concerns, researchers have shown remarkable interest in developing sustainable protocols to form C=N bond with microwaves [35–37], ultrasound [38] and IR [39] as energy sources. Furthermore, water and ethyl lactate can be mixed to create polarity conditions that are ideal for the synthesis of aryl aldimines (scheme 1*b*) [40]. More simply, pure water-mediated protocol for the synthesis of C=N bond that requires neither catalyst nor azeotropic removal of water has been reported (scheme 1*b*) [41]. However, these protocols exhibit limited flexibility in the installation of functional groups.

Herein, we demonstrate a novel protocol using water as co-solvent to construct C=N bond at room temperature (RT) without using any catalysts and additions. The protocol shows superiorities including modularity, excellent efficiency (high yields and short reaction time), operational simplicity, mild condition (RT as reaction temperature, no catalysts, no additions, water as co-solvent and insensitive to air) and excellent functional group compatibility (scheme 1*c*). Two hundred compounds containing C=N bond have been synthesized through condensation of primary amine or hydrazine with very electron-deficient aldehyde. The successful synthesize of these compounds including various pharmacophores implies that the protocol has broad application in organic chemistry and significant influence for drug development.

## 2. Results and discussion

Firstly, we tested the feasibility of this protocol. As shown in table 1, 4-cyano-3-formylmethylbenzoate **b** and commercially available aniline were chosen as the model substrates and allowed to react in MeOH with 10 mol% of HCl in open to air at RT (table 1, entry 1). Unfortunately, the desired product methyl (E)-4-cyano-3-((phenylimino)methyl) benzoate **1** was not detected by ^1^H NMR. There was no improvement with increased loading of HCl (entries 2 and 3). Later, we focused on investigation of the reaction with a number of solvents (DMSO, CH_2_Cl_2_, EtOAc, THF, DMF and H_2_O) with 10 mol% of HCl, and the product **1** was also not detected by ^1^H NMR (entries 4–9). Next, the reaction was performed in series of solvents (H_2_O, CH_3_OH, EtOAc, CHCl_3_ and CH_2_Cl_2_) without addition of HCl. Among these, **1** was obtained in 30–75% yields (entries 10–14). We also performed the reaction in 1 ml H_2_O, the product **1** was obtained in only 35% yields (entry 15). These results indicated that acid might have negative effect on the C=N bond formation. In order to optimize the conditions, this reaction was carried out in H_2_O/CH_2_Cl_2_ (3 : 1) or H_2_O/CH_2_Cl_2_ (4 : 1) or H_2_O/CH_3_OH (4 : 1) without addition of HCl under an air atmosphere, the **1** was obtained in 89%, 95% and 42% yields (entries 16–18). To further improve the reaction efficiency, we shortened the reaction time to 5 min to give 95% yield of **1** (entry 19). This protocol using H_2_O as co-solvent was carried out under mild conditions (RT and open to air) in the absence of any catalysts and additions. Meanwhile, the corresponding product **1** could be obtained by simply extracting and removing the CH_2_Cl_2_ under reduced pressure without chromatographic purification or recrystallization.

**Table 1.** Optimization of reaction conditions.^a^

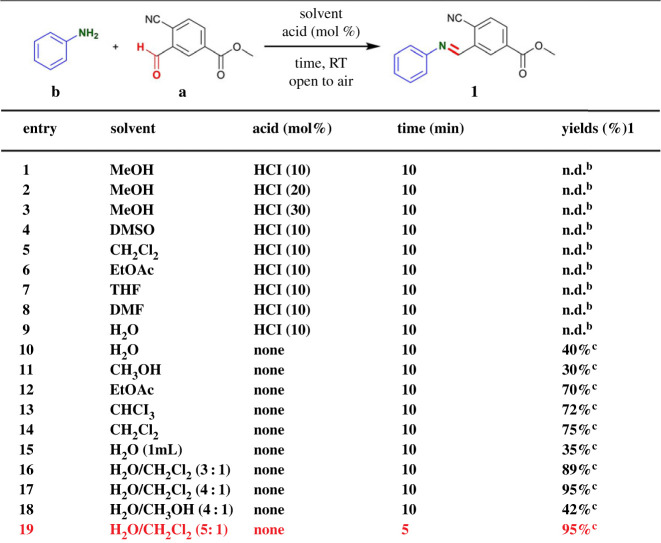

^a^Reaction conditions: reactions were performed with aniline and **b** (0.3 mmol) in 10 ml solvent under an air atmosphere.

^b^n.d. = not detected by ^1^H NMR.

^c^Isolated yield. DMSO = dimethyl sulfoxide. THF = tetrahydrofuran. DMF = *N*, *N*-dimethylformamide.

With the optimized reaction condition in hand, the scopes and generality of the protocol were evaluated (table 2). Firstly, we investigated the substrate scopes of this protocol for monosubstituted anilines (o-, m- and p-substituted anilines) and polysubstituted anilines. As shown in table 2, **b** demonstrated excellent reactivity towards primary amino groups of different reagents. In total, 85–95% yields were obtained in condensation of primary amino groups bearing one or more substituents on the ortho-, meta- or para-position of the anilines with **b** in table 2. To our delight, the present reactions were compatible with electron-donating groups (alkoxy, alkylthiol, alkyl, halogen (Cl, Br, I), *N*, *N*-dimethy, *N*, *N*-diphenyl and unsaturated bonds on aromatic rings), even the strong electron-withdrawing groups (-F, -CN, -CF_3,_ -OCF_3,_ -OCHF_2,_ -OCF_2_Cl, ester carboxyl, ketone carbonyl) on aromatic rings were also tolerated with longer reaction time (table 2). Furthermore, **b** was found to be reactive towards primary amino groups in large steric hindrance environments to obtain corresponding products (**3**, 88%, **70**, 89% and **75**, 90%) with longer reaction time. It is worth mentioning that reactions of **b** with substituted aniline containing electron donor groups or alkane primary amine were completed in 5–15 min. Longer reaction times (30–60 min) were required for some substrates owing to their poor solubility, hindrance or the lower reactivity of their amino groups. Obviously, electron-withdrawing groups on aromatic ring and large steric hindrance around the primary amino group had a little negative effect on reaction time. In the recent past, a significant positive effect of fluorine had been observed through introduction into an aromatic system on drug potency and target selectivity by modulating the physico-chemical parameters and drug metabolism [42]. Cheerfully, fluorine groups (-F, -CF_3,_ -OCF_3,_ -OCHF_2,_ -OCF_2_Cl) on the aromatic ring system were tolerated for this protocol to give the corresponding products in up to 90% yields (table 2). The trimethylsilyl protecting group was also employed, such as 4-((trimethylsilyl)ethynyl) aniline, then the Schiff base **39** was formed in excellent yield (91%). To verify the structure of compounds containing C=N bond, X-ray diffraction (XRD) analysis of a representative product **11** (CCDC 2024116) was performed as shown in table 2.

**Table 2.** Substrate scopes of primary amine.^a,b^

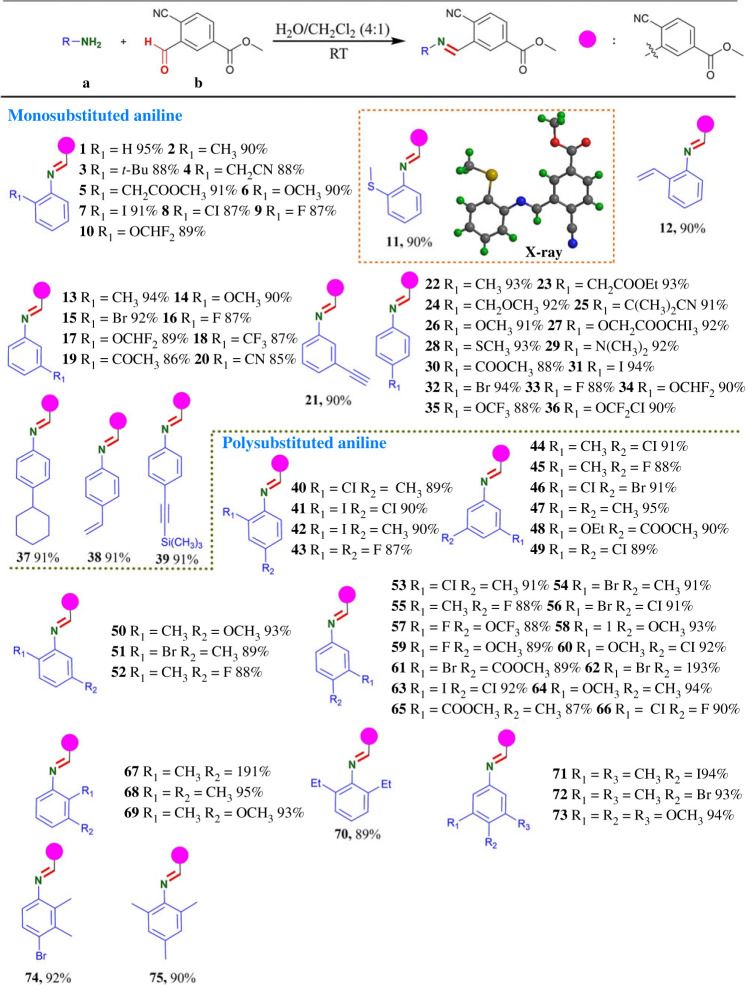

^a^Reaction conditions: reactions were performed with **a** (0.3 mmol) and **b** (0.3 mmol) in 10 ml H_2_O/CH_2_Cl_2_ (4:1) for 5–60 min under an air atmosphere.

^b^Isolated yields.

To further evaluate the chemoselectivity and generality of the protocol, we investigated the reaction of **b** with various pharmacophores and drug fragments containing primary amino group. The benzene ring is not only stable unique dimensionality, π-electron system, aromaticity and rigidity. It is also frequently found in bioactive compounds and materials. Therefore, **b** was examined to benzene derivatives containing primary amino group, and the results are summarized in table 3. Biphenyl scaffolds are privileged substructures used in the discovery and design of therapeutics with high affinity and specificity for a broad range of protein target [43]. The condensation of aminobiphenyls with **b** gave desired products in 89–91% yields (**76**–**78**). Phenoxy aniline derivatives containing primary amino group readily provided the corresponding products **79**–**81** in 90–93% yields. In the case of benzyloxy-substituted aniline, the desired product **82** was obtained in 93% yields. Anilines containing benzyl could also produce the corresponding products **83** in 90% yields. Additionally, **84** containing the tetraphenylmethane motif which had been used in the field of porous materials [44] was obtained in 93% yield. *P*-aminodiphenylamines were able to yield the desired products **85** and **86** in 90% and 92% yields, respectively. Tetraphenylethene (TPE) derivatives are a family of typical aggregation-induced-emission (AIE)-active organic compounds [45]. We synthesized the compound **87** containing the TPE motif in 94% yield. Naphthalene skeletons are important and ubiquitous structural motifs in many important pharmaceutical drugs and have also received much attention in materials science [46,47]. α- or β-amino naphthalene underwent C=N bond formation to generate **88** (94% yield) and **89** (95% yield). Alicyclic anilines were also compatible with this protocol, and gave the desired products **90**–**93** in excellent yields. Fluorene derivatives [48,49], which represented an excellent scaffold for the development of novel pharmaceutical drugs, dyes, polymers and ligands, were tolerated for the protocol to give the corresponding products **94** and **95** in 93% yields. Notably, a single crystal X-ray analysis of the sample of **89** established the absolute configuration of this series of products.

**Table 3.** Substrate scopes of benzene derivative containing primary amino group.^a^^,^^b^

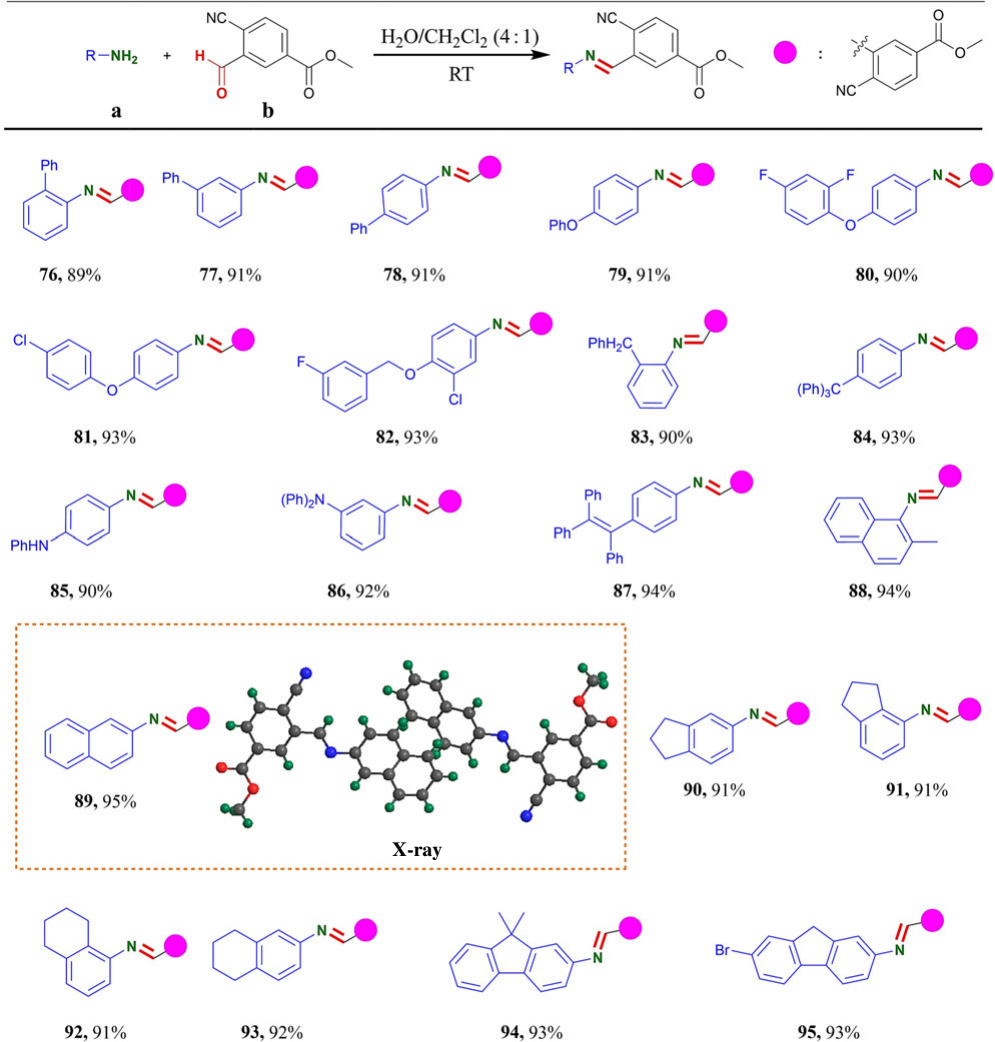

^a^Reaction conditions: reactions were performed with **a** (0.3 mmol) and **b** (0.3 mmol) in 10 ml H_2_O/CH_2_Cl_2_ (4 : 1) for 5–60 min under an air atmosphere.

^b^Isolated yields.

According to the literature, pyridine is the most common N-heterocycle among the small molecule drugs [50]. Piperidine and piperazine are the second and third most commonly used N-heterocycles, respectively, in the FDA approved drugs. Whereas, pyrimidine and pyrazole hold the fourth position followed by indole [50]. Consequently, we further explored the scopes of this protocol with various nitrogen heterocycles containing primary amino group, and the results were summarized in table 4. With the established protocol in hand, indoles and indazoles containing primary amino group could provide the desired products (**96**–**107**) in the excellent yields of up to 95% yields. Aminoquinoline skeletons and *N*, *N-*dimethylbenzimidazolone amine could react with **b** to yield the products **108**–**111** in more than 90% yields. In addition, substituents of piperidine (**112**–**114**), imidazolidin-2-one (**115**), piperazine (**116**), 1-methyl-1H-pyrazole (**117**–**119**) and pyridine (**120**–**126**) on the ortho-, meta- or para-position of anilines could also react with **b** to provide the desired products in up to 93% yields. Indeed, the oxygen-containing heterocycles are fundamental structural units, which are often encountered in a wide range of pharmaceuticals, agrochemicals and natural products [51,52]. The high cation affinity and potency as hydrogen bond acceptors make the O-heterocycles key pharmacophore in drugs [51]. Therefore, various O-heterocycles containing the primary amino group were also investigated and the results were shown in table 5. The condensation of 4-aminobenzo-15-crown-5 with **b** afforded corresponding product **127** in 92% yield. The aminobenzodioxane derivative (**128**), benzofuran amine derivatives (**129**–**133**), benzoxazole amine derivatives (**136**–**139**) and benzo [1,4] oxazinone amine (**140**) were effectively transformed to desired products in up to 94% yields. Additionally, substituents of tetrahydro-2H-pyran (**134**), furan (**135**), oxazole (**141**), isoxazole (**142**) and morphine (**143** and **144**) on the meta-, or para-position of aniline were amenable to this protocol to offer desired products in 89–93% yields. In the past decades, the importance of sulfur-containing heterocycles in new drug discovery programmes has led to increasing attention toward their chemical and biological behaviour [53]. Consequently, S-heterocycles bearing the primary amino group were also investigated, and the results were showed in table 5. The benzothiophene amine (**145**) and benzothiazole amine derivatives (**147**–**150**) were proved to give the corresponding products in 93–95% yields. Meanwhile, the substituents of thiophene (**146**) and thiazole (**151** and **152**) on the meta- or para- position of anilines were smoothly transformed to the corresponding products in excellent yields of up to 92%.

**Table 4.** Substrate scopes of N-heterocycle containing primary amino group.^a^^,^^b^

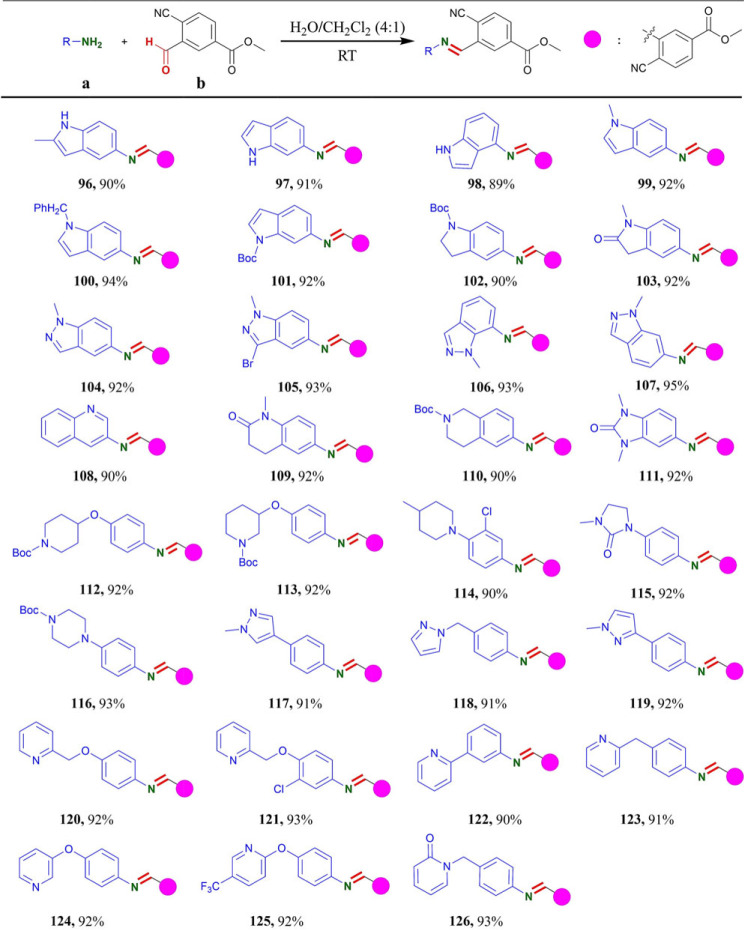

^a^Reaction conditions: reactions were performed with **a** (0.3 mmol) and **b** (0.3 mmol) in 10 ml H_2_O/CH_2_Cl_2_ (4 : 1) for 5–60 min under an air atmosphere.

^b^Isolated yields.

**Table 5.** Substrate scopes of O- or S-heterocycle containing primary amino group.^a^^,^^b^

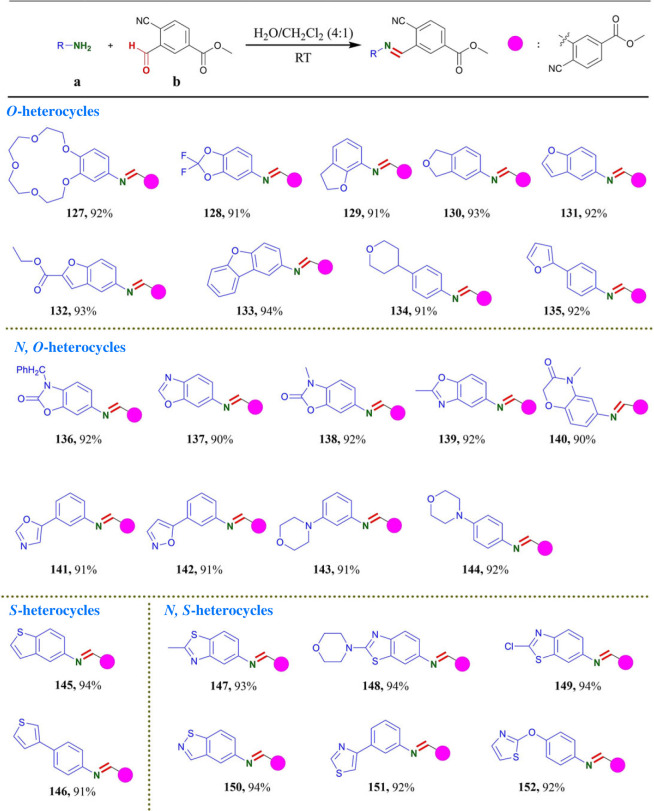

^a^Reaction conditions: reactions were performed with **a** (0.3 mmol) and **b** (0.3 mmol) in 10 ml H_2_O/CH_2_Cl_2_ (4 : 1) for 5–60 min under an air atmosphere.

^b^Isolated yields.

Next, we investigated whether **b** could react toward arylhydrazine in same condition. As shown in table 6, a series of arylhydrazine containing electron-donating groups furnished the desired products within 5–15 min in excellent yields of up to 95% yields. Arylhydrazine containing electron-withdrawing groups were well tolerated in this transformation with longer reaction time (30–60 min) in 88–93% yields (table 6). These results indicated that electronic effect of substituents was negligible in the present reaction since reaction proceeded well with substrates bearing electron donating as well as withdrawing functionalities and the position of the substituents on the phenyl ring had a limited effect on the reaction efficiency. Notably, large steric phenylhydrazine could also provide the desired products in 90% yields (**168** and **169**). 1-naphthylhydrazine hydrochloride and 2-hydrazinopyridine were also compatible with this protocol to obtain the corresponding products (**170** and **171**, 91%). We also applied this protocol for sulfonylhydrazines. The reaction of benzenesulfonohydrazide with **b** gave the desired product in 93% yield (**172**). Arylsulfonyl hydrazines bearing electron-donating group (–OCH_3_) gave corresponding product in 92% yield (**173**). Arylsulfonyl hydrazines including steric hindrance substitute (–CH(CH_3_)_2_) also provided the desired product within 30 min in 89% yields (**174**). It indicated that steric effect had slight influence on this reaction. Meanwhile, aryl acyl hydrazines and aliphatic acyl hydrazines were smoothly transformed to corresponding products in excellent yields of up to 93% (**175**–**178**). Finally, we explored the substrate scopes of *N*, *N*-disubstituted hydrazines and *O*-phenylhydroxylamine hydrochloride for this protocol in table 6. The reaction of **b** with 1-methyl-1-phenylhydrazine delivered the desired products in 91% yield (**179**). To our delight, this protocol was also successfully applied for cyclo-hydrazine derivatives under the same conditions. Some pharmaceutical skeletons, including 1H-indol-amine (**180**) [54], 2-methylindolin-1-amine (**181**) [55], 1-amino-1H-pyrrole-2-carbonitrile (**182**) and 3,4-dihydroquinolin-1(2H)-amine (**183**), were effective reaction partners and could afford the desired products in excellent yields of up to 94% yields. Notably, the condensation of *O*-phenylhydroxylamine with **b** was completed in 15 min to obtain the oxime product **184** with 93% yield.

**Table 6.** Substrate scopes of hydrazine.^a^^,^^b^

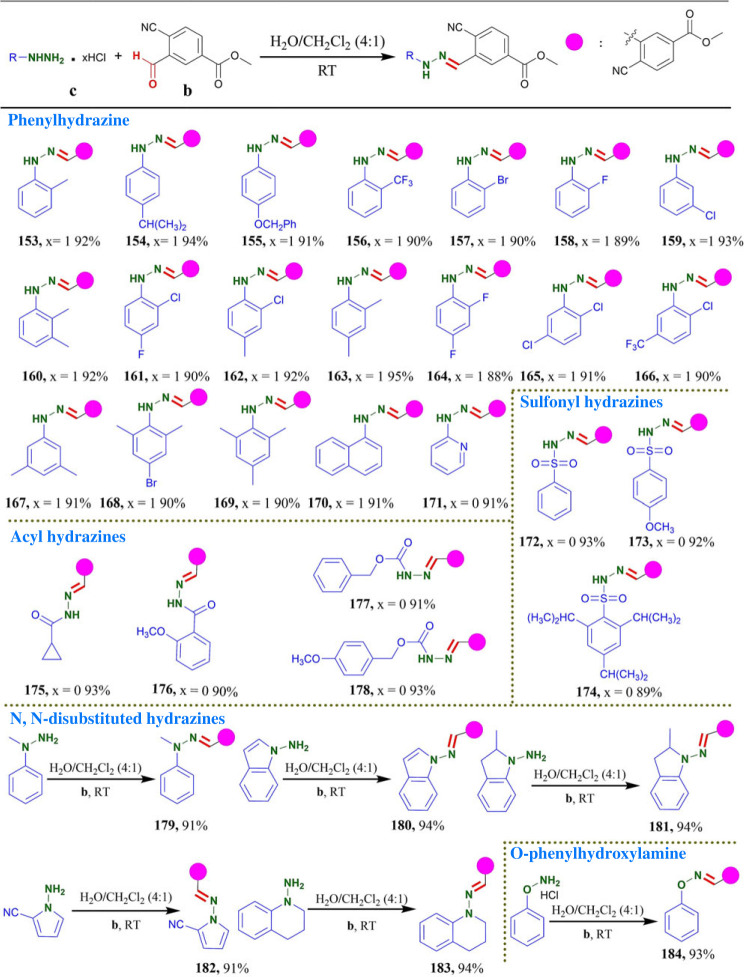

^a^Reaction conditions: reactions were performed with **a** (0.3 mmol) and **b** (0.3 mmol) in 10 ml H_2_O/CH_2_Cl_2_ (4 : 1) for 5–60 min under an air atmosphere.

^b^Isolated yields.

**Figure 2.**
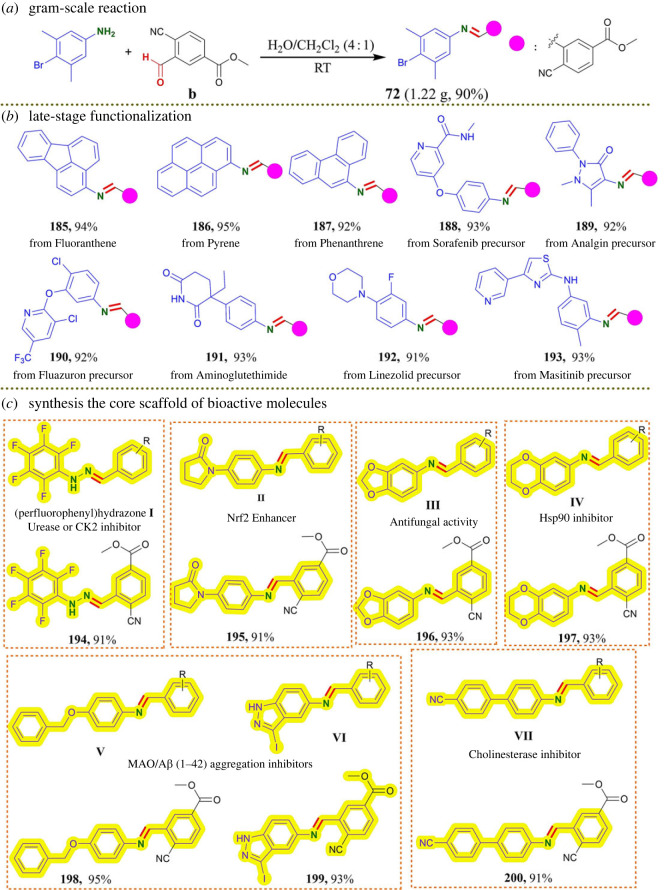
Synthetic application. (*a*) Scale-up synthesis of **72**. (*b*) Late-stage functionalization of dyes, drugs and prodrug. Fluoranthene (**185**), pyrene (**186**), phenanthrene (**187**), sorafenib precursor (**188**), analgin precursor (**189**), fluazuron precursor (**190**), aminoglutethimide (**191**), linezolid precursor (**192**) and masitinib precursor (**193**). (*c*) Synthesis of the core scaffold of bioactive molecules. Synthesis of the core of urease inhibitor or CK2 inhibitor I (**194**), Nrf2 enhancer II (**195**), antifungal activity III (**196**), Hsp90 inhibitor IV (**197**), MAO/Aβ (1–42) aggregation inhibitors V and VI (**198** and **199**) and cholinesterase inhibitor IX (**200**).

Having determined the substrate scopes, we performed a gram-scale reaction **b** with 4-bromo-3,5-dimethylaniline (figure 2*a*) and were pleased to find that the desired product **72** was generated in 90% yield. In addition, to highlight the synthetic potential of this protocol for late-stage functionalization, we investigated several substrates containing primary amino group derived from marketed drugs, prodrugs, dyes and bioactive molecules to showcase the prospective utility of this protocol (figure 2*b*). The fluoranthene [56] (**185**), pyrene [57] (**186**) and phenanthrene [58] (**187**) skeletons, which are often found in natural products and used as fluorescent materials, were tolerated in this protocol to obtain the desired products in up to 95% yields. Sorafenib [59,60] (antirenal and antihepatic carcinomas) precursor (**188**), Analgin [61] (analgesic) precursor (**189**), Fluazuron [62] (pesticide) precursor (**190**), Aminoglutethimide [63] (antineoplastic) (**191**), Linezolid [64,65] (antibiotic) precursor (**192**) and Masitinib [66] (tyrosine kinase inhibitor) precursor (**193**) obtained the corresponding products in 91–93% yields. To further demonstrate the unique advantages of our reaction, we applied our synthetic strategy to the rapid synthesis of the core scaffold of bioactive molecules (figure 2*c*). **I** (urease or protein kinase CK2 inhibitor) [67,68] derivative **194**, **II** (Nrf2 enhancer) [69] derivative **195**, **III** (antifungal activity) [70] derivative **196**, **IV** (Hsp90 inhibitor) [71] derivative **197, V** and **VI** (MAO/A*β* (1–42) aggregation inhibitors) [72,73] derivative **198** and **199**, and VII (cholinesterase inhibitor) [74] derivative **200** were assembled in 91–95% yield. These results further demonstrated the possibility of its utility for pharmaceutical chemistry research.

**Figure 3.**
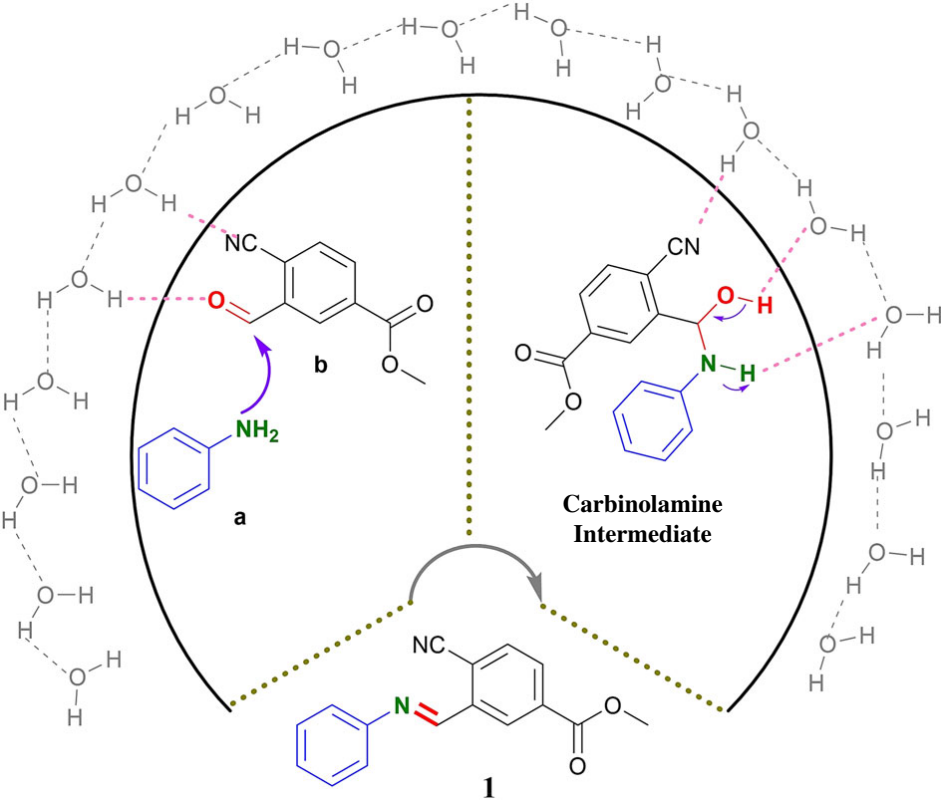
Proposed mechanism.

Based on the literature [75,76], a plausible transition state involving an oil–water interface was assumed in accordance with the Jung and Marcus model [77] to explain the high efficiency of protocol. As shown in figure 3, the nucleophilic attack of the primary amino group to carbonyl at the water–oil phase boundary was facilitated by the hydrogen-bonding interactions between interfacial water and substrates, and the water–oil phase could also stabilize the carbinolamine intermediate through hydrogen-bonding interactions to deliver the product with high efficiency (high yield and fast reaction). Meanwhile, the ortho-cyanide and ester functional groups make the carbonyl more electrophilic.

## 3. Conclusion

In summary, we have developed a novel protocol to form C=N bond without any catalysts and additions at RT. This practical one-step protocol using water as co-solvent represents a simple and economically acceptable route toward the straightforward synthesis of compounds containing C=N bond. With its intrinsic advantages of high yield, fast reaction time (5–60 min), simple purification, no catalysts and additions, mild conditions (H_2_O as co-solvent, RT as reaction temperature and opening to air) and available starting materials, the reaction tolerates a wide range of functional groups and pharmacophores. Different types of primary amine or hydrazine, especially those derived from drugs, prodrug, dye and bioactive molecules are all suitable substrates for this protocol. Moreover, we envision that this protocol potentially can provide a versatile platform for organic synthesis, bio-conjugation, medicinal chemistry, chemical biology and materials science in the future.

## 4. Material and methods

### 4.1. General methods

Unless otherwise stated, all the reagents were purchased from commercial suppliers without further purification. All solvents were distilled from appropriate drying agents prior to use according to Purification of Laboratory Chemicals. The eluents were technical grade. Silica gel (200–300 mesh) for column chromatography and silica GF254 for TLC were produced by Qingdao Marine Chemical Company (China). ^1^H NMR chemical shifts (*δ*) are reported in parts per million relative to tetramethylsilane (0 ppm) or residual CHCl_3_ (7.2600 ppm). ^13^C NMR chemical shifts are reported relative to the centre line signal of the CDCl_3_ triplet at 77.0000 ppm. All ^1^H NMR, ^13^C NMR spectra and ^19^F NMR were recorded in CDCl_3_ on 400 MHz spectrometers. The following abbreviations were used to explain the multiplicities: s, singlet; d, doublet; t, triplet; q, quartet; b, broad; td, triple doublet; dt, double triplet; dq, double quartet; m, multiplet. High-resolution mass spectra (HRMS) were recorded on a matrix assisted laser desorption ionization time of flight mass spectrometer (MALDI-TOF-MS) using electrospray ionization (ESI) as ionization method.

### 4.2. Preparation of the starting material **b**

**S1 to S2.** To a solution of 4-cyano-3-methyl-benzoic acid methyl ester **S1** (6.5 g, 37.10 mmol) in CCl_4_ (80 ml) was added NBS (16.51 g 92.76 mmol) and BPO (1.08 g, 4.45 mmol) successively. The reaction mixture was subsequently stirred for 24 h at 78°C and monitored by TLC (hexane: EtOAc = 10 : 1). After cooling to RT, precipitate of succinimide and unreacted NBS was removed by filtration and washed with CCl_4_ (10 ml). Ultimately, the filtrate was concentrated under reduced pressure to give crude product **S2** (10.1 g), yellow solid. The **S2** was used for the next step without further purification.

**S2 to b.** To a solution of **S2** (10.1 g, 30.33 mmol) in acetonitrile (32 ml) was added a solution of AgNO_3_ (12.88 g, 75.83 mmol) in water (8 ml), and the mixture was heated for 12 h under reflux. After the solution was allowed to cool, AgBr was filtered off and washed with EtOAc (3 × 30 ml), the combined filtrate was washed with water (25 ml) and dried over MgSO_4_. The solvent was evaporated and the residue was purified by column chromatography on silica gel to afford **b** as white solid (4.2 g, two steps yield 60%). ^1^H NMR (400 MHz, CDCl_3_) *δ* 10.36 (s, 1H), 8.65 (d, *J* = 1.7 Hz, 1H), 8.38 (dd, *J* = 8.0, 1.7 Hz, 1H), 7.93 (d, *J* = 8.0 Hz, 1H), 4.00 (s, 3H); ^13^C NMR (101 MHz, CDCl_3_) *δ* 187.67, 164.45, 137.03, 134.66, 134.61, 134.39, 116.92, 115.32, 53.07; HRMS-ESI (*m/z*) [M + H]^+^ calculated for C_10_H_7_NO_3_ 190.04595, found 190.15905.

### 4.3. General procedure for the preparation of compounds containing C=N bond

Aniline derivatives or hydrazine (0.3 mmol) and **b** (0.3 mmol) was added into 10 ml H_2_O/CH_2_Cl_2_ (4 : 1). The reaction mixture was stirred at RT for 5–60 min, Reaction was monitoring by TLC (hexane: EtOAc = 2 : 1). The reaction mixture was extracted three times with CH_2_Cl_2._ The combined organic layers were dried over MgSO_4_ and concentrated under reduced pressure to directly obtain the corresponding products.

**Methyl (E)-4-cyano-3-((phenylimino)methyl)benzoate (1)**

Yellow solid, 75.38 mg, 95% yield; ^1^H NMR (400 MHz, CDCl_3_) *δ* 8.94 (d, *J* = 1.7 Hz, 1H), 8.87 (s, 1H), 8.20 (dd, *J* = 8.1, 1.7 Hz, 1H), 7.83 (d, *J* = 8.0 Hz, 1H), 7.48–7.40 (m, 2H), 7.32 (dt, *J* = 8.1, 1.1 Hz, 3H), 3.99 (s, 3H); ^13^C NMR (101 MHz, CDCl_3_) *δ* 165.11, 154.39, 150.27, 138.63, 134.28, 133.30, 131.46, 129.30, 128.74, 127.42, 121.13, 116.67, 116.21, 52.80; HRMS-ESI (*m/z*) [M + H]^+^ calculated for C_16_H_13_N_2_O_2_ 265.09323, found 265.09753.

**Methyl (E)-4-cyano-3-((o-tolylimino)methyl)benzoate (2)**

Yellow solid, 75.39 mg, 90% yield; ^1^H NMR (400 MHz, CDCl_3_) *δ* 8.89 (d, *J* = 1.7 Hz, 1H), 8.76 (s, 1H), 8.19 (dd, *J* = 8.0, 1.7 Hz, 1H), 7.84 (d, *J* = 8.0 Hz, 1H), 7.29–7.18 (m, 3H), 7.03 (dd, *J* = 7.6, 1.5 Hz, 1H), 4.00 (s, 3H), 2.43 (s, 3H); ^13^C NMR (101 MHz, CDCl_3_) *δ* 165.14, 153.74, 149.36, 138.79, 134.22, 133.54, 132.93, 131.31, 130.54, 129.14, 127.14, 126.79, 117.28, 116.41, 116.32, 52.82, 17.94; HRMS-ESI (*m/z*) [M + H]^+^ calculated for C_17_H_15_N_2_O_2_ 279.10888, found 279.11205.

**Methyl (E)-3-(((2-(tert-butyl)phenyl)imino)methyl)-4-cyanobenzoate (3)**

Yellow solid, 84.35 mg, 88% yield; ^1^H NMR (400 MHz, CDCl_3_) *δ* 8.93 (d, *J* = 1.7 Hz, 1H), 8.74 (s, 1H), 8.21 (dd, *J* = 8.1, 1.7 Hz, 1H), 7.85 (d, *J* = 8.1 Hz, 1H), 7.47–7.43 (m, 1H), 7.28–7.26 (m, 1H), 7.26–7.24 (m, 1H), 6.94–6.91 (m, 1H), 4.00 (s, 3H), 1.47 (s, 9H); ^13^C NMR (101 MHz, CDCl_3_) *δ* 165.16, 152.62, 150.03, 143.66, 138.96, 134.38, 133.41, 131.28, 128.94, 127.15, 127.05, 126.31, 119.06, 116.62, 116.30, 52.88, 35.63, 30.59; HRMS-ESI (*m/z*) [M + H]^+^ calculated for C_20_H_21_N_2_O_2_ 321.15583, found 321.15985.

**Methyl (E)-4-cyano-3-(((2-(cyanomethyl)phenyl)imino)methyl)benzoate (4)**

Light yellow solid, 80.02 mg, 88% yield; ^1^H NMR (400 MHz, CDCl_3_) *δ* 8.84 (s, 1H), 8.82 (d, *J* = 1.7 Hz, 1H), 8.24 (dd, *J* = 8.1, 1.7 Hz, 1H), 7.89 (d, *J* = 8.1 Hz, 1H), 7.54 (dd, *J* = 7.4, 1.5 Hz, 1H), 7.44 (td, *J* = 7.6, 1.6 Hz, 1H), 7.37 (td, *J* = 7.5, 1.4 Hz, 1H), 7.21 (dd, *J* = 7.8, 1.4 Hz, 1H), 4.04 (s, 2H), 4.02 (s, 3H); ^13^C NMR (101 MHz, CDCl_3_) *δ* 164.99, 155.10, 147.83, 138.11, 134.41, 134.08, 131.90, 130.00, 129.52, 129.28, 128.31, 126.22, 118.09, 117.52, 116.58, 116.20, 52.96, 20.31; HRMS-ESI (*m/z*) [M + H]^+^ calculated for C_18_H_14_N_3_O_2_ 304.1041, found 304.1074.

**Methyl (E)-4-cyano-3-(((2-(2-methoxy-2-oxoethyl)phenyl)imino)methyl)benzoate (5)**

Yellow solid, 92.05 mg, 91% yield; ^1^H NMR (400 MHz, CDCl_3_) *δ* 8.86 (d, *J* = 1.7 Hz, 1H), 8.82 (s, 1H), 8.20 (dd, *J* = 8.1, 1.7 Hz, 1H), 7.84 (d, *J* = 8.1 Hz, 1H), 7.37–7.32 (m, 2H), 7.30 (dd, *J* = 7.2, 1.3 Hz, 1H), 7.16 (dd, *J* = 7.7, 1.3 Hz, 1H), 4.00 (s, 3H), 3.91 (s, 2H), 3.67 (s, 3H); ^13^C NMR (101 MHz, CDCl_3_) *δ* 172.24, 165.09, 154.23, 148.82, 138.60, 134.28, 133.57, 131.51, 130.79, 129.95, 129.26, 128.56, 127.66, 117.36, 116.40, 116.35, 52.84, 51.92, 37.52; HRMS-ESI (*m/z*) [M + H]^+^ calculated for C_19_H_17_N_2_O_4_ 337.11436, found 337.11829.

**Methyl (E)-4-cyano-3-(((2-methoxyphenyl)imino)methyl)benzoate (6)**

Red-brown solid, 79.82 mg, 90% yield; ^1^H NMR (400 MHz, CDCl_3_) *δ* 9.00 (s, 1H), 8.97 (d, *J* = 1.7 Hz, 1H), 8.20 (dd, *J* = 8.1, 1.7 Hz, 1H), 7.83 (d, *J* = 8.1 Hz, 1H), 7.27 (td, *J* = 7.8, 1.7 Hz, 1H), 7.15 (dd, *J* = 7.7, 1.7 Hz, 1H), 7.05–6.98 (m, 2H), 3.98 (s, 3H), 3.92 (s, 3H); ^13^C NMR (101 MHz, CDCl_3_) *δ* 165.21, 155.92, 152.38, 139.87, 139.08, 134.32, 133.19, 131.43, 128.68, 128.16, 121.54, 121.11, 116.79, 116.29, 111.77, 55.85, 52.75; HRMS-ESI (*m/z*) [M + H]^+^ calculated for C_17_H_15_N_2_O_3_ 295.10380, found 295.10733.

**Methyl (E)-4-cyano-3-(((2-iodophenyl)imino)methyl)benzoate (7)**

Yellow solid, 106.34 mg, 91% yield; ^1^H NMR (400 MHz, CDCl_3_) *δ* 9.03 (d, *J* = 1.7 Hz, 1H), 8.72 (s, 1H), 8.23 (dd, *J* = 8.1, 1.7 Hz, 1H), 7.93 (dd, *J* = 7.8, 1.3 Hz, 1H), 7.85 (d, *J* = 8.1 Hz, 1H), 7.41 (td, *J* = 7.6, 1.4 Hz, 1H), 7.08 (dd, *J* = 7.9, 1.5 Hz, 1H), 7.00 (td, *J* = 7.6, 1.6 Hz, 1H), 4.00 (s, 3H); ^13^C NMR (101 MHz, CDCl_3_) *δ* 165.03, 155.47, 151.55, 139.28, 138.10, 134.47, 133.30, 131.90, 129.43, 129.10, 128.32, 118.26, 116.84, 116.10, 95.15, 52.89; HRMS-ESI (*m/z*) [M + H]^+^ calculated for C_16_H_12_IN_2_O_2_ 390.98988, found 390.99340.

**Methyl (E)-3-(((2-chlorophenyl)imino)methyl)-4-cyanobenzoate (8)**

Yellow solid, 78.02 mg, 87% yield; ^1^H NMR (400 MHz, CDCl_3_) *δ* 8.97 (d, *J* = 1.7 Hz, 1H), 8.80 (s, 1H), 8.23 (dd, *J* = 8.1, 1.7 Hz, 1H), 7.85 (d, *J* = 8.1 Hz, 1H), 7.47 (dd, *J* = 7.9, 1.5 Hz, 1H), 7.31 (td, *J* = 7.6, 1.5 Hz, 1H), 7.25–7.18 (m, 1H), 7.10 (dd, *J* = 7.8, 1.6 Hz, 1H), 3.99 (s, 3H); ^13^C NMR (101 MHz, CDCl_3_) *δ* 165.02, 156.54, 148.01, 138.17, 134.44, 133.33, 131.98, 130.16, 129.02, 128.35, 127.71, 127.69, 119.73, 116.91, 116.07, 52.85; HRMS-ESI (*m/z*) [M + H]^+^ calculated for C_16_H_12_ClN_2_O_2_ 299.05426, found 299.05819.

**Methyl (E)-4-cyano-3-(((2-fluorophenyl)imino)methyl)benzoate (9)**

Yellow solid, 73.45 mg, 87% yield; ^1^H NMR (400 MHz, CDCl_3_) *δ* 8.96 (s, 2H), 8.23 (dd, *J* = 8.1, 1.7 Hz, 1H), 7.85 (d, *J* = 8.0 Hz, 1H), 7.31–7.15 (m, 4H), 4.00 (s, 3H); ^13^C NMR (101 MHz, CDCl_3_) *δ* 165.07, 157.15 (d, *J* = 3.7 Hz), 138.44, 134.39, 134.36, 133.35, 131.87, 128.86, 128.29 (d, *J* = 7.8 Hz), 124.67 (d, *J* = 3.9 Hz), 122.20 (d, *J* = 1.5 Hz), 116.86, 116.63, 116.43, 116.13, 52.85; ^19^F NMR (376 MHz, CDCl_3_) *δ* -125.33 (ddd, *J* = 12.9, 7.0, 4.2 Hz); HRMS-ESI (*m/z*) [M + H]^+^ calculated for C_16_H_12_FN_2_O_2_ 283.08381, found 283.08813.

**Methyl (E)-4-cyano-3-(((2-(difluoromethoxy)phenyl)imino)methyl)benzoate (10)**

Orange solid, 87.98, 89% yield; ^1^H NMR (400 MHz, CDCl_3_) *δ* 8.89 (d, *J* = 1.7 Hz, 1H), 8.85 (s, 1H), 8.24 (dd, *J* = 8.1, 1.7 Hz, 1H), 7.86 (d, *J* = 8.1 Hz, 1H), 7.33–7.27 (m, 3H), 7.22–7.17 (m, 1H), 6.95–6.53 (m, 1H), 4.00 (s, 3H); ^13^C NMR (101 MHz, CDCl_3_) *δ* 165.02, 156.65, 144.15, 142.72, 138.17, 134.46, 133.58, 131.98, 129.33, 128.24, 126.74, 122.30, 120.26, 116.69, 116.37 (d, *J* = 520.6 Hz), 116.29 (d, *J* = 16.9 Hz), 52.91; ^19^F NMR (376 MHz, CDCl_3_) *δ* -81.28 (d, *J* = 74.4 Hz); HRMS-ESI (*m/z*) [M + H]^+^ calculated for C_17_H_13_F_2_N_2_O_3_ 331.08495, found 331.08810.

**Methyl (E)-4-cyano-3-(((2-(methylthio)phenyl)imino)methyl)benzoate (11)**

Yellow solid, 84.10 mg, 90% yield; ^1^H NMR (400 MHz, CDCl_3_) *δ* 9.00 (d, *J* = 1.7 Hz, 1H), 8.83 (s, 1H), 8.22 (dd, *J* = 8.1, 1.8 Hz, 1H), 7.84 (d, *J* = 8.0 Hz, 1H), 7.30 (ddd, *J* = 8.3, 7.1, 1.4 Hz, 1H), 7.26–7.17 (m, 2H), 7.08 (dd, *J* = 7.8, 1.4 Hz, 1H), 4.00 (s, 3H), 2.49 (s, 3H); ^13^C NMR (101 MHz, CDCl_3_) *δ* 165.15, 154.27, 147.50, 138.59, 135.18, 134.45, 133.30, 131.70, 129.03, 127.87, 125.25, 124.62, 117.43, 116.76, 116.24, 52.85, 14.72; HRMS-ESI (*m/z*) [M + H]^+^ calculated for C_17_H_15_N_2_O_2_S 311.08095, found 311.08507.

**Methyl (E)-4-cyano-3-(((2-vinylphenyl)imino)methyl)benzoate (12)**

Yellow solid, 79.05 mg, 90% yield; ^1^H NMR (400 MHz, CDCl_3_) *δ* 8.90 (d, *J* = 1.7 Hz, 1H), 8.78 (s, 1H), 8.20 (dd, *J* = 8.0, 1.7 Hz, 1H), 7.84 (d, *J* = 8.0 Hz, 1H), 7.63 (dd, *J* = 7.5, 1.8 Hz, 1H), 7.36–7.31 (m, 1H), 7.31–7.28 (m, 1H), 7.26 (d, *J* = 2.4 Hz, 1H), 7.04 (dd, *J* = 7.3, 1.6 Hz, 1H), 5.76 (dd, *J* = 17.7, 1.3 Hz, 1H), 5.34 (dd, *J* = 11.1, 1.3 Hz, 1H), 4.00 (s, 3H); ^13^C NMR (101 MHz, CDCl_3_) *δ* 165.14, 154.50, 148.08, 138.64, 134.34, 133.55, 132.94, 132.20, 131.51, 129.14, 128.79, 127.35, 125.77, 117.98, 116.54, 116.35, 115.32, 52.85; HRMS-ESI (*m/z*) [M + H]^+^ calculated for C_18_H_15_N_2_O_2_ 291.10888, found 291.11299.

**5.3.13** **Methyl (E)-4-cyano-3-((m-tolylimino)methyl)benzoate (13)**

Yellow solid, 78.35 mg, 94% yield; ^1^H NMR (400 MHz, CDCl_3_) *δ* 8.93 (d, *J* = 1.7 Hz, 1H), 8.86 (s, 1H), 8.19 (dd, *J* = 8.0, 1.7 Hz, 1H), 7.83 (d, *J* = 8.0 Hz, 1H), 7.36–7.28 (m, 1H), 7.12 (d, *J* = 6.9 Hz, 3H), 3.99 (s, 3H), 2.41 (s, 3H); ^13^C NMR (101 MHz, CDCl_3_) *δ* 165.14, 154.11, 150.27, 139.22, 138.74, 134.28, 133.30, 131.39, 129.12, 128.73, 128.22, 121.77, 118.21, 116.62, 116.27, 52.80, 21.33; HRMS-ESI (*m/z*) [M + H]^+^ calculated for C_17_H_15_N_2_O_2_ 279.10888, found 279.11348.

**Methyl (E)-4-cyano-3-(((3-methoxyphenyl)imino)methyl)benzoate (14)**

Orange solid, 79.25 mg, 90% yield; ^1^H NMR (400 MHz, CDCl_3_) *δ* 8.95 (d, *J* = 1.7 Hz, 1H), 8.88 (s, 1H), 8.22 (dd, *J* = 8.1, 1.7 Hz, 1H), 7.84 (d, *J* = 8.1 Hz, 1H), 7.37–7.32 (m, 1H), 6.92–6.88 (m, 2H), 6.86 (d, *J* = 1.3 Hz, 1H), 4.00 (s, 3H), 3.87 (s, 3H); ^13^C NMR (101 MHz, CDCl_3_) *δ* 165.17, 160.41, 154.67, 151.75, 138.62, 134.35, 133.37, 131.58, 130.11, 128.83, 116.78, 116.25, 113.30, 112.99, 107.00, 55.41, 52.86; HRMS-ESI (*m/z*) [M + H]^+^ calculated for C_17_H_15_N_2_O_3_ 295.10380, found 295.10693.

**Methyl (E)-3-(((3-bromophenyl)imino)methyl)-4-cyanobenzoate (15)**

Yellow solid, 95.10 mg, 92% yield; ^1^H NMR (400 MHz, CDCl_3_) *δ* 8.90 (d, *J* = 1.7 Hz, 1H), 8.82 (s, 1H), 8.22 (dd, *J* = 8.1, 1.8 Hz, 1H), 7.84 (d, *J* = 8.0 Hz, 1H), 7.47–7.40 (m, 2H), 7.30 (t, *J* = 7.8 Hz, 1H), 7.25–7.20 (m, 1H), 3.99 (s, 3H); ^13^C NMR (101 MHz, CDCl_3_) *δ* 164.99, 155.49, 151.60, 138.13, 134.35, 133.43, 131.85, 130.60, 130.16, 128.94, 124.13, 122.92, 119.89, 116.79, 116.11, 52.86; HRMS-ESI (*m/z*) [M + H]^+^ calculated for C_16_H_12_BrN_2_O_2_ 343.00375, found 343.00728.

**Methyl (E)-4-cyano-3-(((3-fluorophenyl)imino)methyl)benzoate (16)**

Orange solid, 73.78 mg, 87% yield; ^1^H NMR (400 MHz, CDCl_3_) *δ* 8.92 (d, *J* = 1.7 Hz, 1H), 8.84 (s, 1H), 8.22 (dd, *J* = 8.1, 1.7 Hz, 1H), 7.84 (d, *J* = 8.0 Hz, 1H), 7.43–7.34 (m, 1H), 7.12–7.05 (m, 1H), 7.05–6.98 (m, 2H), 3.99 (s, 3H); ^13^C NMR (101 MHz, CDCl_3_) *δ* 165.02, 163.22 (d, *J* = 247.2 Hz), 155.47, 152.03 (d, *J* = 8.8 Hz), 138.20, 134.37, 133.40, 131.82, 130.52 (d, *J* = 9.1 Hz), 128.92, 116.84, 116.75 (d, *J* = 3.0 Hz), 116.10, 114.09 (d, *J* = 21.4 Hz), 108.53 (d, *J* = 22.8 Hz), 52.85; ^19^F NMR (376 MHz, CDCl_3_) *δ* -111.91 (td, *J* = 9.0, 6.2 Hz); HRMS-ESI (*m/z*) [M + H]^+^ calculated for C_16_H_12_FN_2_O_2_ 283.08381, found 283.08728.

**Methyl (E)-4-cyano-3-(((3-(difluoromethoxy)phenyl)imino)methyl)benzoate (17)**

Yellow solid, 88.03 mg, 89% yield; ^1^H NMR (400 MHz, CDCl_3_) *δ* 8.94 (d, *J* = 1.7 Hz, 1H), 8.85 (s, 1H), 8.24 (dd, *J* = 8.1, 1.7 Hz, 1H), 7.86 (d, *J* = 8.1 Hz, 1H), 7.43 (dd, *J* = 8.9, 8.0 Hz, 1H), 7.16 (d, *J* = 0.8 Hz, 1H), 7.10–7.05 (m, 2H), 6.58 (s, 1H), 4.01 (s, 3H); ^13^C NMR (101 MHz, CDCl_3_) *δ* 165.08, 155.63, 151.94, 138.24, 134.44, 133.46, 131.91, 130.54, 129.00, 118.38, 118.10, 117.62, 116.90, 116.15, 115.80, 112.89, 52.91; ^19^F NMR (376 MHz, CDCl_3_) *δ* -80.77, -80.96; HRMS-ESI (*m/z*) [M + H + H_2_O]^+^ calculated for C_17_H_13_F_2_N_2_O_3_ 349.09999, found 349.09976.

**Methyl (E)-4-cyano-3-(((3-(trifluoromethyl)phenyl)imino)methyl)benzoate (18)**

Yellow solid, 86.65 mg, 87% yield; ^1^H NMR (400 MHz, CDCl_3_) *δ* 8.94 (d, *J* = 1.7 Hz, 1H), 8.87 (s, 1H), 8.25 (dd, *J* = 8.1, 1.7 Hz, 1H), 7.87 (d, *J* = 8.1 Hz, 1H), 7.60–7.54 (m, 3H), 7.46 (ddd, *J* = 5.8, 4.5, 2.2 Hz, 1H), 4.01 (s, 3H); ^13^C NMR (101 MHz, CDCl_3_) *δ* 164.98, 156.01, 150.76, 138.03, 134.41, 133.49, 131.99, 129.94, 129.02, 123.81 (dd, *J* = 7.9, 4.2 Hz), 118.46 (q, *J* = 3.7 Hz), 116.83, 116.10, 52.88; ^19^F NMR (376 MHz, CDCl_3_) *δ* -62.62. HRMS-ESI (*m/z*) [M + H]^+^ calculated for C_17_H_12_F_3_N_2_O_2_ 333.08062, found 333.08270.

**Methyl (E)-3-(((3-acetylphenyl)imino)methyl)-4-cyanobenzoate (19)**

Yellow solid, 79.35 mg, 86% yield; ^1^H NMR (400 MHz, CDCl_3_) *δ* 8.92 (d, *J* = 1.7 Hz, 1H), 8.87 (s, 1H), 8.22 (dd, *J* = 8.0, 1.6 Hz, 1H), 7.88 (dd, *J* = 7.5, 1.5 Hz, 1H), 7.86–7.81 (m, 2H), 7.55–7.44 (m, 2H), 3.99 (s, 3H), 2.64 (s, 3H); ^13^C NMR (101 MHz, CDCl_3_) *δ* 197.46, 164.97, 155.55, 150.69, 138.22, 138.18, 134.34, 133.45, 131.80, 129.59, 128.96, 126.98, 125.34, 121.02, 116.72, 116.11, 52.83, 26.71; HRMS-ESI (*m/z*) [M + H]^+^ calculated for C_18_H_15_N_2_O_3_ 307.10380, found 307.10745.

**Methyl (E)-4-cyano-3-(((3-cyanophenyl)imino)methyl)benzoate (20)**

Yellow solid, 74.28 mg, 85% yield; ^1^H NMR (400 MHz, CDCl_3_) *δ* 8.91 (d, *J* = 1.7 Hz, 1H), 8.83 (s, 1H), 8.25 (dd, *J* = 8.1, 1.7 Hz, 1H), 7.87 (d, *J* = 8.1 Hz, 1H), 7.62–7.48 (m, 4H), 4.00 (s, 3H); ^13^C NMR (101 MHz, CDCl_3_) *δ* 164.90, 156.65, 151.01, 137.74, 134.44, 133.54, 132.21, 130.52, 130.31, 129.09, 125.24, 124.81, 118.18, 116.93, 116.01, 113.42, 52.93; HRMS-ESI (*m/z*) [M + H]^+^ calculated for C_17_H_12_N_3_O_2_ 290.08848, found 290.09203.

**Methyl (E)-4-cyano-3-(((3-ethynylphenyl)imino)methyl)benzoate (21)**

Yellow solid, 77.59 mg, 90% yield; ^1^H NMR (400 MHz, CDCl_3_) *δ* 8.94 (d, *J* = 1.7 Hz, 1H), 8.86 (s, 1H), 8.23 (dd, *J* = 8.1, 1.7 Hz, 1H), 7.86 (d, *J* = 8.1 Hz, 1H), 7.46–7.37 (m, 3H), 7.31 (dt, *J* = 7.6, 1.8 Hz, 1H), 4.00 (s, 3H), 3.13 (s, 1H); ^13^C NMR (101 MHz, CDCl_3_) *δ* 165.11, 155.23, 150.35, 138.39, 134.40, 133.43, 131.78, 130.96, 129.40, 128.94, 124.51, 123.28, 121.92, 116.83, 116.20, 82.91, 77.89, 52.90; HRMS-ESI (*m/z*) [M + H]^+^ calculated for C_18_H_13_N_2_O_2_ 289.09323, found 289.09653.

**Methyl (E)-4-cyano-3-((p-tolylimino)methyl)benzoate (22)**

Orange solid, 77.25 mg, 93% yield; ^1^H NMR (400 MHz, CDCl_3_) *δ* 8.94 (d, *J* = 1.7 Hz, 1H), 8.88 (s, 1H), 8.18 (dd, *J* = 8.1, 1.7 Hz, 1H), 7.82 (d, *J* = 8.0 Hz, 1H), 7.29–7.21 (m, 4H), 3.99 (s, 3H), 2.39 (s, 3H); ^13^C NMR (101 MHz, CDCl_3_) *δ* 165.16, 153.26, 147.61, 138.85, 137.66, 134.24, 133.25, 131.23, 129.92, 128.62, 121.20, 116.53, 116.28, 52.77, 21.08; HRMS-ESI (*m/z*) [M + H]^+^ calculated for C_17_H_15_N_2_O_2_ 279.10888, found 279.11200.

**Methyl (E)-4-cyano-3-(((4-(2-ethoxy-2-oxoethyl)phenyl)imino)methyl)benzoate (23)**

Brown solid, 97.25 mg, 93% yield; ^1^H NMR (400 MHz, CDCl_3_) *δ* 8.94 (d, *J* = 1.7 Hz, 1H), 8.88 (s, 1H), 8.20 (dd, *J* = 8.0, 1.7 Hz, 1H), 7.84 (d, *J* = 8.1 Hz, 1H), 7.36 (d, *J* = 8.4 Hz, 2H), 7.29 (d, *J* = 8.3 Hz, 2H), 4.17 (q, *J* = 7.1 Hz, 2H), 4.00 (s, 3H), 3.66 (s, 2H), 1.27 (t, *J* = 7.1 Hz, 3H); ^13^C NMR (101 MHz, CDCl_3_) *δ* 171.40, 165.17, 154.29, 149.21, 138.67, 134.32, 133.55, 133.34, 131.50, 130.26, 128.77, 121.40, 116.70, 116.26, 60.99, 52.85, 40.97, 14.16; HRMS-ESI (*m/z*) [M + H]^+^ calculated for C_20_H_19_N_2_O_4_ 351.13001, found 351.13376.

**Methyl (E)-4-cyano-3-(((4-(methoxymethyl)phenyl)imino)methyl)benzoate (24)**

Red-brown solid, 85.25 mg, 92% yield; ^1^H NMR (400 MHz, CDCl_3_) *δ* 8.96 (d, *J* = 1.7 Hz, 1H), 8.89 (s, 1H), 8.21 (dd, *J* = 8.1, 1.7 Hz, 1H), 7.84 (dd, *J* = 8.1, 0.6 Hz, 1H), 7.42 (d, *J* = 8.4 Hz, 2H), 7.34–7.30 (m, 2H), 4.50 (s, 2H), 4.00 (s, 3H), 3.42 (s, 3H); ^13^C NMR (101 MHz, CDCl_3_) *δ* 165.18, 154.30, 149.72, 138.70, 137.65, 134.35, 133.35, 131.51, 128.80, 128.73, 121.26, 116.71, 116.28, 74.19, 58.16, 52.86; HRMS-ESI (*m/z*) [M + H]^+^ calculated for C_18_H_17_N_2_O_3_ 309.11945, found 309.12369.

**Methyl (E)-4-cyano-3-(((4-(2-cyanopropan-2-yl)phenyl)imino)methyl)benzoate (25)**

Orange solid, 90.25 mg, 91% yield; ^1^H NMR (400 MHz, CDCl_3_) *δ* 8.94 (d, *J* = 1.7 Hz, 1H), 8.86 (s, 1H), 8.22 (dd, *J* = 8.1, 1.7 Hz, 1H), 7.84 (d, *J* = 8.1 Hz, 1H), 7.54 (d, *J* = 8.5 Hz, 2H), 7.33 (d, *J* = 8.6 Hz, 2H), 4.00 (s, 3H), 1.76 (s, 6H); ^13^C NMR (101 MHz, CDCl_3_) *δ* 165.08, 155.01, 149.86, 140.53, 138.41, 134.35, 133.38, 131.69, 128.85, 126.15, 124.30, 121.62, 116.76, 116.18, 52.86, 36.90, 29.13; HRMS-ESI (*m/z*) [M + H]^+^ calculated for C_20_H_18_N_3_O_2_ 332.13543, found 332.13870.

**Methyl (E)-4-cyano-3-(((4-methoxyphenyl)imino)methyl)benzoate (26)**

Brown solid, 80.59 mg, 91% yield; ^1^H NMR (400 MHz, CDCl_3_) *δ* 8.94 (d, *J* = 1.7 Hz, 1H), 8.90 (s, 1H), 8.17 (dd, *J* = 8.1, 1.7 Hz, 1H), 7.82 (d, *J* = 8.1 Hz, 1H), 7.41–7.36 (m, 2H), 6.99–6.95 (m, 2H), 4.00 (s, 3H), 3.86 (s, 3H); ^13^C NMR (101 MHz, CDCl_3_) *δ* 165.29, 159.56, 151.67, 143.00, 139.11, 134.27, 133.29, 131.04, 128.54, 122.94, 116.44, 116.37, 114.55, 55.53, 52.82; HRMS-ESI (*m/z*) [M + H]^+^ calculated for C_17_H_15_N_2_O_3_ 295.10380, found 295.10754.

**Methyl (E)-4-cyano-3-(((4-(2-methoxy-2-oxoethoxy)phenyl)imino)methyl)benzoate (27)**

Red solid, 97.45 mg, 92% yield; ^1^H NMR (400 MHz, CDCl_3_) *δ* 8.92 (d, *J* = 1.7 Hz, 1H), 8.87 (s, 1H), 8.17 (dd, *J* = 8.1, 1.7 Hz, 1H), 7.82 (d, *J* = 8.1 Hz, 1H), 7.35 (d, *J* = 8.9 Hz, 2H), 6.97 (d, *J* = 8.9 Hz, 2H), 4.68 (s, 2H), 3.99 (s, 3H), 3.83 (s, 3H); ^13^C NMR (101 MHz, CDCl_3_) *δ* 169.14, 165.22, 157.55, 152.46, 144.08, 138.92, 134.29, 133.30, 131.19, 128.62, 122.92, 116.47, 116.36, 115.34, 65.49, 52.82, 52.33; HRMS-ESI (*m/z*) [M + H]^+^ calculated for C_19_H_17_N_2_O_5_ 353.10928, found 353.11307.

**Methyl (E)-4-cyano-3-(((4-(methylthio)phenyl)imino)methyl)benzoate (28)**

Red-brown oil, 86.18 mg, 93% yield; ^1^H NMR (400 MHz, CDCl_3_) *δ* 8.92 (d, *J* = 1.7 Hz, 1H), 8.88 (s, 1H), 8.18 (dd, *J* = 8.0, 1.7 Hz, 1H), 7.82 (d, *J* = 8.1 Hz, 1H), 7.30 (s, 4H), 3.99 (s, 3H), 2.52 (s, 3H); ^13^C NMR (101 MHz, CDCl_3_) *δ* 165.13, 153.19, 147.08, 138.73, 138.35, 134.26, 133.30, 131.31, 128.66, 127.13, 121.92, 116.51, 116.27, 52.80, 15.85; HRMS-ESI (*m/z*) [M + H+H_2_O]^+^ calculated for C_17_H_17_N_2_O_3_S 329.09599, found 329.09578.

**Methyl (E)-4-cyano-3-(((4-(dimethylamino)phenyl)imino)methyl)benzoate (29)**

Brown solid, 85.12 mg, 92% yield; ^1^H NMR (400 MHz, CDCl_3_) *δ* 8.96–8.88 (m, 2H), 8.10 (dd, *J* = 8.1, 1.7 Hz, 1H), 7.81–7.74 (m, 1H), 7.41 (d, *J* = 9.1 Hz, 2H), 6.74 (d, *J* = 9.0 Hz, 2H), 3.98 (s, 3H), 3.02 (s, 6H); ^13^C NMR (101 MHz, CDCl_3_) *δ* 165.41, 150.51, 148.12, 139.73, 138.50, 134.07, 133.13, 130.21, 128.14, 123.24, 116.64, 115.76, 112.36, 52.70, 40.42; HRMS-ESI (*m/z*) [M + H]^+^ calculated for C_18_H_18_N_3_O_2_ 308.13543, found 308.13946.

**Methyl (E)-4-cyano-3-(((4-(methoxycarbonyl)phenyl)imino)methyl)benzoate (30)**

Yellow solid, 85.15 mg, 88% yield; ^1^H NMR (400 MHz, CDCl_3_) *δ* 8.94 (d, *J* = 1.7 Hz, 1H), 8.85 (s, 1H), 8.24 (dd, *J* = 8.1, 1.7 Hz, 1H), 8.14–8.09 (m, 2H), 7.86 (dd, *J* = 8.0, 0.6 Hz, 1H), 7.33–7.28 (m, 2H), 4.00 (s, 3H), 3.94 (s, 3H); ^13^C NMR (101 MHz, CDCl_3_) *δ* 166.58, 165.03, 156.09, 154.35, 138.15, 134.45, 133.48, 132.02, 130.99, 129.06, 128.69, 120.89, 116.95, 116.11, 52.92, 52.19; HRMS-ESI (*m/z*) [M + H]^+^ calculated for C_18_H_15_N_2_O_4_ 323.09871, found 323.10175.

**Methyl (E)-4-cyano-3-(((4-iodophenyl)imino)methyl)benzoate (31)**

Orange solid, 109.85 mg, 94% yield; ^1^H NMR (400 MHz, CDCl_3_) *δ* 8.90 (d, *J* = 1.7 Hz, 1H), 8.82 (s, 1H), 8.20 (dd, *J* = 8.1, 1.7 Hz, 1H), 7.83 (dd, *J* = 8.1, 0.6 Hz, 1H), 7.73 (d, *J* = 8.6 Hz, 2H), 7.05 (d, *J* = 8.7 Hz, 2H), 3.98 (s, 3H); ^13^C NMR (101 MHz, CDCl_3_) *δ* 164.99, 154.75, 149.74, 138.37, 138.26, 134.29, 133.37, 131.68, 128.82, 123.09, 116.68, 116.11, 92.35, 52.85; HRMS-ESI (*m/z*) [M + H]^+^ calculated for C_16_H_12_IN_2_O_2_ 390.98988, found 390.99360.

**Methyl (E)-3-(((4-bromophenyl)imino)methyl)-4-cyanobenzoate (32)**

Orange solid, 96.35 mg, 94% yield; ^1^H NMR (400 MHz, CDCl_3_) *δ* 8.90 (d, *J* = 1.7 Hz, 1H), 8.82 (s, 1H), 8.20 (dd, *J* = 8.1, 1.7 Hz, 1H), 7.83 (d, *J* = 8.0 Hz, 1H), 7.53 (d, *J* = 8.6 Hz, 2H), 7.19 (d, *J* = 8.6 Hz, 2H), 3.98 (s, 3H); ^13^C NMR (101 MHz, CDCl_3_) *δ* 165.00, 154.69, 149.06, 138.27, 134.30, 133.37, 132.38, 131.67, 128.81, 122.83, 121.06, 116.67, 116.12, 52.84; HRMS-ESI (*m/z*) [M + H]^+^ calculated for C_16_H_12_BrN_2_O_2_ 343.00375, found 343.00702.

**Methyl (E)-4-cyano-3-(((4-fluorophenyl)imino)methyl)benzoate (33)**

Yellow solid, 74.44 mg, 88% yield; ^1^H NMR (400 MHz, CDCl_3_) *δ* 8.92 (d, *J* = 1.7 Hz, 1H), 8.85 (s, 1H), 8.20 (dd, *J* = 8.1, 1.7 Hz, 1H), 7.84 (d, *J* = 8.0 Hz, 1H), 7.36–7.30 (m, 2H), 7.12 (t, *J* = 8.6 Hz, 2H), 3.99 (s, 3H); ^13^C NMR (101 MHz, CDCl_3_) *δ* 165.08, 162.11 (d, *J* = 247.3 Hz), 153.91 (d, *J* = 1.9 Hz), 146.21 (d, *J* = 3.0 Hz), 138.52, 134.32, 133.35, 131.49, 128.73, 122.88 (d, *J* = 8.5 Hz), 116.61, 116.22, 116.15 (d, *J* = 22.7 Hz), 52.82; ^19^F NMR (376 MHz, CDCl_3_) *δ* -114.53 (tt, *J* = 8.8, 4.9 Hz); HRMS-ESI (*m/z*) [M + H]^+^ calculated for C_16_H_12_FN_2_O_2_ 283.08381, found 283.08774.

**Methyl (E)-4-cyano-3-(((4-(difluoromethoxy)phenyl)imino)methyl)benzoate (34)**

Orange solid, 88.85 mg, 90% yield; ^1^H NMR (400 MHz, CDCl_3_) *δ* 8.93 (d, *J* = 1.7 Hz, 1H), 8.85 (s, 1H), 8.21 (dd, *J* = 8.1, 1.7 Hz, 1H), 7.84 (d, *J* = 8.1 Hz, 1H), 7.34 (d, *J* = 8.9 Hz, 2H), 7.19 (d, *J* = 8.8 Hz, 2H), 6.76–6.33 (m, 1H), 4.00 (s, 3H); ^13^C NMR (101 MHz, CDCl_3_) *δ* 165.11, 154.44, 150.32, 147.46, 138.43, 134.35, 133.39, 131.63, 128.81, 122.65, 120.46, 118.36 (OCHF_2_), 116.70, 116.21 (OCHF_2_), 115.77, 113.18 (305.2 Hz OCHF_2_), 52.88; ^19^F NMR (376 MHz, CDCl_3_) *δ* -80.90 (d, *J* = 73.6 Hz); HRMS-ESI (*m/z*) [M + H]^+^ calculated for C_17_H_13_F_2_N_2_O_3_ 331.08495, found 331.08826.

**Methyl (E)-4-cyano-3-(((4-(trifluoromethoxy)phenyl)imino)methyl)benzoate (35)**

Orange solid, 92.38 mg, 88% yield; ^1^H NMR (400 MHz, CDCl_3_) *δ* 8.92 (d, *J* = 1.7 Hz, 1H), 8.85 (s, 1H), 8.22 (dd, *J* = 8.1, 1.7 Hz, 1H), 7.85 (d, *J* = 8.1 Hz, 1H), 7.37–7.26 (m, 4H), 4.00 (s, 3H); ^13^C NMR (101 MHz, CDCl_3_) *δ* 165.07, 155.16, 148.79, 148.26 (d, *J* = 2.0 Hz), 138.29, 134.40, 133.43, 131.79, 128.91, 122.48, 121.91, 119.16, 116.81, 116.15, 52.88; ^19^F NMR (376 MHz, CDCl_3_) *δ* -57.92. HRMS-ESI (*m/z*) [M + H]^+^ calculated for C_17_H_12_F_3_N_2_O_3_ 349.07553, found 349.07938.

**Methyl (E)-3-(((4-(chlorodifluoromethoxy)phenyl)imino)methyl)-4-cyanobenzoate (36)**

Red solid, 98.56 mg, 90% yield; ^1^H NMR (400 MHz, CDCl_3_) *δ* 8.92 (d, *J* = 1.7 Hz, 1H), 8.85 (s, 1H), 8.22 (dd, *J* = 8.1, 1.7 Hz, 1H), 7.85 (d, *J* = 8.1 Hz, 1H), 7.37–7.28 (m, 4H), 4.00 (s, 3H); ^13^C NMR (101 MHz, CDCl_3_) *δ* 165.07, 155.20, 149.27, 148.94, 138.31, 134.43, 133.44, 131.79, 128.95, 123.96 (d, *J* = 246.7 Hz), 122.43 (d, *J* = 2.8 Hz), 116.81, 116.16, 115.40, 52.87; ^19^F NMR (376 MHz, CDCl_3_) *δ* -25.64; HRMS-ESI (*m/z*) [M + H]^+^ calculated for C_17_H_12_ClF_2_N_2_O_3_ 365.04598, found 365.04928.

**Methyl (E)-4-cyano-3-(((4-cyclohexylphenyl)imino)methyl)benzoate (37)**

Orange solid, 94.95 mg, 91% yield; ^1^H NMR (400 MHz, CDCl_3_) *δ* 8.95 (d, *J* = 1.7 Hz, 1H), 8.89 (s, 1H), 8.19 (dd, *J* = 8.1, 1.7 Hz, 1H), 7.82 (d, *J* = 8.0 Hz, 1H), 7.28 (s, 3H), 3.99 (s, 3H), 2.54 (ddt, *J* = 11.4, 7.1, 3.5 Hz, 1H), 1.94–1.81 (m, 4H), 1.79–1.74 (m, 1H), 1.50–1.35 (m, 4H), 1.28 (tt, *J* = 12.1, 3.7 Hz, 1H). ^13^C NMR (101 MHz, CDCl_3_) *δ* 165.22, 153.35, 147.93, 147.91, 138.92, 134.26, 133.28, 131.25, 128.66, 127.70, 121.26, 116.57, 116.33, 52.82, 44.21, 34.41, 26.81, 26.07; HRMS-ESI (*m/z*) [M + H]^+^ calculated for C_22_H_23_N_2_O_2_ 347.17148, found 347.17497.

**Methyl (E)-4-cyano-3-(((4-vinylphenyl)imino)methyl)benzoate (38)**

Yellow solid, 79.02 mg, 91% yield; ^1^H NMR (400 MHz, CDCl_3_) *δ* 8.94 (d, *J* = 1.7 Hz, 1H), 8.89 (s, 1H), 8.19 (dd, *J* = 8.0, 1.7 Hz, 1H), 7.82 (d, *J* = 8.0 Hz, 1H), 7.48 (d, *J* = 8.4 Hz, 2H), 7.31 (d, *J* = 8.5 Hz, 2H), 6.74 (dd, *J* = 17.6, 10.9 Hz, 1H), 5.78 (dd, *J* = 17.6, 0.9 Hz, 1H), 5.28 (dd, *J* = 10.9, 0.8 Hz, 1H), 3.99 (s, 3H); ^13^C NMR (101 MHz, CDCl_3_) *δ* 165.15, 153.82, 149.57, 138.72, 137.05, 136.05, 134.33, 133.33, 131.43, 128.78, 127.18, 121.56, 116.65, 116.25, 114.24, 52.80; HRMS-ESI (*m/z*) [M + H]^+^ calculated for C_18_H_15_N_2_O_2_ 291.10888, found 291.11249.

**Methyl (E)-4-cyano-3-(((4-((trimethylsilyl)ethynyl)phenyl)imino)methyl)benzoate (39)**

Brown oil, 98.25 mg, 91% yield; ^1^H NMR (400 MHz, CDCl_3_) *δ* 8.92 (d, *J* = 1.7 Hz, 1H), 8.85 (s, 1H), 8.21 (dd, *J* = 8.1, 1.7 Hz, 1H), 7.84 (d, *J* = 8.1 Hz, 1H), 7.53 (d, *J* = 8.5 Hz, 2H), 7.27 (s, 1H), 7.24 (d, *J* = 1.8 Hz, 1H), 3.99 (s, 3H), 0.26 (s, 9H); ^13^C NMR (101 MHz, CDCl_3_) *δ* 165.09, 154.69, 149.98, 138.42, 134.34, 133.41, 133.32, 133.07, 131.69, 128.88, 122.28, 116.71, 116.21, 114.51, 104.59, 95.42, 52.86; HRMS-ESI (*m/z*) [M + H]^+^ calculated for C_21_H_21_N_2_O_2_Si 361.13276, found 361.13615.

**Methyl (E)-3-(((2-chloro-4-methylphenyl)imino)methyl)-4-cyanobenzoate (40)**

Yellow solid, 83.89 mg, 89% yield; ^1^H NMR (400 MHz, CDCl_3_) *δ* 8.98 (d, *J* = 1.7 Hz, 1H), 8.82 (s, 1H), 8.22 (dd, *J* = 8.1, 1.7 Hz, 1H), 7.84 (d, *J* = 8.1 Hz, 1H), 7.32–7.29 (m, 1H), 7.12 (ddd, *J* = 8.0, 1.9, 0.8 Hz, 1H), 7.04 (d, *J* = 8.0 Hz, 1H), 4.00 (s, 3H), 2.37 (s, 3H); ^13^C NMR (101 MHz, CDCl_3_) *δ* 165.09, 155.61, 145.25, 138.43, 138.31, 134.43, 133.29, 131.79, 130.68, 128.96, 128.63, 128.38, 119.33, 116.81, 116.16, 52.85, 20.78; HRMS-ESI (*m/z*) [M + H]^+^ calculated for C_17_H_14_ClN_2_O_2_ 313.06991, found 313.07306.

**Methyl (E)-3-(((4-chloro-2-iodophenyl)imino)methyl)-4-cyanobenzoate (41)**

Yellow solid, 115.32 mg, 90% yield; ^1^H NMR (400 MHz, CDCl_3_) *δ* 9.01 (d, *J* = 1.7 Hz, 1H), 8.70 (s, 1H), 8.24 (dd, *J* = 8.0, 1.7 Hz, 1H), 7.91 (d, *J* = 2.3 Hz, 1H), 7.85 (d, *J* = 8.1 Hz, 1H), 7.39 (dd, *J* = 8.4, 2.3 Hz, 1H), 7.02 (d, *J* = 8.4 Hz, 1H), 4.00 (s, 3H); ^13^C NMR (101 MHz, CDCl_3_) *δ* 164.97, 155.77, 150.13, 138.61, 137.87, 134.54, 133.38, 132.99, 132.10, 129.51, 129.20, 118.64, 116.89, 116.05, 95.68, 52.94; HRMS-ESI (*m/z*) [M + H]^+^ calculated for C_16_H_11_ClIN_2_O_2_ 424.95090, found 424.95423.

**Methyl (E)-4-cyano-3-(((2-iodo-4-methylphenyl)imino)methyl)benzoate (42)**

Yellow solid, 109.42 mg, 90% yield; ^1^H NMR (400 MHz, CDCl_3_) *δ* 9.04 (d, *J* = 1.7 Hz, 1H), 8.73 (s, 1H), 8.22 (dd, *J* = 8.1, 1.7 Hz, 1H), 7.85–7.81 (m, 1H), 7.79–7.74 (m, 1H), 7.22–7.18 (m, 1H), 7.01 (d, *J* = 8.0 Hz, 1H), 4.00 (s, 3H), 2.35 (s, 3H); ^13^C NMR (101 MHz, CDCl_3_) *δ* 165.11, 154.57, 148.85, 139.78, 138.84, 138.36, 134.47, 133.25, 131.70, 130.13, 129.07, 117.75, 116.73, 116.18, 96.02, 52.88, 20.45; HRMS-ESI (*m/z*) [M + H]^+^ calculated for C_17_H_14_IN_2_O_2_ 405.00553, found 405.00903.

**Methyl (E)-4-cyano-3-(((2,4-difluorophenyl)imino)methyl)benzoate (43)**

Yellow solid, 78.26 mg, 87% yield; ^1^H NMR (400 MHz, CDCl_3_) *δ* 8.98–8.89 (m, 2H), 8.22 (dd, *J* = 8.1, 1.7 Hz, 1H), 7.84 (d, *J* = 8.1 Hz, 1H), 7.31–7.22 (m, 1H), 6.95 (tdd, *J* = 7.9, 4.5, 2.6 Hz, 2H), 3.99 (s, 3H); ^13^C NMR (101 MHz, CDCl_3_) *δ* 165.03, 156.74 (dd, *J* = 4.3, 1.9 Hz), 138.35, 134.39, 133.39, 131.89, 128.82, 123.09 (dd, *J* = 9.8, 2.7 Hz), 116.77, 116.12, 111.80 (d, *J* = 3.8 Hz), 111.58 (d, *J* = 3.8 Hz), 105.24, 104.99 (d, *J* = 2.3 Hz), 104.74, 52.87; HRMS-ESI (*m/z*) [M + H]^+^ calculated for C_16_H_11_F_2_N_2_O_2_ 301.07439, found 301.07814.

**Methyl (E)-3-(((3-chloro-5-methylphenyl)imino)methyl)-4-cyanobenzoate (44)**

Yellow solid, 84.97 mg, 91% yield; ^1^H NMR (400 MHz, CDCl_3_) *δ* 8.90 (d, *J* = 1.7 Hz, 1H), 8.82 (s, 1H), 8.22 (dd, *J* = 8.1, 1.7 Hz, 1H), 7.84 (d, *J* = 8.0 Hz, 1H), 7.11 (d, *J* = 0.8 Hz, 2H), 6.99 (d, *J* = 1.3 Hz, 1H), 4.00 (s, 3H), 2.39 (s, 3H); ^13^C NMR (101 MHz, CDCl_3_) *δ* 165.06, 155.20, 151.32, 140.79, 138.29, 134.61, 134.37, 133.43, 131.78, 128.93, 127.93, 120.14, 118.39, 116.76, 116.18, 52.87, 21.19; HRMS-ESI (*m/z*) [M + H]^+^ calculated for C_17_H_14_ClN_2_O_2_ 313.06991, found 313.07364.

**Methyl (E)-4-cyano-3-(((3-fluoro-5-methylphenyl)imino)methyl)benzoate (45)**

Orange solid, 78.02 mg, 88% yield; ^1^H NMR (400 MHz, CDCl_3_) *δ* 8.91 (d, *J* = 1.7 Hz, 1H), 8.82 (s, 1H), 8.21 (dd, *J* = 8.1, 1.7 Hz, 1H), 7.84 (d, *J* = 8.1 Hz, 1H), 6.89 (d, *J* = 1.7 Hz, 1H), 6.83 (dd, *J* = 9.4, 1.6 Hz, 2H), 3.99 (s, 3H), 2.40 (s, 3H); ^13^C NMR (101 MHz, CDCl_3_) *δ* 165.05, 163.13 (d, *J* = 246.3 Hz), 155.15, 151.70 (d, *J* = 9.5 Hz), 141.20 (d, *J* = 9.0 Hz), 138.31, 134.36, 133.39, 131.73, 128.89, 117.45 (d, *J* = 2.5 Hz), 116.77, 116.16, 114.76 (d, *J* = 21.3 Hz), 105.55 (d, *J* = 23.0 Hz), 52.85, 21.36 (d, *J* = 2.0 Hz); ^19^F NMR (376 MHz, CDCl_3_) *δ* -113.13 (t, *J* = 9.5 Hz); HRMS-ESI (*m/z*) [M + H]^+^ calculated for C_17_H_14_FN_2_O_2_ 297.09946, found 297.10349.

**Methyl (E)-3-(((3-bromo-5-chlorophenyl)imino)methyl)-4-cyanobenzoate (46)**

Light yellow solid, 102.98 mg, 91% yield; ^1^H NMR (400 MHz, CDCl_3_) *δ* 8.89 (d, *J* = 1.7 Hz, 1H), 8.80 (s, 1H), 8.25 (dd, *J* = 8.1, 1.7 Hz, 1H), 7.87 (d, *J* = 8.1 Hz, 1H), 7.45 (t, *J* = 1.8 Hz, 1H), 7.33 (t, *J* = 1.8 Hz, 1H), 7.23 (t, *J* = 1.8 Hz, 1H), 4.00 (s, 3H); ^13^C NMR (101 MHz, CDCl_3_) *δ* 164.93, 156.55, 152.31, 137.74, 135.72, 134.46, 133.57, 132.24, 129.77, 129.16, 123.13, 122.55, 120.32, 116.95, 116.03, 52.94; HRMS-ESI (*m/z*) [M + H]^+^ calculated for C_16_H_11_BrClN_2_O_2_ 376.96477, found 376.96906.

**5.3.47** **Methyl (E)-4-cyano-3-(((3,5-dimethylphenyl)imino)methyl)benzoate (47)**

Orange solid, 83.45 mg, 95% yield; ^1^H NMR (400 MHz, CDCl_3_) *δ* 8.92 (d, *J* = 1.7 Hz, 1H), 8.86 (s, 1H), 8.19 (dd, *J* = 8.0, 1.7 Hz, 1H), 7.82 (d, *J* = 8.0 Hz, 1H), 6.95 (d, *J* = 5.1 Hz, 3H), 3.99 (s, 3H), 2.37 (s, 6H); ^13^C NMR (101 MHz, CDCl_3_) *δ* 165.13, 153.80, 150.23, 138.97, 138.80, 134.23, 133.27, 131.29, 129.13, 128.66, 118.87, 116.53, 116.29, 52.76, 21.21; HRMS-ESI (*m/z*) [M + H]^+^ calculated for C_18_H_17_N_2_O_2_ 293.12453, found 293.12857.

**Methyl (E)-4-cyano-3-(((3-ethoxy-5-(methoxycarbonyl)phenyl)imino)methyl) benzoate (48)**

Orange solid, 99.55 mg, 90% yield; ^1^H NMR (400 MHz, CDCl_3_) *δ* 8.90 (d, *J* = 1.7 Hz, 1H), 8.85 (s, 1H), 8.20 (dd, *J* = 8.1, 1.7 Hz, 1H), 7.83 (d, *J* = 8.0 Hz, 1H), 7.54–7.47 (m, 2H), 7.01 (t, *J* = 2.2 Hz, 1H), 4.11 (q, *J* = 7.0 Hz, 2H), 3.98 (s, 3H), 3.92 (s, 3H), 1.44 (t, *J* = 7.0 Hz, 3H); ^13^C NMR (101 MHz, CDCl_3_) *δ* 166.43, 165.01, 159.66, 155.42, 151.61, 138.24, 134.32, 133.44, 132.18, 131.76, 128.97, 116.74, 116.13, 114.06, 113.84, 112.51, 63.99, 52.83, 52.29, 14.66; HRMS-ESI (*m/z*) [M + H]^+^ calculated for C_20_H_19_N_2_O_5_ 367.12493, found 367.12888.

**Methyl (E)-4-cyano-3-(((3,5-dichlorophenyl)imino)methyl)benzoate (49)**

Yellow solid, 89.14 mg, 89% yield; ^1^H NMR (400 MHz, CDCl_3_) *δ* 8.89 (d, *J* = 1.7 Hz, 1H), 8.80 (s, 1H), 8.25 (dd, *J* = 8.1, 1.7 Hz, 1H), 7.87 (d, *J* = 8.0 Hz, 1H), 7.30 (t, *J* = 1.8 Hz, 1H), 7.18 (d, *J* = 1.8 Hz, 2H), 4.00 (s, 3H); ^13^C NMR (101 MHz, CDCl_3_) *δ* 164.94, 156.54, 152.19, 137.76, 135.58, 134.46, 133.56, 132.24, 129.15, 127.01, 119.78, 116.96, 116.04, 52.95; HRMS-ESI (*m/z*) [M + H]^+^ calculated for C_16_H_11_Cl_2_N_2_O_2_ 333.01529, found 333.01878.

**Methyl (E)-4-cyano-3-(((5-methoxy-2-methylphenyl)imino)methyl)benzoate (50)**

Red-brown solid, 85.93 mg, 93% yield; ^1^H NMR (400 MHz, CDCl_3_) *δ* 8.88 (d, *J* = 1.7 Hz, 1H), 8.75 (s, 1H), 8.20 (dd, *J* = 8.1, 1.7 Hz, 1H), 7.87–7.82 (m, 1H), 7.18–7.14 (m, 1H), 6.77 (dd, *J* = 8.3, 2.6 Hz, 1H), 6.58 (d, *J* = 2.6 Hz, 1H), 4.00 (s, 3H), 3.83 (s, 3H), 2.34 (s, 3H); ^13^C NMR (101 MHz, CDCl_3_) *δ* 165.15, 158.50, 154.01, 150.07, 138.70, 134.24, 133.57, 131.40, 131.13, 129.17, 124.84, 116.39, 112.29, 103.34, 55.43, 52.84, 17.04; HRMS-ESI (*m/z*) [M + H]^+^ calculated for C_18_H_17_N_2_O_3_ 309.11945, found 309.12289.

**Methyl (E)-3-(((2-bromo-5-methylphenyl)imino)methyl)-4-cyanobenzoate (51)**

Yellow solid, 95.58 mg, 89% yield; ^1^H NMR (400 MHz, CDCl_3_) *δ* 8.99 (d, *J* = 1.7 Hz, 1H), 8.77 (s, 1H), 8.24 (dd, *J* = 8.1, 1.7 Hz, 1H), 7.85 (d, *J* = 8.1 Hz, 1H), 7.52 (d, *J* = 8.1 Hz, 1H), 6.96 (dd, *J* = 8.1, 2.1 Hz, 1H), 6.90 (d, *J* = 2.1 Hz, 1H), 4.00 (s, 3H), 2.36 (s, 3H); ^13^C NMR (101 MHz, CDCl_3_) *δ* 165.09, 155.97, 149.00, 138.65, 138.29, 134.51, 133.33, 132.88, 131.93, 129.03, 128.83, 120.14, 116.89, 116.16, 115.15, 52.89, 20.95; HRMS-ESI (*m/z*) [M + H]^+^ calculated for C_17_H_14_BrN_2_O_2_ 357.01940, found 357.02337.

**Methyl (E)-4-cyano-3-(((5-fluoro-2-methylphenyl)imino)methyl)benzoate (52)**

Yellow solid, 78.08 mg, 88% yield; ^1^H NMR (400 MHz, CDCl_3_) *δ* 8.88 (d, *J* = 1.7 Hz, 1H), 8.73 (s, 1H), 8.23 (dd, *J* = 8.1, 1.7 Hz, 1H), 7.87 (d, *J* = 8.1 Hz, 1H), 7.21 (dd, *J* = 8.4, 6.1 Hz, 1H), 6.91 (td, *J* = 8.4, 2.6 Hz, 1H), 6.77 (dd, *J* = 9.5, 2.6 Hz, 1H), 4.00 (s, 3H), 2.38 (s, 3H); ^13^C NMR (101 MHz, CDCl_3_) *δ* 165.13, 161.61 (d, *J* = 245.2 Hz), 154.86, 150.28 (d, *J* = 7.3 Hz), 138.42, 134.39, 133.74, 131.74, 131.50 (d, *J* = 8.5 Hz), 129.41, 128.56 (d, *J* = 3.3 Hz), 116.53, 116.38, 113.50 (d, *J* = 21.0 Hz), 104.70 (d, *J* = 22.6 Hz), 52.94, 17.31; ^19^F NMR (376 MHz, CH_3_CN + D_2_O) *δ* -118.76 (q, *J* = 8.2 Hz); HRMS-ESI (*m/z*) [M + H]^+^ calculated for C_17_H_14_FN_2_O_2_ 297.09946, found 297.10321.

**Methyl (E)-3-(((3-chloro-4-methylphenyl)imino)methyl)-4-cyanobenzoate (53)**

Orange solid, 84.98 mg, 91% yield; ^1^H NMR (400 MHz, CDCl_3_) *δ* 8.93 (d, *J* = 1.7 Hz, 1H), 8.86 (s, 1H), 8.22 (dd, *J* = 8.0, 1.7 Hz, 1H), 7.85 (d, *J* = 8.1 Hz, 1H), 7.35 (d, *J* = 2.1 Hz, 1H), 7.31–7.28 (m, 1H), 7.15 (dd, *J* = 8.1, 2.2 Hz, 1H), 4.00 (s, 3H), 2.41 (s, 3H); ^13^C NMR (101 MHz, CDCl_3_) *δ* 165.11, 154.47, 149.02, 138.45, 135.35, 134.97, 134.36, 133.40, 131.65, 131.47, 128.86, 121.89, 119.57, 116.71, 116.22, 52.87, 19.74; HRMS-ESI (*m/z*) [M + H]^+^ calculated for C_17_H_14_ClN_2_O_2_ 313.06991, found 313.07382.

**Methyl (E)-3-(((3-bromo-4-methylphenyl)imino)methyl)-4-cyanobenzoate (54)**

Orange solid, 97.68 mg, 91% yield; ^1^H NMR (400 MHz, CDCl_3_) *δ* 8.91 (d, *J* = 1.8 Hz, 1H), 8.84 (s, 1H), 8.21 (dd, *J* = 8.0, 1.7 Hz, 1H), 7.84 (d, *J* = 8.1 Hz, 1H), 7.53 (d, *J* = 2.1 Hz, 1H), 7.31–7.27 (m, 1H), 7.20 (dd, *J* = 8.1, 2.2 Hz, 1H), 4.00 (s, 3H), 2.43 (s, 3H); ^13^C NMR (101 MHz, CDCl_3_) *δ* 165.10, 154.50, 149.03, 138.42, 137.17, 134.36, 133.41, 131.66, 131.25, 128.87, 125.26, 125.02, 120.22, 116.70, 116.22, 52.87, 22.55; HRMS-ESI (*m/z*) [M + H]^+^ calculated for C_17_H_14_BrN_2_O_2_ 357.01940, found 357.02318.

**Methyl (E)-4-cyano-3-(((4-fluoro-3-methylphenyl)imino)methyl)benzoate (55)**

Orange solid, 77.95 mg, 88% yield; ^1^H NMR (400 MHz, CDCl_3_) *δ* 8.91 (d, *J* = 1.8 Hz, 1H), 8.84 (s, 1H), 8.19 (dd, *J* = 8.0, 1.7 Hz, 1H), 7.83 (d, *J* = 8.0 Hz, 1H), 7.17 (ddd, *J* = 13.7, 5.9, 2.3 Hz, 2H), 7.06 (t, *J* = 8.8 Hz, 1H), 3.99 (s, 3H), 2.33 (d, *J* = 2.1 Hz, 3H); ^13^C NMR (101 MHz, CDCl_3_) *δ* 165.13, 160.74 (d, *J* = 246.0 Hz), 153.52 (d, *J* = 1.9 Hz), 145.85 (d, *J* = 3.2 Hz), 138.66, 134.31, 133.34, 131.40, 128.70, 125.87 (d, *J* = 18.7 Hz), 124.23 (d, *J* = 5.5 Hz), 120.18 (d, *J* = 8.4 Hz), 116.54, 116.29, 115.70 (d, *J* = 23.8 Hz), 52.82, 14.62 (d, *J* = 3.4 Hz); ^19^F NMR (376 MHz, CDCl_3_) *δ* -118.54 (dtt, *J* = 7.0, 4.8, 2.4 Hz); HRMS-ESI (*m/z*) [M + H]^+^ calculated for C_17_H_14_FN_2_O_2_ 297.09946, found 297.10369.

**Methyl (E)-3-(((3-bromo-4-chlorophenyl)imino)methyl)-4-cyanobenzoate (56)**

Yellow solid, 103.15 mg, 91% yield; ^1^H NMR (400 MHz, CDCl_3_) *δ* 8.90 (d, *J* = 1.8 Hz, 1H), 8.82 (s, 1H), 8.24 (dd, *J* = 8.1, 1.7 Hz, 1H), 7.86 (d, *J* = 8.1 Hz, 1H), 7.59 (d, *J* = 2.4 Hz, 1H), 7.51 (d, *J* = 8.5 Hz, 1H), 7.22 (dd, *J* = 8.5, 2.4 Hz, 1H), 4.00 (s, 3H); ^13^C NMR (101 MHz, CDCl_3_) *δ* 164.99, 155.67, 149.55, 137.99, 134.45, 133.52, 133.19, 132.04, 130.83, 129.06, 126.26, 123.05, 121.33, 116.85, 116.10, 52.92; HRMS-ESI (*m/z*) [M + H]^+^ calculated for C_16_H_11_BrClN_2_O_2_ 376.96477, found 376.96868.

**Methyl (E)-4-cyano-3-(((3-fluoro-4-(trifluoromethoxy)phenyl)imino)methyl)benzoate (57)**

Yellow solid, 96.78 mg, 88% yield; ^1^H NMR (400 MHz, CDCl_3_) *δ* 8.92 (d, *J* = 1.7 Hz, 1H), 8.83 (s, 1H), 8.25 (dd, *J* = 8.1, 1.7 Hz, 1H), 7.87 (d, *J* = 8.0 Hz, 1H), 7.38 (td, *J* = 8.3, 1.2 Hz, 1H), 7.18 (dd, *J* = 10.7, 2.4 Hz, 1H), 7.10 (ddd, *J* = 8.7, 2.5, 1.4 Hz, 1H), 4.01 (s, 3H); ^13^C NMR (101 MHz, CDCl_3_) *δ* 164.99, 156.28, 156.08, 153.56, 150.15, 137.85, 134.48, 133.53, 132.17, 129.08, 124.35, 117.15 (d, *J* = 3.6 Hz), 116.99, 116.06, 110.42, 110.22, 52.96; ^19^F NMR (376 MHz, CDCl_3_) *δ* -58.86 (d, *J* = 4.7 Hz); HRMS-ESI (*m/z*) [M + H]^+^ calculated for C_17_H_11_F_4_N_2_O_3_ 367.06611, found 367.06949.

**Methyl (E)-4-cyano-3-(((3-iodo-4-methoxyphenyl)imino)methyl)benzoate (58)**

Orange solid, 117.29 mg, 93% yield; ^1^H NMR (400 MHz, CDCl_3_) *δ* 8.88 (d, *J* = 1.7 Hz, 1H), 8.82 (s, 1H), 8.17 (dd, *J* = 8.1, 1.7 Hz, 1H), 7.86–7.78 (m, 2H), 7.38 (dd, *J* = 8.7, 2.5 Hz, 1H), 6.87 (d, *J* = 8.7 Hz, 1H), 3.99 (s, 3H), 3.92 (s, 3H); ^13^C NMR (101 MHz, CDCl_3_) *δ* 165.10, 157.86, 152.57, 144.01, 138.61, 134.25, 133.35, 132.34, 131.30, 128.69, 123.00, 116.37, 116.30, 110.81, 86.31, 56.63, 52.81; HRMS-ESI (*m/z*) [M + H]^+^ calculated for C_17_H_14_IN_2_O_3_ 421.00044, found 421.00363.

**Methyl (E)-4-cyano-3-(((3-fluoro-4-methoxyphenyl)imino)methyl)benzoate (59)**

Red-brown solid, 83.14 mg, 89% yield; ^1^H NMR (400 MHz, CDCl_3_) *δ* 8.91 (d, *J* = 1.7 Hz, 1H), 8.85 (s, 1H), 8.19 (dd, *J* = 8.1, 1.7 Hz, 1H), 7.83 (d, *J* = 8.1 Hz, 1H), 7.22–7.14 (m, 2H), 7.01 (t, *J* = 8.8 Hz, 1H), 3.99 (s, 3H), 3.94 (s, 3H); ^13^C NMR (101 MHz, CDCl_3_) *δ* 165.17, 153.73, 152.91, 151.27, 147.52 (d, *J* = 10.9 Hz), 143.10 (d, *J* = 7.3 Hz), 138.61, 134.30, 133.37, 131.38, 128.68, 117.84 (d, *J* = 3.3 Hz), 116.40 (d, *J* = 20.2 Hz), 113.41 (d, *J* = 2.6 Hz), 109.57 (d, *J* = 19.2 Hz), 56.42, 52.86; ^19^F NMR (376 MHz, CDCl_3_) *δ* -133.40 (dd, *J* = 11.9, 8.8 Hz); HRMS-ESI (*m/z*) [M + H]^+^ calculated for C_17_H_14_FN_2_O_3_ 313.09438, found 313.09781.

**Methyl (E)-3-(((4-chloro-3-methoxyphenyl)imino)methyl)-4-cyanobenzoate (60)**

Orange solid, 90.35 mg, 92% yield; ^1^H NMR (400 MHz, CDCl_3_) *δ* 8.90 (d, *J* = 1.7 Hz, 1H), 8.84 (s, 1H), 8.20 (dd, *J* = 8.1, 1.7 Hz, 1H), 7.83 (d, *J* = 8.0 Hz, 1H), 7.39 (d, *J* = 8.3 Hz, 1H), 6.91 (d, *J* = 2.3 Hz, 1H), 6.85 (dd, *J* = 8.3, 2.3 Hz, 1H), 3.99 (s, 3H), 3.95 (s, 3H); ^13^C NMR (101 MHz, CDCl_3_) *δ* 165.01, 155.47, 154.66, 149.96, 138.29, 134.33, 133.42, 131.68, 130.57, 128.85, 121.41, 116.66, 116.16, 112.75, 106.29, 56.18, 52.85; HRMS-ESI (*m/z*) [M + H]^+^ calculated for C_17_H_14_ClN_2_O_3_ 329.06482, found 329.06848.

**Methyl (E)-2-bromo-5-((2-cyano-5-(methoxycarbonyl)benzylidene)amino)benzoate (61)**

Yellow solid, 107.15 mg, 89% yield; ^1^H NMR (400 MHz, CDCl_3_) *δ* 8.91 (d, *J* = 1.7 Hz, 1H), 8.85 (s, 1H), 8.23 (dd, *J* = 8.1, 1.7 Hz, 1H), 7.85 (d, *J* = 8.1 Hz, 1H), 7.74–7.69 (m, 2H), 7.29 (dd, *J* = 8.5, 2.7 Hz, 1H), 4.00 (s, 3H), 3.96 (s, 3H); ^13^C NMR (101 MHz, CDCl_3_) *δ* 165.99, 164.97, 155.66, 149.20, 138.03, 135.31, 134.42, 133.53, 132.93, 132.01, 129.06, 125.24, 123.91, 120.06, 116.79, 116.11, 52.91, 52.65; HRMS-ESI (*m/z*) [M + H]^+^ calculated for C_18_H_14_BrN_2_O_4_ 401.00922, found 401.01307.

**Methyl (E)-3-(((3-bromo-4-iodophenyl)imino)methyl)-4-cyanobenzoate (62)**

Yellow solid, 130.43 mg, 93% yield; ^1^H NMR (400 MHz, CDCl_3_) *δ* 8.90 (d, *J* = 1.7 Hz, 1H), 8.82 (s, 1H), 8.23 (dd, *J* = 8.0, 1.7 Hz, 1H), 7.92–7.83 (m, 2H), 7.57 (d, *J* = 2.4 Hz, 1H), 6.97 (dd, *J* = 8.4, 2.4 Hz, 1H), 4.00 (s, 3H); ^13^C NMR (101 MHz, CDCl_3_) *δ* 164.97, 155.75, 151.22, 140.77, 137.98, 134.45, 133.52, 132.06, 130.45, 129.07, 125.31, 121.37, 116.86, 116.08, 99.00, 52.92; HRMS-ESI (*m/z*) [M + H]^+^ calculated for C_16_H_11_BrIN_2_O_2_ 468.90039, found 468.90379.

**Methyl (E)-3-(((4-chloro-3-iodophenyl)imino)methyl)-4-cyanobenzoate (63)**

Yellow solid, 116.95 mg, 92% yield; ^1^H NMR (400 MHz, CDCl_3_) *δ* 8.90 (d, *J* = 1.7 Hz, 1H), 8.81 (s, 1H), 8.24 (dd, *J* = 8.0, 1.7 Hz, 1H), 7.86 (d, *J* = 8.1 Hz, 1H), 7.81 (d, *J* = 2.4 Hz, 1H), 7.50 (d, *J* = 8.4 Hz, 1H), 7.29–7.24 (m, 1H), 4.00 (s, 3H); ^13^C NMR (101 MHz, CDCl_3_) *δ* 164.98, 155.53, 149.32, 138.01, 137.29, 134.43, 133.52, 132.55, 132.00, 129.63, 129.05, 122.27, 116.81, 116.11, 98.45, 52.91; HRMS-ESI (*m/z*) [M + H]^+^ calculated for C_16_H_11_ClIN_2_O_2_ 424.95090, found 424.95557.

**Methyl (E)-4-cyano-3-(((3-methoxy-4-methylphenyl)imino)methyl)benzoate (64)**

Orange solid, 86.98 mg, 94% yield; ^1^H NMR (400 MHz, CDCl_3_) *δ* 8.93 (d, *J* = 1.7 Hz, 1H), 8.89 (s, 1H), 8.18 (dd, *J* = 8.0, 1.7 Hz, 1H), 7.82 (d, *J* = 8.0 Hz, 1H), 7.17 (d, *J* = 8.3 Hz, 1H), 6.90–6.81 (m, 2H), 3.99 (s, 3H), 3.89 (s, 3H), 2.25 (s, 3H); ^13^C NMR (101 MHz, CDCl_3_) *δ* 165.16, 158.23, 153.28, 149.22, 138.82, 134.26, 133.30, 131.26, 130.94, 128.66, 126.37, 116.52, 116.29, 111.91, 104.33, 55.35, 52.78, 16.00; HRMS-ESI (*m/z*) [M + H]^+^ calculated for C_18_H_17_N_2_O_3_ 309.11945, found 309.12266.

**Methyl (E)-5-((2-cyano-5-(methoxycarbonyl)benzylidene)amino)-2-methylbenzoate (65)**

Yellow solid, 88.06 mg, 87% yield; ^1^H NMR (400 MHz, CDCl_3_) *δ* 8.92 (d, *J* = 1.7 Hz, 1H), 8.88 (s, 1H), 8.20 (dd, *J* = 8.1, 1.7 Hz, 1H), 7.87 (d, *J* = 2.3 Hz, 1H), 7.83 (d, *J* = 8.1 Hz, 1H), 7.38 (dd, *J* = 8.1, 2.4 Hz, 1H), 7.31 (d, *J* = 8.2 Hz, 1H), 3.99 (s, 3H), 3.92 (s, 3H), 2.62 (s, 3H); ^13^C NMR (101 MHz, CDCl_3_) *δ* 167.39, 165.07, 154.33, 147.82, 139.59, 138.51, 134.32, 133.39, 132.70, 131.56, 130.38, 128.85, 124.65, 123.31, 116.61, 116.22, 52.82, 51.97, 21.32; HRMS-ESI (*m/z*) [M + H]^+^ calculated for C_19_H_17_N_2_O_4_ 337.11436, found 337.11853.

**Methyl (E)-3-(((3-chloro-4-fluorophenyl)imino)methyl)-4-cyanobenzoate (66)**

Light yellow solid, 83.06 mg, 90% yield; ^1^H NMR (400 MHz, CDCl_3_) *δ* 8.90 (d, *J* = 1.7 Hz, 1H), 8.83 (s, 1H), 8.23 (dd, *J* = 8.1, 1.7 Hz, 1H), 7.85 (d, *J* = 8.0 Hz, 1H), 7.40 (ddd, *J* = 6.6, 2.1, 0.8 Hz, 1H), 7.23–7.19 (m, 2H), 4.00 (s, 3H); ^13^C NMR (101 MHz, CDCl_3_) *δ* 165.03, 157.35 (d, *J* = 249.9 Hz), 155.04 (d, *J* = 2.1 Hz), 146.77, 138.11, 134.41, 133.49, 131.89, 128.94, 123.36, 121.77 (d, *J* = 19.0 Hz), 121.12 (d, *J* = 7.3 Hz), 117.14 (d, *J* = 22.3 Hz), 116.76, 116.16, 52.92; ^19^F NMR (376 MHz, CDCl_3_) *δ* -116.94 (q, *J* = 6.4 Hz); HRMS-ESI (*m/z*) [M + H]^+^ calculated for C_16_H_11_ClFN_2_O_2_ 317.04484, found 317.04792.

**Methyl (E)-4-cyano-3-(((3-iodo-2-methylphenyl)imino)methyl)benzoate (67)**

Yellow solid, 110.75 mg, 91%;^1^H NMR (400 MHz, CDCl_3_) *δ* 8.87 (d, *J* = 1.7 Hz, 1H), 8.69 (s, 1H), 8.21 (dd, *J* = 8.0, 1.7 Hz, 1H), 7.85 (d, *J* = 8.1 Hz, 1H), 7.75 (dd, *J* = 7.1, 2.0 Hz, 1H), 7.00–6.89 (m, 2H), 4.00 (s, 3H), 2.53 (s, 3H);^13^C NMR (101 MHz, CDCl_3_) *δ* 165.03, 154.90, 150.00, 138.28, 137.64, 135.81, 134.31, 133.60, 131.68, 129.24, 128.20, 117.64, 116.51, 116.27, 102.69, 52.89, 23.55; HRMS-ESI (*m/z*) [M + H]^+^ calculated for C_17_H_14_IN_2_O_2_ 405.00553, found 405.00980.

**Methyl (E)-4-cyano-3-(((2,3-dimethylphenyl)imino)methyl)benzoate (68)**

Brown solid, 83.39 mg, 95% yield; ^1^H NMR (400 MHz, CDCl_3_) *δ* 8.91–8.88 (m, 1H), 8.75 (s, 1H), 8.19 (dd, *J* = 8.0, 1.7 Hz, 1H), 7.84 (d, *J* = 8.1 Hz, 1H), 7.18–7.08 (m, 2H), 6.92–6.85 (m, 1H), 4.00 (s, 3H), 2.35 (d, *J* = 8.9 Hz, 6H); ^13^C NMR (101 MHz, CDCl_3_) *δ* 165.18, 153.66, 149.43, 138.92, 137.80, 134.22, 133.53, 131.42, 131.22, 129.12, 128.66, 126.13, 116.44, 116.31, 115.17, 52.81, 20.06, 13.98; HRMS-ESI (*m/z*) [M + H]^+^ calculated for C_18_H_17_N_2_O_2_ 293.12453, found 293.12836.

**Methyl (E)-4-cyano-3-(((3-methoxy-2-methylphenyl)imino)methyl)benzoate (69)**

Yellow solid, 86.21 mg, 93% yield; ^1^H NMR (400 MHz, CDCl_3_) *δ* 8.92 (d, *J* = 1.6 Hz, 1H), 8.77 (s, 1H), 8.21 (dd, *J* = 8.0, 1.7 Hz, 1H), 7.85 (d, *J* = 8.0 Hz, 1H), 7.21 (t, *J* = 8.1 Hz, 1H), 6.82 (d, *J* = 8.2 Hz, 1H), 6.68 (d, *J* = 7.9 Hz, 1H), 4.00 (s, 3H), 3.88 (s, 3H), 2.31 (s, 3H); ^13^C NMR (101 MHz, CDCl_3_) *δ* 165.19, 158.19, 154.12, 150.27, 138.85, 134.26, 133.50, 131.35, 129.07, 126.71, 121.43, 116.42, 116.39, 110.19, 108.81, 55.68, 52.84, 10.49; HRMS-ESI (*m/z*) [M + H]^+^ calculated for C_18_H_17_N_2_O_3_ 309.11945, found 309.12317.

**Methyl (E)-4-cyano-3-(((2,6-diethylphenyl)imino)methyl)benzoate (70)**

Dark green solid, 85.98 mg, 89% yield; ^1^H NMR (400 MHz, CDCl_3_) *δ* 8.95 (d, *J* = 1.7 Hz, 1H), 8.66 (s, 1H), 8.26 (dd, *J* = 8.1, 1.7 Hz, 1H), 7.87 (d, *J* = 8.1 Hz, 1H), 7.17–7.07 (m, 3H), 4.01 (s, 3H), 2.53 (q, *J* = 7.5 Hz, 4H), 1.17 (t, *J* = 7.5 Hz, 6H); ^13^C NMR (101 MHz, CDCl_3_) *δ* 165.07, 157.52, 149.19, 138.34, 134.38, 133.46, 132.70, 131.74, 128.52, 126.36, 124.80, 116.58, 116.08, 52.84, 24.62, 14.71; HRMS-ESI (*m/z*) [M + H]^+^ calculated for C_20_H_21_N_2_O_2_ 321.15583, found 321.16028.

**Methyl (E)-4-cyano-3-(((4-iodo-3,5-dimethylphenyl)imino)methyl)benzoate (71)**

Brown solid, 117.58 mg, 94% yield; ^1^H NMR (400 MHz, CDCl_3_) *δ* 8.90 (d, *J* = 1.7 Hz, 1H), 8.85 (s, 1H), 8.19 (dd, *J* = 8.0, 1.7 Hz, 1H), 7.82 (d, *J* = 8.0 Hz, 1H), 7.02 (s, 2H), 3.99 (s, 3H), 2.52 (s, 6H); ^13^C NMR (101 MHz, CDCl_3_) *δ* 165.06, 154.32, 149.58, 143.13, 138.55, 134.31, 133.36, 131.53, 128.78, 119.49, 116.59, 116.23, 106.51, 52.82, 29.63; HRMS-ESI (*m/z*) [M + H]^+^ calculated for C_18_H_16_IN_2_O_2_ 419.02118, found 419.02557.

**Methyl (E)-3-(((4-bromo-3,5-dimethylphenyl)imino)methyl)-4-cyanobenzoate (72)**

Orange solid, 103.03 mg, 93% yield; ^1^H NMR (400 MHz, CDCl_3_) *δ* 8.93 (d, *J* = 1.7 Hz, 1H), 8.86 (s, 1H), 8.21 (dd, *J* = 8.1, 1.7 Hz, 1H), 7.86–7.81 (m, 1H), 7.07–7.04 (m, 2H), 4.00 (s, 3H), 2.47 (s, 6H); ^13^C NMR (101 MHz, CDCl_3_) *δ* 165.13, 154.23, 148.57, 139.35, 138.60, 134.35, 133.40, 131.55, 128.79, 126.36, 120.78, 116.61, 116.29, 52.86, 23.94; HRMS-ESI (*m/z*) [M + H]^+^ calculated for C_18_H_16_BrN_2_O_2_ 371.03505, found 371.03852.

**Methyl (E)-4-cyano-3-(((3,4,5-trimethoxyphenyl)imino)methyl)benzoate (73)**

Orange solid, 100.02 mg, 94% yield; ^1^H NMR (400 MHz, CDCl_3_) *δ* 8.90 (d, *J* = 1.7 Hz, 1H), 8.85 (s, 1H), 8.18 (dd, *J* = 8.0, 1.7 Hz, 1H), 7.82 (d, *J* = 8.0 Hz, 1H), 6.59 (s, 2H), 3.98 (s, 3H), 3.91 (s, 6H), 3.87 (s, 3H); ^13^C NMR (101 MHz, CDCl_3_) *δ* 165.08, 153.64, 153.34, 145.94, 138.57, 137.70, 134.29, 133.37, 131.37, 128.71, 116.46, 116.27, 98.65, 60.96, 56.15, 52.79; HRMS-ESI (*m/z*) [M + H]^+^ calculated for C_19_H_19_N_2_O_5_ 355.12493, found 355.12897.

**Methyl (E)-3-(((4-bromo-2,3-dimethylphenyl)imino)methyl)-4-cyanobenzoate (74)**

Yellow solid, 102.45 mg, 92% yield; ^1^H NMR (400 MHz, CDCl_3_) *δ* 8.86 (d, *J* = 1.7 Hz, 1H), 8.71 (s, 1H), 8.20 (dd, *J* = 8.1, 1.7 Hz, 1H), 7.84 (d, *J* = 8.1 Hz, 1H), 7.44 (d, *J* = 8.4 Hz, 1H), 6.76 (d, *J* = 8.4 Hz, 1H), 4.00 (s, 3H), 2.43 (s, 3H), 2.43 (s, 3H). ^13^C NMR (101 MHz, CDCl_3_) *δ* 165.10, 154.01, 148.54, 138.64, 137.21, 134.28, 133.64, 133.44, 131.45, 130.34, 129.23, 124.03, 116.39, 116.32, 116.24, 52.87, 19.82, 15.43; HRMS-ESI (*m/z*) [M + H]^+^ calculated for C_18_H_16_BrN_2_O_2_ 371.03505, found 371.03882.

**Methyl (E)-4-cyano-3-((mesitylimino)methyl)benzoate (75)**

Dark green solid, 83.26 mg, 90% yield; ^1^H NMR (400 MHz, CDCl_3_) *δ* 8.94 (d, *J* = 1.7 Hz, 1H), 8.65 (s, 1H), 8.23 (dd, *J* = 8.1, 1.7 Hz, 1H), 7.85 (d, *J* = 8.0 Hz, 1H), 6.92 (s, 2H), 4.00 (s, 3H), 2.30 (s, 3H), 2.17 (s, 6H); ^13^C NMR (101 MHz, CDCl_3_) *δ* 165.14, 157.87, 147.52, 138.61, 134.31, 134.09, 133.41, 131.59, 128.94, 128.45, 126.87, 116.47, 116.19, 52.83, 20.72, 18.29; HRMS-ESI (*m/z*) [M + H]^+^ calculated for C_19_H_19_N_2_O_2_ 307.14018, found 307.14336.

**Methyl (E)-3-(([1,1'-biphenyl]-2-ylimino)methyl)-4-cyanobenzoate (76)**

Yellow solid, 90.56 mg, 89% yield; ^1^H NMR (400 MHz, CDCl_3_) *δ* 8.86 (s, 1H), 8.74 (d, *J* = 1.7 Hz, 1H), 8.16 (dd, *J* = 8.0, 1.8 Hz, 1H), 7.79 (d, *J* = 8.1 Hz, 1H), 7.56–7.48 (m, 3H), 7.46–7.39 (m, 4H), 7.37–7.34 (m, 1H), 7.22–7.18 (m, 1H), 3.95 (s, 3H); ^13^C NMR (101 MHz, CDCl_3_) *δ* 165.02, 154.74, 148.02, 139.05, 138.75, 136.00, 134.24, 133.16, 131.32, 130.53, 130.14, 128.97, 128.44, 127.74, 127.34, 126.93, 118.57, 116.48, 116.13, 52.68; HRMS-ESI (*m/z*) [M + H]^+^ calculated for C_22_H_17_N_2_O_2_ 341.12453, found 341.12866.

**Methyl (E)-3-(([1,1'-biphenyl]-3-ylimino)methyl)-4-cyanobenzoate (77)**

Orange solid, 93.15 mg, 91% yield; ^1^H NMR (400 MHz, CDCl_3_) *δ* 8.97 (d, *J* = 1.7 Hz, 1H), 8.94 (s, 1H), 8.21 (dd, *J* = 8.1, 1.7 Hz, 1H), 7.84 (d, *J* = 8.1 Hz, 1H), 7.67–7.63 (m, 2H), 7.57–7.53 (m, 3H), 7.50–7.45 (m, 2H), 7.39 (d, *J* = 7.5 Hz, 1H), 7.33–7.28 (m, 1H), 4.00 (s, 3H); ^13^C NMR (101 MHz, CDCl_3_) *δ* 165.10, 154.65, 150.79, 142.48, 140.38, 138.58, 134.30, 133.35, 131.54, 129.70, 128.82, 128.81, 127.60, 127.14, 126.16, 120.18, 119.58, 116.69, 116.23, 52.81; HRMS-ESI (*m/z*) [M + H]^+^ calculated for C_22_H_17_N_2_O_2_ 341.12453, found 341.12802.

**Methyl (E)-3-(([1,1'-biphenyl]-4-ylimino)methyl)-4-cyanobenzoate (78)**

Orange solid, 93.15 mg, 91% yield; ^1^H NMR (400 MHz, CDCl_3_) *δ* 8.97 (d, *J* = 1.7 Hz, 1H), 8.94 (s, 1H), 8.21 (dd, *J* = 8.1, 1.7 Hz, 1H), 7.84 (d, *J* = 8.1 Hz, 1H), 7.70–7.62 (m, 4H), 7.49–7.37 (m, 5H), 4.00 (s, 3H); ^13^C NMR (101 MHz, CDCl_3_) *δ* 165.15, 154.01, 149.25, 140.45, 140.21, 138.69, 134.29, 133.33, 131.45, 128.81, 128.77, 127.98, 127.47, 126.94, 121.76, 116.65, 116.27, 52.82; HRMS-ESI (*m/z*) [M + H]^+^ calculated for C_22_H_17_N_2_O_2_ 341.12453, found 341.12846.

**Methyl (E)-4-cyano-3-(((4-phenoxyphenyl)imino)methyl)benzoate (79)**

Orange solid, 97.51 mg, 91% yield; ^1^H NMR (400 MHz, CDCl_3_) *δ* 8.94 (d, *J* = 1.7 Hz, 1H), 8.89 (s, 1H), 8.19 (dd, *J* = 8.1, 1.7 Hz, 1H), 7.83 (d, *J* = 8.1 Hz, 1H), 7.40–7.33 (m, 4H), 7.18–7.11 (m, 1H), 7.09–7.02 (m, 4H), 4.00 (s, 3H); ^13^C NMR (101 MHz, CDCl_3_) *δ* 165.18, 157.06, 156.86, 153.05, 145.27, 138.79, 134.29, 133.32, 131.30, 129.83, 128.66, 123.59, 122.87, 119.35, 119.05, 116.53, 116.32, 52.83; HRMS-ESI (*m/z*) [M + H]^+^ calculated for C_22_H_17_N_2_O_3_ 357.11945, found 357.12332.

**Methyl (E)-4-cyano-3-(((4-(2,4-difluorophenoxy)phenyl)imino)methyl)benzoate (80)**

Yellow solid, 106.25 mg, 90% yield; ^1^H NMR (400 MHz, CDCl_3_) *δ* 8.93 (d, *J* = 1.7 Hz, 1H), 8.87 (s, 1H), 8.19 (dd, *J* = 8.0, 1.7 Hz, 1H), 7.83 (d, *J* = 8.1 Hz, 1H), 7.37–7.29 (m, 2H), 7.11 (td, *J* = 9.0, 5.5 Hz, 1H), 7.00 (d, *J* = 8.8 Hz, 2H), 6.96 (dd, *J* = 8.0, 2.6 Hz, 1H), 6.89 (dddd, *J* = 9.3, 7.7, 3.1, 1.8 Hz, 1H), 3.99 (s, 3H). ^13^C NMR (101 MHz, CDCl_3_) *δ* 165.19, 157.27, 153.20, 145.28, 138.75, 134.32, 133.35, 131.36, 128.69, 123.01, 122.89, 119.17, 117.36, 116.56, 116.32, 111.62 (d, *J* = 3.9 Hz), 111.39 (d, *J* = 4.0 Hz), 105.59 (d, *J* = 48.8 Hz), 105.59 (d, *J* = 4.8 Hz), 52.85; ^19^F NMR (376 MHz, CDCl_3_) *δ* -114.42 (ddd, *J* = 13.0, 8.1, 4.9 Hz), -125.33 (td, *J* = 10.2, 5.1 Hz); HRMS-ESI (*m/z*) [M + H + H_2_O]^+^ calculated for C_22_H_17_F_2_N_2_O_4_ 411.11564, found 411.11462.

**Methyl (E)-3-(((4-(4-chlorophenoxy)phenyl)imino)methyl)-4-cyanobenzoate (81)**

Red solid, 109.25 mg, 93% yield; ^1^H NMR (400 MHz, CDCl_3_) *δ* 8.93 (d, *J* = 1.7 Hz, 1H), 8.88 (s, 1H), 8.19 (dd, *J* = 8.0, 1.7 Hz, 1H), 7.83 (d, *J* = 8.1 Hz, 1H), 7.33 (dd, *J* = 16.4, 8.9 Hz, 4H), 7.05 (d, *J* = 8.8 Hz, 2H), 6.98 (d, *J* = 9.0 Hz, 2H), 3.99 (s, 3H); ^13^C NMR (101 MHz, CDCl_3_) *δ* 165.14, 156.54, 155.57, 153.36, 145.66, 138.67, 134.28, 133.33, 131.38, 129.80, 128.67, 128.55, 122.93, 120.17, 119.45, 116.54, 116.29, 52.84; HRMS-ESI (*m/z*) [M + H]^+^ calculated for C_22_H_16_ClN_2_O_3_ 391.08047, found 391.08397.

**Methyl (E)-3-(((3-chloro-4-((3-fluorobenzyl)oxy)phenyl)imino)methyl)-4-cyanobenzoate (82)**

Orange solid, 117.58 mg, 93% yield; ^1^H NMR (400 MHz, CDCl_3_) *δ* 8.91 (d, *J* = 1.7 Hz, 1H), 8.85 (s, 1H), 8.19 (dd, *J* = 8.1, 1.7 Hz, 1H), 7.83 (d, *J* = 8.1 Hz, 1H), 7.48 (d, *J* = 2.5 Hz, 1H), 7.37 (td, *J* = 7.9, 5.8 Hz, 1H), 7.26–7.19 (m, 3H), 7.04 (dd, *J* = 8.6, 2.6 Hz, 1H), 7.00 (d, *J* = 8.7 Hz, 1H), 5.19 (s, 2H), 4.00 (s, 3H); ^13^C NMR (101 MHz, CDCl_3_) *δ* 165.14, 162.99 (d, *J* = 246.5 Hz), 153.51, 153.21, 143.83, 138.75 (d, *J* = 7.4 Hz), 138.54, 134.32, 133.40, 131.46, 130.22 (d, *J* = 8.2 Hz), 128.75, 124.01, 123.54, 122.38 (d, *J* = 3.0 Hz), 121.09, 116.52, 116.30, 114.99 (d, *J* = 21.0 Hz), 114.12, 113.93 (d, *J* = 22.3 Hz), 70.21 (d, *J* = 2.2 Hz), 52.86; ^19^F NMR (376 MHz, CDCl_3_) *δ* -112.46 (td, *J* = 9.1, 5.8 Hz); HRMS-ESI (*m/z*) [M + H]^+^ calculated for C_23_H_17_ClFN_2_O_3_ 423.08670, found 423.08958.

**Methyl (E)-3-(((2-benzylphenyl)imino)methyl)-4-cyanobenzoate (83)**

Light yellow solid, 95.87 mg, 90% yield; ^1^H NMR (400 MHz, CDCl_3_) *δ* 8.87 (d, *J* = 1.7 Hz, 1H), 8.71 (s, 1H), 8.20 (dd, *J* = 8.1, 1.7 Hz, 1H), 7.83 (d, *J* = 8.1 Hz, 1H), 7.30 (ddd, *J* = 7.6, 5.4, 3.7 Hz, 2H), 7.24 (t, *J* = 1.5 Hz, 1H), 7.22–7.18 (m, 4H), 7.16–7.10 (m, 1H), 7.07 (dd, *J* = 7.5, 1.0 Hz, 1H), 4.22 (s, 2H), 4.01 (s, 3H); ^13^C NMR (101 MHz, CDCl_3_) *δ* 165.17, 154.03, 148.98, 141.16, 138.72, 136.01, 134.25, 133.48, 131.42, 130.40, 129.08, 128.95, 128.26, 127.51, 127.44, 125.80, 117.54, 116.44, 116.36, 52.88, 37.64; HRMS-ESI (*m/z*) [M + H]^+^ calculated for C_23_H_19_N_2_O_2_ 355.14018, found 355.14387.

**Methyl (E)-4-cyano-3-(((4-tritylphenyl)imino)methyl)benzoate (84)**

Yellow solid, 141.25 mg, 93% yield; ^1^H NMR (400 MHz, CDCl_3_) *δ* 8.93 (d, *J* = 1.7 Hz, 1H), 8.89 (s, 1H), 8.19 (dd, *J* = 8.1, 1.7 Hz, 1H), 7.82 (d, *J* = 8.0 Hz, 1H), 7.32–7.27 (m, 5H), 7.25 (s, 5H), 7.24–7.18 (m, 9H), 3.99 (s, 3H); ^13^C NMR (101 MHz, CDCl_3_) *δ* 165.17, 154.24, 148.00, 146.54, 146.44, 138.74, 134.31, 133.32, 132.10, 131.43, 131.07, 131.03, 128.74, 127.56, 127.43, 126.02, 120.31, 116.66, 116.30, 64.75, 52.84; HRMS-ESI (*m/z*) [M + H]^+^ calculated for C_35_H_27_N_2_O_2_ 507.20278, found 507.20563.

**Methyl (E)-4-cyano-3-(((4-(phenylamino)phenyl)imino)methyl)benzoate (85)**

Brown solid, 96.22 mg, 90% yield; ^1^H NMR (400 MHz, CDCl_3_) *δ* 8.95 (d, *J* = 1.7 Hz, 1H), 8.92 (s, 1H), 8.16 (dd, *J* = 8.1, 1.7 Hz, 1H), 7.81 (d, *J* = 8.1 Hz, 1H), 7.39–7.35 (m, 2H), 7.35–7.29 (m, 2H), 7.16–7.10 (m, 4H), 7.04–6.96 (m, 1H), 4.00 (s, 3H); ^13^C NMR (101 MHz, CDCl_3_) *δ* 165.34, 150.63, 143.50, 142.63, 142.21, 139.32, 134.25, 133.27, 130.84, 129.46, 128.48, 123.13, 121.83, 118.66, 117.44, 116.52, 116.24, 52.80; HRMS-ESI (*m/z*) [M + H]^+^ calculated for C_22_H_18_N_3_O_2_ 356.13543, found 356.13919.

**Methyl (E)-4-cyano-3-(((3-(diphenylamino)phenyl)imino)methyl)benzoate (86)**

Brown solid, 119.23 mg 92% yield; ^1^H NMR (400 MHz, CDCl_3_) *δ* 8.94 (d, *J* = 1.7 Hz, 1H), 8.90 (s, 1H), 8.17 (dd, *J* = 8.1, 1.7 Hz, 1H), 7.82 (d, *J* = 8.1 Hz, 1H), 7.32–7.26 (m, 6H), 7.16–7.11 (m, 6H), 7.06 (td, *J* = 7.3, 1.1 Hz, 2H), 4.00 (s, 3H); ^13^C NMR (101 MHz, CDCl_3_) *δ* 165.25, 151.68, 147.79, 147.35, 144.01, 139.11, 134.24, 133.27, 130.97, 129.35, 128.92, 128.54, 124.66, 123.60, 123.33, 122.61, 122.46, 116.42, 116.32, 52.79; HRMS-ESI (*m/z*) [M + H]^+^ calculated for C_28_H_22_N_3_O_2_ 432.16673, found 432.17028.

**Methyl (E)-4-cyano-3-(((4-(1,2,2-triphenylvinyl)phenyl)imino)methyl)benzoate (87)**

Orange solid, 146.65 mg, 94% yield; ^1^H NMR (400 MHz, CDCl_3_) *δ* 8.91 (d, *J* = 1.7 Hz, 1H), 8.84 (s, 1H), 8.19 (dd, *J* = 8.0, 1.7 Hz, 1H), 7.82 (d, *J* = 8.1 Hz, 1H), 7.17–7.04 (m, 19H), 3.99 (s, 3H). ^13^C NMR (101 MHz, CDCl_3_) *δ* 165.16, 153.68, 148.14, 143.56, 143.53, 143.48, 143.43, 141.48, 140.15, 138.79, 134.30, 133.32, 132.39, 131.36, 131.30, 128.71, 127.83, 127.73, 127.63, 126.65, 126.57, 126.49, 120.71, 116.55, 116.31, 52.81; HRMS-ESI (*m/z*) [M + H]^+^ calculated for C_36_H_27_N_2_O_2_ 519.20278, found 519.20621.

**Methyl (E)-4-cyano-3-(((2-methylnaphthalen-1-yl)imino)methyl)benzoate (88)**

Yellow solid, 92.36 mg, 94% yield; ^1^H NMR (400 MHz, CDCl_3_) *δ* 9.08 (d, *J* = 1.7 Hz, 1H), 8.82 (s, 1H), 8.28 (dd, *J* = 8.1, 1.7 Hz, 1H), 7.89 (d, *J* = 8.1 Hz, 1H), 7.82 (ddd, *J* = 11.4, 8.2, 2.8 Hz, 2H), 7.62 (d, *J* = 8.4 Hz, 1H), 7.47–7.42 (m, 2H), 7.39 (d, *J* = 8.4 Hz, 1H), 4.02 (s, 3H), 2.40 (s, 3H); ^13^C NMR (101 MHz, CDCl_3_) *δ* 165.12, 159.31, 146.19, 138.39, 134.47, 133.58, 132.59, 131.91, 129.16, 128.76, 127.79, 126.32, 126.00, 125.39, 124.75, 122.91, 121.67, 116.69, 116.18, 52.89, 18.30; HRMS-ESI (*m/z*) [M + H]^+^ calculated for C_21_H_17_N_2_O_2_ 329.12453, found 329.12818.

**Methyl (E)-4-cyano-3-((naphthalen-2-ylimino)methyl)benzoate (89)**

Red solid, 89.25 mg, 95% yield; ^1^H NMR (400 MHz, CDCl_3_) *δ* 9.01–8.96 (m, 2H), 8.19 (dd, *J* = 8.1, 1.6 Hz, 1H), 7.91–7.81 (m, 4H), 7.74 (d, *J* = 2.2 Hz, 1H), 7.58–7.45 (m, 3H), 4.00 (s, 3H); ^13^C NMR (101 MHz, CDCl_3_) *δ* 165.09, 154.17, 147.51, 138.63, 134.22, 133.77, 133.30, 132.64, 131.39, 129.22, 128.73, 128.25, 127.70, 126.62, 126.08, 120.20, 119.51, 116.55, 116.29, 52.79; HRMS-ESI (*m/z*) [M + H]^+^ calculated for C_20_H_15_N_2_O_2_ 315.10888, found 315.11285.

**Methyl (E)-4-cyano-3-(((2,3-dihydro-1H-inden-5-yl)imino)methyl)benzoate (90)**

Orange solid, 83.28 mg, 91% yield; ^1^H NMR (400 MHz, CDCl_3_) *δ* 8.94 (d, *J* = 1.7 Hz, 1H), 8.89 (s, 1H), 8.18 (dd, *J* = 8.1, 1.7 Hz, 1H), 7.82 (d, *J* = 8.0 Hz, 1H), 7.30–7.24 (m, 1H), 7.24–7.20 (m, 1H), 7.14 (dd, *J* = 7.9, 2.0 Hz, 1H), 3.99 (s, 3H), 2.95 (q, *J* = 7.6 Hz, 4H), 2.13 (p, *J* = 7.4 Hz, 2H); ^13^C NMR (101 MHz, CDCl_3_) *δ* 165.23, 153.00, 148.64, 145.62, 144.10, 139.01, 134.26, 133.25, 131.16, 128.64, 124.89, 119.86, 116.82, 116.51, 116.35, 52.78, 32.81, 32.52, 25.62; HRMS-ESI (*m/z*) [M + H]^+^ calculated for C_19_H_17_N_2_O_2_ 305.12453, found 305.12781.

**Methyl (E)-4-cyano-3-(((2,3-dihydro-1H-inden-4-yl)imino)methyl)benzoate (91)**

Yellow solid, 83.25 mg, 91% yield; ^1^H NMR (400 MHz, CDCl_3_) *δ* 8.89 (d, *J* = 1.8 Hz, 1H), 8.84 (s, 1H), 8.19 (dd, *J* = 8.0, 1.7 Hz, 1H), 7.84 (d, *J* = 8.0 Hz, 1H), 7.25–7.15 (m, 2H), 6.95 (d, *J* = 6.2 Hz, 1H), 4.00 (s, 3H), 3.07 (t, *J* = 7.4 Hz, 2H), 2.98 (t, *J* = 7.5 Hz, 2H), 2.17–2.07 (m, 2H); ^13^C NMR (101 MHz, CDCl_3_) *δ* 165.20, 154.12, 146.80, 145.91, 138.93, 138.65, 134.23, 133.48, 131.29, 129.02, 127.30, 123.21, 116.41, 116.39, 115.53, 52.82, 33.08, 30.80, 25.15; HRMS-ESI (*m/z*) [M + H]^+^ calculated for C_17_H_14_ClN_2_O_2_ 305.12453, found 305.12836.

**Methyl (E)-4-cyano-3-(((5,6,7,8-tetrahydronaphthalen-1-yl)imino)methyl)benzoate (92)**

Brown solid, 86.98 mg, 91% yield; ^1^H NMR (400 MHz, CDCl_3_) *δ* 8.88 (d, *J* = 1.7 Hz, 1H), 8.74 (s, 1H), 8.19 (dd, *J* = 8.1, 1.7 Hz, 1H), 7.84 (d, *J* = 8.1 Hz, 1H), 7.15 (t, *J* = 7.7 Hz, 1H), 7.03 (d, *J* = 7.6 Hz, 1H), 6.81 (d, *J* = 7.6 Hz, 1H), 4.00 (s, 3H), 2.84 (dt, *J* = 11.6, 5.8 Hz, 4H), 1.90–1.76 (m, 4H); ^13^C NMR (101 MHz, CDCl_3_) *δ* 165.22, 153.51, 149.49, 138.92, 138.27, 134.24, 133.53, 131.85, 131.26, 129.15, 128.04, 125.96, 116.43, 116.35, 114.41, 52.85, 29.66, 25.30, 22.99, 22.89; HRMS-ESI (*m/z*) [M + H]^+^ calculated for C_20_H_19_N_2_O_2_ 319.14018, found 319.14401.

**Methyl (E)-4-cyano-3-(((5,6,7,8-tetrahydronaphthalen-2-yl)imino)methyl)benzoate (93)**

Orange oil, 88.16 mg, 92% yield; ^1^H NMR (400 MHz, CDCl_3_) *δ* 8.93 (d, *J* = 1.8 Hz, 1H), 8.88 (s, 1H), 8.17 (dd, *J* = 8.1, 1.7 Hz, 1H), 7.81 (d, *J* = 8.1 Hz, 1H), 7.15–7.10 (m, 2H), 7.06 (d, *J* = 2.0 Hz, 1H), 3.99 (s, 3H), 2.81 (d, *J* = 7.5 Hz, 4H), 1.85–1.79 (m, 4H); ^13^C NMR (101 MHz, CDCl_3_) *δ* 165.20, 153.02, 147.57, 138.98, 138.16, 137.04, 134.24, 133.25, 131.16, 129.95, 128.62, 121.73, 118.51, 116.48, 116.34, 52.77, 29.42, 29.13, 23.10, 23.00; HRMS-ESI (*m/z*) [M + H]^+^ calculated for C_20_H_19_N_2_O_2_ 319.14018, found 319.14389.

**Methyl (E)-4-cyano-3-(((9,9-dimethyl-9H-fluoren-2-yl)imino)methyl)benzoate (94)**

Orange solid, 106.31 mg, 93% yield; ^1^H NMR (400 MHz, CDCl_3_) *δ* 8.99 (d, *J* = 1.4 Hz, 2H), 8.21 (dd, *J* = 8.0, 1.7 Hz, 1H), 7.85 (d, *J* = 8.1 Hz, 1H), 7.78 (d, *J* = 8.0 Hz, 1H), 7.76–7.73 (m, 1H), 7.48–7.43 (m, 2H), 7.39–7.30 (m, 3H), 4.01 (s, 3H), 1.54 (s, 6H); ^13^C NMR (101 MHz, CDCl_3_) *δ* 165.20, 155.00, 153.93, 153.22, 149.34, 139.01, 138.89, 138.45, 134.32, 133.40, 131.33, 128.78, 127.45, 127.10, 122.63, 120.73, 120.24, 120.10, 116.50, 116.40, 116.20, 52.83, 46.99, 27.12; HRMS-ESI (*m/z*) [M + H]^+^ calculated for C_25_H_21_N_2_O_2_ 381.15583, found 381.15869.

**Methyl (E)-3-(((7-bromo-9H-fluoren-2-yl)imino)methyl)-4-cyanobenzoate (95)**

Brown solid, 129.39 mg, 93% yield; ^1^H NMR (400 MHz, CDCl_3_) *δ* 8.98 (d, *J* = 1.7 Hz, 1H), 8.96 (s, 1H), 8.21 (dd, *J* = 8.1, 1.7 Hz, 1H), 7.85 (d, *J* = 8.0 Hz, 1H), 7.80 (d, *J* = 8.1 Hz, 1H), 7.70 (d, *J* = 1.7 Hz, 1H), 7.65 (d, *J* = 8.2 Hz, 1H), 7.55–7.49 (m, 2H), 7.40 (dd, *J* = 8.1, 1.9 Hz, 1H), 4.01 (s, 3H), 3.95 (s, 2H); ^13^C NMR (101 MHz, CDCl_3_) *δ* 165.20, 153.55, 149.31, 145.50, 144.22, 140.41, 140.05, 138.81, 134.36, 133.38, 131.42, 130.10, 128.79, 128.33, 121.22, 121.14, 120.80, 120.63, 117.80, 116.60, 116.38, 52.87, 36.78; HRMS-ESI (*m/z*) [M + H]^+^ calculated for C_23_H_16_BrN_2_O_2_ 431.03505, found 431.03861.

**Methyl (E)-4-cyano-3-(((2-methyl-1H-indol-5-yl)imino)methyl)benzoate (96)**

Brown solid, 85.99 mg, 90% yield; ^1^H NMR (400 MHz, CDCl_3_) *δ* 9.00 (d, *J* = 3.6 Hz, 2H), 8.16 (dd, *J* = 8.1, 1.7 Hz, 1H), 8.01 (s, 1H), 7.82 (d, *J* = 8.1 Hz, 1H), 7.58 (d, *J* = 2.0 Hz, 1H), 7.33 (d, *J* = 8.5 Hz, 1H), 7.26–7.23 (m, 1H), 6.29 (dt, *J* = 2.1, 1.1 Hz, 1H), 4.00 (s, 3H), 2.47 (s, 3H); ^13^C NMR (101 MHz, CDCl_3_) *δ* 165.40, 150.89, 142.70, 139.48, 136.57, 135.92, 134.17, 133.21, 130.66, 129.63, 128.44, 116.60, 116.16, 115.52, 112.78, 110.74, 101.22, 52.76, 13.76; HRMS-ESI (*m/z*) [M + H]^+^ calculated for C_19_H_16_N_3_O_2_ 318.11978, found 318.12347.

**Methyl (E)-3-(((1H-indol-6-yl)imino)methyl)-4-cyanobenzoate (97)**

Brown solid, 83.25 mg, 91% yield; ^1^H NMR (400 MHz, CDCl_3_) *δ* 9.01 (s, 1H), 8.99 (d, *J* = 1.7 Hz, 1H), 8.31 (s, 1H), 8.18 (dd, *J* = 8.0, 1.7 Hz, 1H), 7.83 (dd, *J* = 8.1, 0.5 Hz, 1H), 7.68 (d, *J* = 8.4 Hz, 1H), 7.46 (dd, *J* = 1.8, 0.9 Hz, 1H), 7.29–7.27 (m, 1H), 7.25 (d, *J* = 1.8 Hz, 1H), 6.59 (td, *J* = 2.1, 1.0 Hz, 1H), 4.01 (s, 3H); ^13^C NMR (101 MHz, CDCl_3_) *δ* 165.35, 151.93, 144.95, 139.30, 136.24, 134.29, 133.29, 130.92, 128.64, 127.99, 125.70, 121.28, 116.53, 116.34, 114.13, 104.85, 102.98, 52.79; HRMS-ESI (*m/z*) [M + H]^+^ calculated for C_18_H_14_N_3_O_2_ 304.10413, found 304.10834.

**Methyl (E)-3-(((1H-indol-4-yl)imino)methyl)-4-cyanobenzoate (98)**

Dark green solid, 83.02 mg, 91% yield; ^1^H NMR (400 MHz, CDCl_3_) *δ* 9.11 (s, 1H), 9.03 (d, *J* = 1.7 Hz, 1H), 8.36 (s, 1H), 8.21 (dd, *J* = 8.1, 1.7 Hz, 1H), 7.86 (d, *J* = 8.1 Hz, 1H), 7.36 (dt, *J* = 8.2, 0.9 Hz, 1H), 7.29 (dd, *J* = 3.2, 2.4 Hz, 1H), 7.23 (d, *J* = 8.0 Hz, 1H), 7.01 (dd, *J* = 7.5, 0.8 Hz, 1H), 6.87 (ddd, *J* = 3.2, 2.1, 1.0 Hz, 1H), 4.01 (s, 3H); ^13^C NMR (101 MHz, CDCl_3_) *δ* 165.32, 154.04, 143.10, 139.28, 136.99, 134.23, 133.45, 131.12, 128.99, 124.81, 122.99, 122.47, 116.59, 116.32, 110.55, 110.01, 100.87, 52.80; HRMS-ESI (*m/z*) [M + H]^+^ calculated for C_18_H_14_N_3_O_2_ 304.10413, found 304.10757.

**Methyl (E)-4-cyano-3-(((1-methyl-1H-indol-5-yl)imino)methyl)benzoate (99)**

Brown solid, 87.23 mg, 92% yield; ^1^H NMR (400 MHz, CDCl_3_) *δ* 9.01 (s, 1H), 9.00 (d, *J* = 1.7 Hz, 1H), 8.16 (dd, *J* = 8.1, 1.7 Hz, 1H), 7.82 (d, *J* = 8.1 Hz, 1H), 7.69 (t, *J* = 1.4 Hz, 1H), 7.37 (d, *J* = 1.3 Hz, 2H), 7.10 (d, *J* = 3.1 Hz, 1H), 6.55 (d, *J* = 3.1 Hz, 1H), 4.00 (s, 3H), 3.83 (s, 3H); ^13^C NMR (101 MHz, CDCl_3_) *δ* 165.39, 151.05, 142.48, 139.46, 136.51, 134.20, 133.22, 130.72, 130.08, 128.92, 128.46, 116.59, 116.22, 116.15, 114.07, 109.80, 101.88, 52.78, 33.07; HRMS-ESI (*m/z*) [M + H]^+^ calculated for C_19_H_16_N_3_O_2_ 318.11978, found 318.12329.

**Methyl (E)-3-(((1-benzyl-1H-indol-5-yl)imino)methyl)-4-cyanobenzoate (100)**

Red oil, 111.35 mg, 94% yield; ^1^H NMR (400 MHz, CDCl_3_) *δ* 9.01–8.97 (m, 2H), 8.16 (dd, *J* = 8.1, 1.7 Hz, 1H), 7.81 (d, *J* = 8.1 Hz, 1H), 7.73–7.69 (m, 1H), 7.34–7.27 (m, 5H), 7.18 (d, *J* = 3.2 Hz, 1H), 7.16–7.10 (m, 2H), 6.62 (d, *J* = 3.2 Hz, 1H), 5.35 (s, 2H), 4.00 (s, 3H); ^13^C NMR (101 MHz, CDCl_3_) *δ* 165.39, 151.26, 142.77, 139.42, 137.14, 136.09, 134.20, 133.23, 130.76, 129.56, 129.22, 128.82, 128.49, 127.74, 126.73, 116.56, 116.35, 116.27, 114.10, 110.32, 102.62, 52.79, 50.33; HRMS-ESI (*m/z*) [M + H]^+^ calculated for C_25_H_20_N_3_O_2_ 394.15108, found 394.15479.

**Tert-butyl (E)-6-((2-cyano-5-(methoxycarbonyl)benzylidene)amino)-1H-indole-1-carboxylate (101)**

Red-brown solid, 111.35 mg, 92% yield; ^1^H NMR (400 MHz, CDCl_3_) *δ* 8.98 (s, 1H), 8.97 (d, *J* = 1.8 Hz, 1H), 8.22–8.15 (m, 2H), 7.82 (d, *J* = 8.0 Hz, 1H), 7.63 (d, *J* = 3.7 Hz, 1H), 7.59 (d, *J* = 8.2 Hz, 1H), 7.31 (dd, *J* = 8.3, 1.9 Hz, 1H), 6.62–6.56 (m, 1H), 4.00 (s, 3H), 1.70 (s, 9H); ^13^C NMR (101 MHz, CDCl_3_) *δ* 165.22, 152.90, 149.52, 146.80, 138.92, 134.19, 133.29, 131.13, 130.18, 128.72, 127.02, 121.35, 117.42, 116.42, 116.36, 112.14, 107.93, 107.17, 84.10, 52.78, 28.15; HRMS-ESI (*m/z*) [M + H]^+^ calculated for C_23_H_22_N_3_O_4_ 404.15656, found 404.15973.

**Tert-butyl (E)-6-((2-cyano-5-(methoxycarbonyl)benzylidene)amino)indoline-1-carboxylate (102)**

Red-brown solid, 109.25 mg, 90% yield; ^1^H NMR (400 MHz, CDCl_3_) *δ* 8.90 (d, *J* = 12.1 Hz, 2H), 8.18 (dd, *J* = 8.1, 1.7 Hz, 1H), 7.82 (d, *J* = 8.1 Hz, 1H), 7.26 (s, 1H), 7.18 (d, *J* = 7.8 Hz, 1H), 6.94 (dd, *J* = 7.8, 2.0 Hz, 1H), 4.09–4.00 (m, 2H), 3.99 (s, 3H), 3.12 (t, *J* = 8.6 Hz, 2H), 1.57 (s, 9H); ^13^C NMR (101 MHz, CDCl_3_) *δ* 165.21, 152.47, 138.86, 134.23, 133.34, 131.27, 128.85, 116.31, 52.80, 48.10, 28.44; HRMS-ESI (*m/z*) [M + H]^+^ calculated for C_23_H_24_N_3_O_4_ 406.17221, found 406.17544.

**Methyl (E)-4-cyano-3-(((1-methyl-2-oxoindolin-5-yl)imino)methyl)benzoate (103)**

Yellow solid, 92.15 mg, 92% yield; ^1^H NMR (400 MHz, CDCl_3_) *δ* 8.94 (d, *J* = 1.7 Hz, 1H), 8.91 (s, 1H), 8.18 (dd, *J* = 8.1, 1.7 Hz, 1H), 7.83 (d, *J* = 8.1 Hz, 1H), 7.36 (dt, *J* = 8.3, 1.1 Hz, 2H), 6.88 (d, *J* = 8.9 Hz, 1H), 4.00 (s, 3H), 3.60 (s, 2H), 3.26 (s, 3H); ^13^C NMR (101 MHz, CDCl_3_) *δ* 174.91, 165.21, 152.00, 145.06, 144.94, 138.89, 134.33, 133.34, 131.21, 128.63, 125.60, 122.22, 117.66, 116.41, 108.47, 52.85, 35.76, 26.37; HRMS-ESI (*m/z*) [M + H]^+^ calculated for C_19_H_16_N_3_O_3_ 334.11470, found 334.11837.

**Methyl (E)-4-cyano-3-(((1-methyl-1H-indazol-5-yl)imino)methyl)benzoate (104)**

Brown solid, 88.23 mg, 92% yield; ^1^H NMR (400 MHz, CDCl_3_) *δ* 8.99–8.95 (m, 2H), 8.18 (dd, *J* = 8.0, 1.7 Hz, 1H), 8.03 (d, *J* = 0.9 Hz, 1H), 7.83 (d, *J* = 8.1 Hz, 1H), 7.72 (d, *J* = 1.9 Hz, 1H), 7.52 (dd, *J* = 8.8, 1.9 Hz, 1H), 7.44 (d, *J* = 9.0 Hz, 1H), 4.11 (s, 3H), 4.00 (s, 3H); ^13^C NMR (101 MHz, CDCl_3_) *δ* 165.22, 152.52, 143.54, 139.36, 138.93, 134.28, 133.52, 133.31, 131.14, 128.64, 124.45, 121.22, 116.41, 116.40, 113.48, 109.70, 52.81, 35.72; HRMS-ESI (*m/z*) [M + H]^+^ calculated for C_18_H_15_N_4_O_2_ 319.11503, found 319.11829.

**Methyl (E)-3-(((3-bromo-1-methyl-1H-indazol-5-yl)imino)methyl)-4-cyanobenzoate (105)**

Orange solid, 111.23 mg, 93% yield; ^1^H NMR (400 MHz, CDCl_3_) *δ* 8.97 (s, 1H), 8.95 (d, *J* = 1.6 Hz, 1H), 8.20 (dd, *J* = 8.1, 1.6 Hz, 1H), 7.85 (d, *J* = 8.0 Hz, 1H), 7.55 (d, *J* = 2.0 Hz, 1H), 7.53 (d, *J* = 1.9 Hz, 1H), 7.42 (d, *J* = 9.7 Hz, 1H), 4.08 (s, 3H), 4.00 (s, 3H); ^13^C NMR (101 MHz, CDCl_3_) *δ* 165.16, 153.29, 144.27, 140.44, 138.68, 134.32, 133.42, 131.40, 128.78, 124.22, 122.67, 120.59, 116.46, 116.39, 112.32, 110.11, 52.87, 36.17; HRMS-ESI (*m/z*) [M + H]^+^ calculated for C_18_H_14_BrN_4_O_2_ 397.02554, found 397.02882.

**Methyl (E)-4-cyano-3-(((1-methyl-1H-indazol-7-yl)imino)methyl)benzoate (106)**

Yellow solid, 88.45 mg, 93% yield; ^1^H NMR (400 MHz, CDCl_3_) *δ* 8.99 (s, 1H), 8.93 (d, *J* = 1.7 Hz, 1H), 8.23 (dd, *J* = 8.1, 1.7 Hz, 1H), 8.00 (s, 1H), 7.89 (d, *J* = 8.0 Hz, 1H), 7.70–7.67 (m, 1H), 7.18–7.10 (m, 2H), 4.41 (s, 3H), 4.01 (s, 3H); ^13^C NMR (101 MHz, CDCl_3_) *δ* 165.05, 154.03, 138.36, 135.98, 134.82, 134.42, 133.80, 132.78, 131.61, 129.18, 126.11, 121.16, 120.83, 116.45, 116.34, 113.78, 52.98, 39.42; HRMS-ESI (*m/z*) [M + H]^+^ calculated for C_18_H_15_N_4_O_2_ 319.11503, found 319.11853.

**Methyl (E)-4-cyano-3-(((1-methyl-1H-indazol-6-yl)imino)methyl)benzoate (107)**

Yellow solid, 90.36 mg, 95% yield; ^1^H NMR (400 MHz, CDCl_3_) *δ* 8.97 (s, 2H), 8.22 (dd, *J* = 8.1, 1.7 Hz, 1H), 7.99 (d, *J* = 1.1 Hz, 1H), 7.85 (d, *J* = 8.0 Hz, 1H), 7.76 (d, *J* = 8.5 Hz, 1H), 7.30 (s, 1H), 7.18 (dd, *J* = 8.6, 1.7 Hz, 1H), 4.11 (s, 3H), 4.00 (s, 3H); ^13^C NMR (101 MHz, CDCl_3_) *δ* 165.13, 154.63, 148.89, 140.46, 138.58, 134.39, 133.45, 132.84, 131.59, 128.90, 123.24, 121.91, 116.66, 116.32, 114.97, 101.47, 52.86, 35.66; HRMS-ESI (*m/z*) [M + H]^+^ calculated for C_18_H_15_N_4_O_2_ 319.11503, found 319.11832.

**Methyl (E)-4-cyano-3-((quinolin-3-ylimino)methyl)benzoate (108)**

Yellow solid, 85.39 mg, 90% yield; ^1^H NMR (400 MHz, CDCl_3_) *δ* 9.00 (s, 1H), 8.97 (dd, *J* = 3.7, 2.1 Hz, 2H), 8.22 (dd, *J* = 8.1, 1.7 Hz, 1H), 8.15–8.09 (m, 1H), 7.97 (d, *J* = 2.5 Hz, 1H), 7.88–7.82 (m, 2H), 7.70 (ddd, *J* = 8.4, 6.9, 1.5 Hz, 1H), 7.57 (ddd, *J* = 8.1, 6.8, 1.2 Hz, 1H), 3.99 (s, 3H); ^13^C NMR (101 MHz, CDCl_3_) *δ* 164.94, 156.27, 147.22, 146.44, 143.19, 138.04, 134.38, 133.45, 131.97, 129.41, 129.22, 128.97, 128.11, 128.03, 127.36, 124.36, 116.76, 116.10, 52.88; HRMS-ESI (*m/z*) [M + H]^+^ calculated for C_19_H_14_N_3_O_2_ 316.10413, found 316.10858.

**Methyl (E)-4-cyano-3-(((1-methyl-2-oxo-1,2,3,4-tetrahydroquinolin-6-yl)imino)methyl)benzoate (109)**

Yellow solid, 96.19 mg, 92% yield; ^1^H NMR (400 MHz, CDCl_3_) *δ* 8.93 (d, *J* = 1.7 Hz, 1H), 8.89 (s, 1H), 8.19 (dd, *J* = 8.1, 1.7 Hz, 1H), 7.83 (d, *J* = 8.0 Hz, 1H), 7.29 (dd, *J* = 8.5, 2.5 Hz, 1H), 7.21 (d, *J* = 2.4 Hz, 1H), 7.04 (d, *J* = 8.6 Hz, 1H), 3.99 (s, 3H), 3.39 (s, 3H), 3.00–2.93 (m, 2H), 2.73–2.66 (m, 2H); ^13^C NMR (101 MHz, CDCl_3_) *δ* 170.24, 165.15, 152.89, 144.77, 140.22, 138.74, 134.29, 133.35, 131.32, 128.64, 127.22, 120.78, 116.45, 116.34, 115.39, 52.84, 31.50, 29.69, 25.35; HRMS-ESI (*m/z*) [M + H]^+^ calculated for C_20_H_18_N_3_O_3_ 348.13035, found 348.13409.

**Tert-butyl (E)-6-((2-cyano-5-(methoxycarbonyl)benzylidene)amino)-3,4-dihydroisoquinoline-2(1H)-carboxylate (110)**

Red oil, 113.24 mg, 90% yield; ^1^H NMR (400 MHz, CDCl_3_) *δ* 8.93 (d, *J* = 1.7 Hz, 1H), 8.87 (s, 1H), 8.19 (dd, *J* = 8.0, 1.7 Hz, 1H), 7.83 (d, *J* = 8.1 Hz, 1H), 7.17 (d, *J* = 1.4 Hz, 2H), 7.11 (s, 1H), 4.60 (s, 2H), 3.99 (s, 3H), 3.67 (t, *J* = 5.9 Hz, 2H), 2.88 (t, *J* = 5.9 Hz, 2H), 1.50 (s, 9H); ^13^C NMR (101 MHz, CDCl_3_) *δ* 165.12, 154.84, 153.92, 148.44, 138.67, 135.90, 134.28, 133.32, 131.42, 128.71, 127.25, 119.28, 116.58, 116.27, 79.88, 52.82, 29.04, 28.44; HRMS-ESI (*m/z*) [M + H]^+^ calculated for C_24_H_26_N_3_O_4_ 420.18786, found 420.19120.

**Methyl (E)-4-cyano-3-(((1,3-dimethyl-2-oxo-2,3-dihydro-1H-benzo[d]imidazol-5-yl)imino)methyl)benzoate (111)**

Yellow solid, 96.15 mg, 92% yield; ^1^H NMR (400 MHz, CDCl_3_) *δ* 8.97–8.92 (m, 2H), 8.18 (dd, *J* = 8.1, 1.7 Hz, 1H), 7.83 (d, *J* = 8.1 Hz, 1H), 7.19 (dd, *J* = 8.3, 1.9 Hz, 1H), 7.06 (d, *J* = 1.9 Hz, 1H), 7.01 (d, *J* = 8.3 Hz, 1H), 4.00 (s, 3H), 3.47 (s, 3H), 3.46 (s, 3H); ^13^C NMR (101 MHz, CDCl_3_) *δ* 165.21, 154.84, 152.02, 144.21, 138.91, 134.32, 133.39, 131.17, 130.78, 129.97, 128.66, 116.46, 116.33, 115.32, 107.63, 101.07, 52.84, 27.34; HRMS-ESI (*m/z*) [M + H]^+^ calculated for C_19_H_17_N_4_O_3_ 349.12560, found 349.12921.

**Tert-butyl (E)-4-(4-((2-cyano-5-(methoxycarbonyl)benzylidene)amino)phenoxy)piperidine-1-carboxylate (112)**

Tan solid, 128.33 mg, 92% yield; ^1^H NMR (400 MHz, CDCl_3_) *δ* 8.92 (d, *J* = 1.7 Hz, 1H), 8.88 (s, 1H), 8.16 (dd, *J* = 8.0, 1.7 Hz, 1H), 7.81 (d, *J* = 8.0 Hz, 1H), 7.35 (d, *J* = 8.7 Hz, 2H), 6.96 (d, *J* = 8.8 Hz, 2H), 4.51 (dt, *J* = 7.2, 3.7 Hz, 1H), 3.98 (s, 3H), 3.71 (ddd, *J* = 12.1, 7.3, 3.7 Hz, 2H), 3.36 (ddd, *J* = 13.7, 7.8, 3.8 Hz, 2H), 1.94 (td, *J* = 8.1, 3.7 Hz, 2H), 1.81–1.73 (m, 2H), 1.47 (s, 9H); ^13^C NMR (101 MHz, CDCl_3_) *δ* 165.20, 157.12, 154.79, 151.85, 143.25, 139.01, 134.24, 133.26, 131.04, 128.52, 122.96, 116.63, 116.37, 116.34, 79.60, 72.47, 52.77, 30.44, 28.39; HRMS-ESI (*m/z*) [M + H]^+^ calculated for C_26_H_30_N_3_O_5_ 464.21408, found 464.21756.

**Tert-butyl (E)-3-(4-((2-cyano-5-(methoxycarbonyl)benzylidene)amino)phenoxy)piperidine-1-carboxylate (113)**

Brown solid, 128.11 mg, 92% yield; ^1^H NMR (400 MHz, CDCl_3_) *δ* 8.93 (d, *J* = 1.7 Hz, 1H), 8.88 (s, 1H), 8.17 (dd, *J* = 8.1, 1.7 Hz, 1H), 7.81 (d, *J* = 8.1 Hz, 1H), 7.35 (d, *J* = 8.8 Hz, 2H), 6.97 (d, *J* = 8.9 Hz, 2H), 4.51 (dt, *J* = 7.2, 3.6 Hz, 1H), 3.99 (s, 3H), 3.71 (ddd, *J* = 12.2, 7.4, 3.7 Hz, 2H), 3.36 (ddd, *J* = 13.5, 7.7, 3.8 Hz, 2H), 2.00–1.90 (m, 2H), 1.83–1.72 (m, 2H), 1.47 (s, 9H); ^13^C NMR (101 MHz, CDCl_3_) *δ* 165.23, 157.11, 154.82, 151.87, 143.22, 139.00, 134.23, 133.28, 131.07, 128.52, 122.98, 116.62, 116.39, 116.35, 79.65, 72.43, 52.81, 30.42, 28.39; HRMS-ESI (*m/z*) [M + H]^+^ calculated for C_26_H_30_N_3_O_5_ 464.21408, found 464.21756.

**Methyl (E)-3-(((3-chloro-4-(4-methylpiperidin-1-yl)phenyl)imino)methyl)-4-cyanobenzoate (114)**

Brown solid, 106.52 mg, 90% yield; ^1^H NMR (400 MHz, CDCl_3_) *δ* 8.91 (d, *J* = 1.7 Hz, 1H), 8.85 (s, 1H), 8.18 (dd, *J* = 8.0, 1.7 Hz, 1H), 7.82 (d, *J* = 8.0 Hz, 1H), 7.43 (d, *J* = 2.5 Hz, 1H), 7.27 (s, 1H), 7.08 (d, *J* = 8.6 Hz, 1H), 3.99 (s, 3H), 3.41 (d, *J* = 12.0 Hz, 2H), 2.67 (t, *J* = 11.2 Hz, 2H), 1.79–1.72 (m, 2H), 1.55–1.40 (m, 3H), 1.01 (d, *J* = 5.8 Hz, 3H); ^13^C NMR (101 MHz, CDCl_3_) *δ* 165.24, 152.84, 150.08, 144.71, 138.78, 134.34, 133.41, 131.37, 129.28, 128.74, 123.77, 120.90, 120.69, 116.52, 116.38, 52.90, 52.19, 34.50, 30.69, 21.98; HRMS-ESI (*m/z*) [M + H]^+^ calculated for C_22_H_23_ClN_3_O_2_ 396.14341, found 396.14694.

**Methyl (E)-4-cyano-3-(((4-(3-methyl-2-oxoimidazolidin-1-yl)phenyl)imino)methyl)benzoate (115)**

Yellow solid, 100.65 mg, 93% yield; ^1^H NMR (400 MHz, CDCl_3_) *δ* 8.94 (d, *J* = 1.7 Hz, 1H), 8.91 (s, 1H), 8.17 (dd, *J* = 8.1, 1.7 Hz, 1H), 7.82 (d, *J* = 8.1 Hz, 1H), 7.67–7.63 (m, 2H), 7.42–7.38 (m, 2H), 3.99 (s, 3H), 3.88–3.83 (m, 2H), 3.54–3.48 (m, 2H), 2.92 (s, 3H); ^13^C NMR (101 MHz, CDCl_3_) *δ* 165.28, 157.93, 152.12, 144.02, 140.53, 139.07, 134.27, 133.31, 131.10, 128.62, 122.22, 117.65, 116.43, 116.39, 52.82, 43.99, 42.33, 31.22; HRMS-ESI (*m/z*) [M + H]^+^ calculated for C_20_H_19_N_4_O_3_ 363.14125, found 363.14517.

**Tert-butyl (E)-4-(4-((2-cyano-5-(methoxycarbonyl)benzylidene)amino)phenyl)piperazine-1-carboxylate (116)**

Dark brown solid, 125.11 mg, 93% yield; ^1^H NMR (400 MHz, CDCl_3_) *δ* 8.93 (d, *J* = 1.7 Hz, 1H), 8.90 (s, 1H), 8.14 (dd, *J* = 8.1, 1.7 Hz, 1H), 7.80 (d, *J* = 8.1 Hz, 1H), 7.37 (d, *J* = 8.9 Hz, 2H), 6.96 (d, *J* = 9.0 Hz, 2H), 3.98 (s, 3H), 3.60 (t, *J* = 5.2 Hz, 4H), 3.21 (t, *J* = 5.2 Hz, 4H), 1.49 (s, 9H); ^13^C NMR (101 MHz, CDCl_3_) *δ* 165.29, 154.66, 150.95, 150.69, 141.87, 139.26, 134.19, 133.23, 130.80, 128.41, 122.88, 116.48, 116.46, 116.17, 80.00, 52.77, 48.85, 28.39; HRMS-ESI (*m/z*) [M + H]^+^ calculated for C_25_H_29_N_4_O_4_ 449.21441, found 449.21776.

**Methyl (E)-4-cyano-3-(((4-(1-methyl-1H-pyrazol-4-yl)phenyl)imino)methyl)benzoate (117)**

Red-brown solid, 94.15 mg, 91% yield; ^1^H NMR (400 MHz, CDCl_3_) *δ* 8.96 (d, *J* = 1.7 Hz, 1H), 8.93 (s, 1H), 8.20 (dd, *J* = 8.0, 1.7 Hz, 1H), 7.84 (d, *J* = 8.1 Hz, 1H), 7.80 (s, 1H), 7.66 (s, 1H), 7.55 (d, *J* = 8.5 Hz, 2H), 7.37 (d, *J* = 8.5 Hz, 2H), 4.00 (s, 3H), 3.97 (s, 3H); ^13^C NMR (101 MHz, CDCl_3_) *δ* 165.18, 153.33, 148.20, 138.82, 136.71, 134.32, 133.32, 132.21, 131.33, 128.74, 127.00, 126.25, 122.54, 122.02, 116.57, 116.30, 52.80, 39.08; HRMS-ESI (*m/z*) [M + H]^+^ calculated for C_20_H_17_N_4_O_2_ 345.13068, found 345.13394.

**Methyl (E)-3-(((4-((1H-pyrazol-1-yl)methyl)phenyl)imino)methyl)-4-cyanobenzoate (118)**

Orange solid, 94.22 mg, 91% yield; ^1^H NMR (400 MHz, CDCl_3_) *δ* 8.92 (d, *J* = 1.7 Hz, 1H), 8.84 (s, 1H), 8.20 (dd, *J* = 8.0, 1.7 Hz, 1H), 7.83 (d, *J* = 8.1 Hz, 1H), 7.57 (d, *J* = 1.9 Hz, 1H), 7.42 (d, *J* = 2.3 Hz, 1H), 7.28 (s, 4H), 6.30 (t, *J* = 2.1 Hz, 1H), 5.37 (s, 2H), 3.99 (s, 3H); ^13^C NMR (101 MHz, CDCl_3_) *δ* 165.09, 154.71, 150.05, 139.65, 138.48, 135.86, 134.31, 133.34, 131.58, 129.27, 128.80, 128.64, 121.56, 116.72, 116.18, 106.08, 55.39, 52.83. HRMS-ESI (*m/z*) [M + H]^+^ calculated for C_20_H_17_N_4_O_2_ 345.13068, found 345.13413.

**Methyl (E)-4-cyano-3-(((4-(1-methyl-1H-pyrazol-3-yl)phenyl)imino)methyl)benzoate (119)**

Orange solid, 95.10 mg, 92% yield; ^1^H NMR (400 MHz, CDCl_3_) *δ* 8.97 (d, *J* = 1.7 Hz, 1H), 8.94 (s, 1H), 8.20 (dd, *J* = 8.1, 1.7 Hz, 1H), 7.90–7.86 (m, 2H), 7.84 (d, *J* = 8.1 Hz, 1H), 7.42–7.37 (m, 3H), 6.58 (d, *J* = 2.3 Hz, 1H), 4.00 (s, 3H), 3.98 (s, 3H); ^13^C NMR (101 MHz, CDCl_3_) *δ* 165.20, 153.58, 150.84, 149.24, 138.83, 134.32, 133.35, 133.05, 131.50, 131.39, 128.79, 126.42, 121.73, 116.61, 116.34, 102.99, 52.83, 39.09; HRMS-ESI (*m/z*) [M + H]^+^ calculated for C_20_H_17_N_4_O_2_ 345.13068, found 345.13434.

**Methyl (E)-4-cyano-3-(((4-(pyridin-2-ylmethoxy)phenyl)imino)methyl)benzoate (120)**

Orange solid, 102.66 mg, 92% yield; ^1^H NMR (400 MHz, CDCl_3_) *δ* 8.91 (d, *J* = 1.7 Hz, 1H), 8.85 (s, 1H), 8.61 (d, *J* = 4.5 Hz, 1H), 8.19 (dd, *J* = 8.1, 1.8 Hz, 1H), 7.83 (d, *J* = 8.1 Hz, 1H), 7.78 (td, *J* = 7.7, 1.7 Hz, 1H), 7.67 (d, *J* = 7.9 Hz, 1H), 7.49 (d, *J* = 2.5 Hz, 1H), 7.29–7.24 (m, 3H), 7.05 (d, *J* = 8.8 Hz, 1H), 5.34 (s, 2H), 4.00 (s, 3H); ^13^C NMR (101 MHz, CDCl_3_) *δ* 165.12, 156.36, 153.40, 153.20, 148.95, 143.79, 138.54, 137.22, 134.31, 133.38, 131.45, 128.73, 123.70, 123.47, 122.88, 121.25, 121.21, 116.52, 116.28, 113.93, 71.32, 52.85; HRMS-ESI (*m/z*) [M + H]^+^ calculated for C_22_H_18_N_3_O_3_ 372.13035, found 372.13494.

**Methyl (E)-3-(((3-chloro-4-(pyridin-2-ylmethoxy)phenyl)imino)methyl)-4-cyanobenzoate (121)**

Yellow solid, 113.21 mg, 93% yield; ^1^H NMR (400 MHz, CDCl_3_) *δ* 8.90 (d, *J* = 1.7 Hz, 1H), 8.84 (s, 1H), 8.60 (dt, *J* = 5.0, 1.3 Hz, 1H), 8.19 (dd, *J* = 8.1, 1.7 Hz, 1H), 7.82 (d, *J* = 8.0 Hz, 1H), 7.77 (dd, *J* = 7.7, 1.8 Hz, 1H), 7.67 (d, *J* = 7.9 Hz, 1H), 7.48 (d, *J* = 2.5 Hz, 1H), 7.29–7.26 (m, 1H), 7.24 (d, *J* = 2.5 Hz, 1H), 7.04 (d, *J* = 8.7 Hz, 1H), 5.33 (s, 2H), 3.99 (s, 3H); ^13^C NMR (101 MHz, CDCl_3_) *δ* 165.12, 156.32, 153.38, 153.21, 148.86, 143.81, 138.54, 137.30, 134.31, 133.37, 131.45, 128.73, 123.71, 123.46, 122.90, 121.29, 121.21, 116.52, 116.28, 113.94, 71.27, 52.85; HRMS-ESI (*m/z*) [M + H]^+^ calculated for C_22_H_17_ClN_3_O_3_ 406.09137, found 406.09531.

**Methyl (E)-4-cyano-3-(((3-(pyridin-2-yl)phenyl)imino)methyl)benzoate (122)**

Yellow solid, 92.21 mg, 90% yield; ^1^H NMR (400 MHz, CDCl_3_) *δ* 8.96 (s, 2H), 8.72 (d, *J* = 4.9 Hz, 1H), 8.21 (dd, *J* = 8.1, 1.7 Hz, 1H), 7.98–7.93 (m, 2H), 7.85 (d, *J* = 8.0 Hz, 1H), 7.79 (dd, *J* = 3.6, 1.3 Hz, 2H), 7.55 (t, *J* = 7.7 Hz, 1H), 7.41–7.37 (m, 1H), 7.28 (d, *J* = 9.7 Hz, 1H), 4.00 (s, 3H); ^13^C NMR (101 MHz, CDCl_3_) *δ* 165.12, 156.57, 154.69, 150.78, 149.65, 140.57, 138.61, 136.91, 134.31, 133.40, 131.55, 129.72, 128.93, 125.85, 122.47, 121.68, 120.68, 119.69, 116.64, 116.27, 52.82; HRMS-ESI (*m/z*) [M + H]^+^ calculated for C_21_H_16_N_3_O_2_ 342.11978, found 342.12360.

**Methyl (E)-4-cyano-3-(((4-(pyridin-2-ylmethyl)phenyl)imino)methyl)benzoate (123)**

Orange solid, 97.23 mg, 91% yield; ^1^H NMR (400 MHz, CDCl_3_) *δ* 8.93 (d, *J* = 1.7 Hz, 1H), 8.87 (s, 1H), 8.61–8.55 (m, 1H), 8.19 (dd, *J* = 8.1, 1.7 Hz, 1H), 7.82 (d, *J* = 8.1 Hz, 1H), 7.62 (td, *J* = 7.7, 1.8 Hz, 1H), 7.34 (d, *J* = 8.4 Hz, 2H), 7.28 (d, *J* = 8.4 Hz, 2H), 7.15 (ddd, *J* = 7.4, 2.8, 1.0 Hz, 2H), 4.21 (s, 2H), 3.99 (s, 3H); ^13^C NMR (101 MHz, CDCl_3_) *δ* 165.19, 160.57, 153.94, 149.30, 148.67, 139.03, 138.78, 136.77, 134.32, 133.31, 131.41, 130.07, 128.75, 123.18, 121.51, 121.43, 116.66, 116.28, 52.83, 44.13; HRMS-ESI (*m/z*) [M + H]^+^ calculated for C_22_H_18_N_3_O_2_ 356.13543, found 356.13895.

**Methyl (E)-4-cyano-3-(((4-(pyridin-3-yloxy)phenyl)imino)methyl)benzoate (124)**

Yellow oil, 98.10 mg, 92% yield; ^1^H NMR (400 MHz, CDCl_3_) *δ* 8.94 (d, *J* = 1.7 Hz, 1H), 8.89 (s, 1H), 8.45 (d, *J* = 2.7 Hz, 1H), 8.40 (dd, *J* = 4.5, 1.5 Hz, 1H), 8.20 (dd, *J* = 8.1, 1.7 Hz, 1H), 7.84 (d, *J* = 8.1 Hz, 1H), 7.39–7.36 (m, 2H), 7.36–7.34 (m, 1H), 7.33–7.29 (m, 1H), 7.12–7.08 (m, 2H), 4.00 (s, 3H); ^13^C NMR (101 MHz, CDCl_3_) *δ* 165.15, 155.84, 153.77, 153.73, 146.22, 144.42, 141.24, 138.61, 134.34, 133.38, 131.50, 128.75, 125.76, 124.25, 123.07, 119.66, 116.62, 116.29, 52.87; HRMS-ESI (*m/z*) [M + H]^+^ calculated for C_21_H_16_N_3_O_3_ 358.11470, found 358.11850.

**Methyl (E)-4-cyano-3-(((4-((5-(trifluoromethyl)pyridin-2-yl)oxy)phenyl)imino)methyl)benzoate (125)**

Yellow solid, 117.51 mg, 92% yield; ^1^H NMR (400 MHz, CDCl_3_) *δ* 8.94 (d, *J* = 1.8 Hz, 1H), 8.90 (s, 1H), 8.48–8.45 (m, 1H), 8.21 (dd, *J* = 8.1, 1.7 Hz, 1H), 7.93 (dd, *J* = 8.7, 2.5 Hz, 1H), 7.84 (d, *J* = 8.1 Hz, 1H), 7.42 (d, *J* = 8.7 Hz, 2H), 7.23 (d, *J* = 8.7 Hz, 2H), 7.06 (d, *J* = 8.7 Hz, 1H), 4.00 (s, 3H); ^13^C NMR (101 MHz, CDCl_3_) *δ* 165.62, 165.14, 154.28, 152.47, 147.43, 145.43 (d, *J* = 4.4 Hz), 138.58, 136.81 (d, *J* = 3.8 Hz), 134.33, 133.37, 131.54, 128.80, 122.69, 122.34, 122.27, 121.73 (d, *J* = 33.4 Hz), 116.70, 116.24, 111.48, 52.85; ^19^F NMR (376 MHz, CDCl_3_) *δ* -61.67; HRMS-ESI (*m/z*) [M + H]^+^ calculated for C_22_H_15_F_3_N_3_O_3_ 426.10208, found 426.10663.

**Methyl (E)-4-cyano-3-(((4-((2-oxopyridin-1(2H)-yl)methyl)phenyl)imino)methyl)benzoate (126)**

Yellow solid, 103.56 mg, 93% yield; ^1^H NMR (400 MHz, CDCl_3_) *δ* 8.92 (d, *J* = 1.7 Hz, 1H), 8.84 (s, 1H), 8.21 (dd, *J* = 8.0, 1.7 Hz, 1H), 7.83 (d, *J* = 8.1 Hz, 1H), 7.37 (td, *J* = 5.6, 1.9 Hz, 2H), 7.35–7.31 (m, 1H), 7.31–7.27 (m, 3H), 6.64 (dt, *J* = 9.0, 1.2 Hz, 1H), 6.18 (td, *J* = 6.7, 1.4 Hz, 1H), 5.18 (s, 2H), 3.99 (s, 3H); ^13^C NMR (101 MHz, CDCl_3_) *δ* 165.06, 162.63, 154.74, 150.05, 139.56, 138.43, 137.13, 135.54, 134.27, 133.33, 131.58, 129.14, 128.78, 121.59, 121.20, 116.67, 116.16, 106.43, 52.82, 51.56; HRMS-ESI (*m/z*) [M + H]^+^ calculated for C_22_H_18_N_3_O_3_ 372.13035, found 372.13391.

**Methyl (E)-4-cyano-3-(((2,3,5,6,8,9,11,12-octahydrobenzo[b][**1,4,7,10,13**]pentaoxacyclopentadecin-15-yl)imino)methyl)benzoate (127)**

Yellow solid, 126.11 mg, 92% yield; ^1^H NMR (400 MHz, CDCl_3_) *δ* 8.90 (d, *J* = 1.6 Hz, 1H), 8.85 (s, 1H), 8.15 (dd, *J* = 8.1, 1.7 Hz, 1H), 7.80 (d, *J* = 8.0 Hz, 1H), 6.99–6.93 (m, 2H), 6.90 (d, *J* = 9.1 Hz, 1H), 4.22–4.15 (m, 4H), 3.98 (d, *J* = 1.1 Hz, 3H), 3.93 (dq, *J* = 4.4, 2.4 Hz, 4H), 3.80–3.73 (m, 8H); ^13^C NMR (101 MHz, CDCl_3_) *δ* 165.19, 151.88, 149.53, 149.12, 143.59, 138.94, 134.24, 133.29, 131.05, 128.55, 116.38, 116.30, 113.98, 113.89, 107.94, 71.00, 70.39, 70.36, 69.45, 69.40, 69.07, 68.90, 52.76; HRMS-ESI (*m/z*) [M + H]^+^ calculated for C_24_H_27_N_2_O_7_ 455.17736, found 455.18143.

**Methyl (E)-4-cyano-3-(((2,2-difluorobenzo[d][**1,3**]dioxol-5-yl)imino)methyl)benzoate (128)**

Yellow solid, 94.12 mg, 91% yield; ^1^H NMR (400 MHz, CDCl_3_) *δ* 8.90 (d, *J* = 1.7 Hz, 1H), 8.83 (s, 1H), 8.21 (dd, *J* = 8.1, 1.7 Hz, 1H), 7.84 (d, *J* = 8.1 Hz, 1H), 7.11 (td, *J* = 5.0, 2.4 Hz, 3H), 4.00 (s, 3H); ^13^C NMR (101 MHz, CDCl_3_) *δ* 165.07, 154.38, 146.39, 144.42, 143.06, 138.20, 134.39, 133.46, 131.78, 131.75, 128.91, 117.12, 116.71, 116.17, 109.75, 103.08, 52.89; ^19^F NMR (376 MHz, CDCl_3_) *δ* -49.67; HRMS-ESI (*m/z*) [M + H]^+^ calculated for C_17_H_11_F_2_N_2_O_4_ 345.06422, found 345.06753.

**Methyl (E)-4-cyano-3-(((2,3-dihydrobenzofuran-7-yl)imino)methyl)benzoate (129)**

Yellow solid, 83.86 mg, 91% yield; ^1^H NMR (400 MHz, CDCl_3_) *δ* 8.86 (d, *J* = 2.1 Hz, 2H), 8.22 (dd, *J* = 8.1, 1.7 Hz, 1H), 7.85 (d, *J* = 8.1 Hz, 1H), 7.36 (t, *J* = 7.6 Hz, 1H), 7.19 (d, *J* = 7.5 Hz, 1H), 7.09 (d, *J* = 7.8 Hz, 1H), 5.31–5.27 (m, 2H), 5.19 (d, *J* = 2.2 Hz, 2H), 4.01 (s, 3H); ^13^C NMR (101 MHz, CDCl_3_) *δ* 165.09, 154.83, 144.44, 140.76, 138.37, 134.30, 133.56, 131.67, 129.10, 128.84, 119.88, 116.56, 116.27, 73.82, 72.71, 52.90; HRMS-ESI (*m/z*) [M + H]^+^ calculated for C_18_H_15_N_2_O_3_ 307.10380, found 307.10773.

**Methyl (E)-4-cyano-3-(((1,3-dihydroisobenzofuran-5-yl)imino)methyl)benzoate (130)**

Orange solid, 85.84 mg, 93% yield; ^1^H NMR (400 MHz, CDCl_3_) *δ* 8.94 (d, *J* = 1.7 Hz, 1H), 8.89 (s, 1H), 8.21 (dd, *J* = 8.1, 1.7 Hz, 1H), 7.84 (d, *J* = 8.1 Hz, 1H), 7.30 (d, *J* = 8.0 Hz, 1H), 7.24 (dd, *J* = 8.0, 1.9 Hz, 1H), 7.20 (d, *J* = 1.9 Hz, 1H), 5.15 (s, 4H), 4.00 (s, 3H); ^13^C NMR (101 MHz, CDCl_3_) *δ* 165.13, 154.23, 149.88, 140.65, 138.59, 138.48, 134.33, 133.35, 131.52, 128.78, 121.71, 120.84, 116.65, 116.26, 113.62, 73.34, 73.30, 52.85; HRMS-ESI (*m/z*) [M + H]^+^ calculated for C_18_H_15_N_2_O_3_ 307.10380, found 307.10654.

**Methyl (E)-3-((benzofuran-5-ylimino)methyl)-4-cyanobenzoate (131)**

Orange solid, 83.86 mg, 92% yield; ^1^H NMR (400 MHz, CDCl_3_) *δ* 8.96 (d, *J* = 1.7 Hz, 1H), 8.94 (s, 1H), 8.18 (dd, *J* = 8.0, 1.7 Hz, 1H), 7.82 (d, *J* = 8.1 Hz, 1H), 7.67 (d, *J* = 2.2 Hz, 1H), 7.58 (d, *J* = 2.2 Hz, 1H), 7.54 (d, *J* = 8.6 Hz, 1H), 7.35 (dd, *J* = 8.7, 2.2 Hz, 1H), 6.82 (d, *J* = 2.2 Hz, 1H), 4.00 (s, 3H); ^13^C NMR (101 MHz, CDCl_3_) *δ* 165.21, 154.44, 153.24, 146.17, 145.73, 138.89, 134.31, 133.30, 131.23, 128.70, 128.28, 118.68, 116.53, 116.36, 113.37, 111.94, 106.99, 52.80; HRMS-ESI (*m/z*) [M + H]^+^ calculated for C_18_H_13_N_2_O_3_ 305.08815, found 305.09138.

**Ethyl (E)-5-((2-cyano-5-(methoxycarbonyl)benzylidene)amino)benzofuran-2-carboxylate (132)**

Yellow solid, 104.55 mg, 93% yield; ^1^H NMR (400 MHz, CDCl_3_) *δ* 8.95 (d, *J* = 1.7 Hz, 1H), 8.92 (s, 1H), 8.21 (dd, *J* = 8.1, 1.7 Hz, 1H), 7.84 (d, *J* = 8.1 Hz, 1H), 7.66–7.60 (m, 2H), 7.56 (s, 1H), 7.48 (dd, *J* = 8.8, 2.2 Hz, 1H), 4.46 (q, *J* = 7.2 Hz, 2H), 4.00 (s, 3H), 1.44 (t, *J* = 7.1 Hz, 3H); ^13^C NMR (101 MHz, CDCl_3_) *δ* 165.12, 159.31, 154.95, 154.16, 146.82, 146.60, 138.54, 134.33, 133.38, 131.52, 128.79, 127.76, 122.09, 116.62, 116.29, 114.47, 113.92, 113.03, 61.66, 52.87, 14.30; HRMS-ESI (*m/z*) [M + H]^+^ calculated for C_21_H_17_N_2_O_5_ 377.10928, found 377.11343.

**Methyl (E)-4-cyano-3-((dibenzo[b,d]furan-2-ylimino)methyl)benzoate (133)**

Yellow solid, 100.20 mg, 94% yield; ^1^H NMR (400 MHz, CDCl_3_) *δ* 9.00 (s, 1H), 8.98 (d, *J* = 1.7 Hz, 1H), 8.20 (dd, *J* = 8.0, 1.7 Hz, 1H), 8.00 (d, *J* = 7.6 Hz, 1H), 7.95 (d, *J* = 2.2 Hz, 1H), 7.85 (d, *J* = 8.0 Hz, 1H), 7.61 (dd, *J* = 11.7, 8.4 Hz, 2H), 7.51 (dt, *J* = 8.7, 2.0 Hz, 2H), 7.38 (t, *J* = 7.5 Hz, 1H), 4.01 (s, 3H); ^13^C NMR (101 MHz, CDCl_3_) *δ* 165.21, 156.90, 155.63, 153.31, 145.45, 138.80, 134.32, 133.39, 131.34, 128.76, 127.65, 125.15, 124.01, 122.96, 121.29, 120.93, 116.50, 116.43, 113.15, 112.25, 111.84, 52.86; HRMS-ESI (*m/z*) [M + H]^+^ calculated for C_22_H_15_N_2_O_3_ 355.10380, found 355.10773.

**Methyl (E)-4-cyano-3-(((4-(tetrahydro-2H-pyran-4-yl)phenyl)imino)methyl)benzoate (134)**

Red-brown solid, 95.16 mg, 91% yield; ^1^H NMR (400 MHz, CDCl_3_) *δ* 8.95 (d, *J* = 1.7 Hz, 1H), 8.89 (s, 1H), 8.20 (dd, *J* = 8.1, 1.7 Hz, 1H), 7.83 (d, *J* = 8.1 Hz, 1H), 7.31 (s, 4H), 4.14–4.08 (m, 2H), 4.00 (s, 3H), 3.55 (td, *J* = 11.4, 3.0 Hz, 2H), 2.87–2.77 (m, 1H), 1.87–1.78 (m, 4H); ^13^C NMR (101 MHz, CDCl_3_) *δ* 165.17, 153.79, 148.48, 145.46, 138.78, 134.29, 133.30, 131.37, 128.70, 127.64, 121.43, 116.63, 116.28, 68.29, 52.82, 41.18, 33.86; HRMS-ESI (*m/z*) [M + H]^+^ calculated for C_21_H_21_N_2_O_3_ 349.15075, found 349.15432.

**Methyl (E)-4-cyano-3-(((4-(furan-2-yl)phenyl)imino)methyl)benzoate (135)**

Dark brown solid, 91.24 mg, 92% yield; ^1^H NMR (400 MHz, CDCl_3_) *δ* 8.96 (d, *J* = 1.7 Hz, 1H), 8.92 (s, 1H), 8.20 (dd, *J* = 8.1, 1.7 Hz, 1H), 7.84 (d, *J* = 8.1 Hz, 1H), 7.76–7.72 (m, 2H), 7.50 (d, *J* = 1.8 Hz, 1H), 7.38 (d, *J* = 8.5 Hz, 2H), 6.70 (d, *J* = 3.5 Hz, 1H), 6.50 (dd, *J* = 3.4, 1.8 Hz, 1H), 4.00 (s, 3H); ^13^C NMR (101 MHz, CDCl_3_) *δ* 165.18, 153.65, 153.36, 148.89, 142.38, 138.71, 134.31, 133.36, 131.45, 130.25, 128.77, 124.70, 121.88, 116.62, 116.31, 111.85, 105.52, 52.86; HRMS-ESI (*m/z*) [M + H]^+^ calculated for C_20_H_15_N_2_O_3_ 331.10380, found 331.10768.

**Methyl (E)-3-(((3-benzyl-2-oxo-2,3-dihydrobenzo[d]oxazol-6-yl)imino)methyl)-4-cyanobenzoate (136)**

Yellow solid, 113.60 mg, 92% yield; ^1^H NMR (400 MHz, CDCl_3_) *δ* 8.91 (d, *J* = 1.7 Hz, 1H), 8.85 (s, 1H), 8.20 (dd, *J* = 8.0, 1.7 Hz, 1H), 7.84 (d, *J* = 8.1 Hz, 1H), 7.38 (d, *J* = 4.6 Hz, 3H), 7.36–7.31 (m, 2H), 7.29 (d, *J* = 1.9 Hz, 1H), 7.15 (dd, *J* = 8.3, 2.0 Hz, 1H), 6.88 (d, *J* = 8.3 Hz, 1H), 5.05 (s, 2H), 4.00 (s, 3H); ^13^C NMR (101 MHz, CDCl_3_) *δ* 165.12, 154.75, 153.53, 145.50, 143.24, 138.44, 134.38, 134.35, 133.41, 131.54, 130.38, 129.06, 128.81, 128.43, 127.65, 117.81, 116.59, 116.26, 109.14, 103.66, 52.89, 46.29; HRMS-ESI (*m/z*) [M + H]^+^ calculated for C_24_H_18_N_3_O_4_ 412.12526, found 412.12884.

**Methyl (E)-3-((benzo[d]oxazol-6-ylimino)methyl)-4-cyanobenzoate (137)**

Yellow solid, 82.66 mg, 90% yield; ^1^H NMR (400 MHz, CDCl_3_) *δ* 8.96 (d, *J* = 1.7 Hz, 1H), 8.92 (s, 1H), 8.22 (dd, *J* = 8.0, 1.7 Hz, 1H), 8.14 (s, 1H), 7.84 (dd, *J* = 12.2, 8.2 Hz, 2H), 7.56 (d, *J* = 2.0 Hz, 1H), 7.40 (dd, *J* = 8.5, 2.0 Hz, 1H), 4.00 (s, 3H); ^13^C NMR (101 MHz, CDCl_3_) *δ* 165.09, 154.93, 153.36, 150.56, 148.43, 139.43, 138.35, 134.38, 133.44, 131.72, 128.90, 120.86, 118.95, 116.76, 116.22, 103.76, 52.90; HRMS-ESI (*m/z*) [M + H]^+^ calculated for C_17_H_12_N_3_O_3_ 306.08340, found 306.08734.

**Methyl (E)-4-cyano-3-(((3-methyl-2-oxo-2,3-dihydrobenzo[d]oxazol-6-yl)imino)methyl)benzoate (138)**

Yellow solid, 92.33 mg, 92% yield; ^1^H NMR (400 MHz, CDCl_3_) *δ* 8.94 (d, *J* = 1.7 Hz, 1H), 8.89 (s, 1H), 8.21 (dd, *J* = 8.0, 1.7 Hz, 1H), 7.85 (dd, *J* = 8.1, 0.6 Hz, 1H), 7.31–7.28 (m, 1H), 7.26 (d, *J* = 2.0 Hz, 1H), 7.02 (d, *J* = 8.2 Hz, 1H), 4.01 (s, 3H), 3.46 (s, 3H); ^13^C NMR (101 MHz, CDCl_3_) *δ* 165.12, 154.73, 153.39, 145.38, 143.20, 138.48, 134.37, 133.44, 131.54, 131.35, 128.83, 118.00, 116.57, 116.30, 108.23, 103.52, 52.89, 28.30; HRMS-ESI (*m/z*) [M + H]^+^ calculated for C_18_H_14_N_3_O_4_ 336.09396, found 336.09772.

**Methyl (E)-4-cyano-3-(((2-methylbenzo[d]oxazol-5-yl)imino)methyl)benzoate (139)**

Red-brown solid, 86.66 mg, 90% yield; ^1^H NMR (400 MHz, CDCl_3_) *δ* 8.95 (d, *J* = 1.7 Hz, 1H), 8.91 (s, 1H), 8.20 (dd, *J* = 8.0, 1.7 Hz, 1H), 7.84 (d, *J* = 8.1 Hz, 1H), 7.61 (d, *J* = 2.0 Hz, 1H), 7.50 (d, *J* = 8.6 Hz, 1H), 7.32 (dd, *J* = 8.6, 2.1 Hz, 1H), 3.99 (s, 3H), 2.66 (s, 3H); ^13^C NMR (101 MHz, CDCl_3_) *δ* 165.20, 165.15, 154.20, 150.36, 147.12, 142.35, 138.57, 134.30, 133.36, 131.48, 128.78, 118.68, 116.66, 116.26, 111.69, 110.56, 52.85, 14.62; HRMS-ESI (*m/z*) [M + H]^+^ calculated for C_18_H_14_N_3_O_3_ 320.09905, found 320.10263.

**Methyl (E)-4-cyano-3-(((4-methyl-3-oxo-3,4-dihydro-2H-benzo[b][1,4]oxazin-6-yl)imino)methyl)benzoate (140)**

Orange solid, 95.96 mg, 92% yield; ^1^H NMR (400 MHz, CDCl_3_) *δ* 8.92 (d, *J* = 1.7 Hz, 1H), 8.87 (s, 1H), 8.20 (dd, *J* = 8.0, 1.7 Hz, 1H), 7.84 (d, *J* = 8.1 Hz, 1H), 7.03 (s, 3H), 4.66 (s, 2H), 3.99 (s, 3H), 3.41 (s, 3H); ^13^C NMR (101 MHz, CDCl_3_) *δ* 165.10, 164.29, 153.31, 145.11, 144.64, 138.52, 134.32, 133.41, 131.47, 130.10, 128.71, 117.37, 116.52, 116.28, 115.30, 109.72, 67.54, 52.87, 28.13; HRMS-ESI (*m/z*) [M + H]^+^ calculated for C_19_H_16_N_3_O_4_ 350.10961, found 350.11389.

**Methyl (E)-4-cyano-3-(((3-(oxazol-5-yl)phenyl)imino)methyl)benzoate (141)**

Yellow solid, 90.20 mg, 91% yield; ^1^H NMR (400 MHz, CDCl_3_) *δ* 8.96 (d, *J* = 1.7 Hz, 1H), 8.91 (s, 1H), 8.24 (dd, *J* = 8.1, 1.7 Hz, 1H), 7.97 (s, 1H), 7.87 (d, *J* = 8.1 Hz, 1H), 7.64–7.59 (m, 2H), 7.54–7.48 (m, 1H), 7.44 (s, 1H), 7.31–7.27 (m, 1H), 4.01 (s, 3H); ^13^C NMR (101 MHz, CDCl_3_) *δ* 165.07, 155.28, 150.99, 150.92, 150.67, 138.35, 134.39, 133.45, 131.79, 130.03, 128.94, 128.88, 123.17, 122.12, 120.86, 117.38, 116.79, 116.20, 52.89; HRMS-ESI (*m/z*) [M + H]^+^ calculated for C_19_H_14_N_3_O_3_ 332.09905, found 332.10260.

**Methyl (E)-4-cyano-3-(((3-(isoxazol-5-yl)phenyl)imino)methyl)benzoate (142)**

Yellow solid, 90.75 mg, 91% yield; ^1^H NMR (400 MHz, CDCl_3_) *δ* 8.96 (d, *J* = 1.7 Hz, 1H), 8.92 (s, 1H), 8.33 (d, *J* = 1.8 Hz, 1H), 8.25 (dd, *J* = 8.1, 1.7 Hz, 1H), 7.87 (dd, *J* = 8.1, 0.5 Hz, 1H), 7.78–7.74 (m, 1H), 7.72 (t, *J* = 1.9 Hz, 1H), 7.56 (t, *J* = 7.8 Hz, 1H), 7.38 (ddd, *J* = 7.9, 2.2, 1.1 Hz, 1H), 6.60 (d, *J* = 1.9 Hz, 1H), 4.01 (s, 3H); ^13^C NMR (101 MHz, CDCl_3_) *δ* 168.69, 165.07, 155.58, 151.09, 150.86, 138.31, 134.47, 133.49, 131.89, 130.15, 129.05, 128.44, 124.63, 122.36, 118.92, 116.86, 116.18, 99.27, 52.89; HRMS-ESI (*m/z*) [M + H]^+^ calculated for C_19_H_14_N_3_O_3_ 332.09905, found 332.10284.

**Methyl (E)-4-cyano-3-(((3-morpholinophenyl)imino)methyl)benzoate (143)**

Red solid, 95.43 mg, 91% yield; ^1^H NMR (400 MHz, CDCl_3_) *δ* 8.93 (d, *J* = 1.6 Hz, 1H), 8.87 (s, 1H), 8.20 (dd, *J* = 8.1, 1.7 Hz, 1H), 7.83 (d, *J* = 8.0 Hz, 1H), 7.33 (t, *J* = 7.9 Hz, 1H), 6.89–6.79 (m, 3H), 3.99 (s, 3H), 3.90–3.86 (m, 4H), 3.24–3.20 (m, 4H); ^13^C NMR (101 MHz, CDCl_3_) *δ* 165.15, 154.33, 152.18, 151.44, 138.70, 134.31, 133.35, 131.47, 129.96, 128.77, 116.67, 116.27, 114.56, 111.55, 109.11, 66.81, 52.83, 49.08; HRMS-ESI (*m/z*) [M + H]^+^ calculated for C_20_H_20_N_3_O_3_ 350.14600, found 350.14981.

**Methyl (E)-4-cyano-3-(((4-morpholinophenyl)imino)methyl)benzoate (144)**

Dark brown solid, 96.27 mg, 92% yield; ^1^H NMR (400 MHz, CDCl_3_) *δ* 8.93 (d, *J* = 1.7 Hz, 1H), 8.91 (s, 1H), 8.14 (dd, *J* = 8.1, 1.7 Hz, 1H), 7.80 (d, *J* = 8.1 Hz, 1H), 7.39 (d, *J* = 8.9 Hz, 2H), 6.96 (d, *J* = 8.9 Hz, 2H), 3.99 (s, 3H), 3.88 (t, *J* = 4.8 Hz, 4H), 3.23 (t, *J* = 4.8 Hz, 4H); ^13^C NMR (101 MHz, CDCl_3_) *δ* 165.32, 151.09, 150.60, 141.76, 139.32, 134.22, 133.24, 130.79, 128.42, 122.91, 116.50, 116.19, 115.66, 66.75, 52.78, 48.85; HRMS-ESI (*m/z*) [M + H]^+^ calculated for C_20_H_20_N_3_O_3_ 350.14600, found 350.15005.

**Methyl (E)-3-((benzo[b]thiophen-5-ylimino)methyl)-4-cyanobenzoate (145)**

Orange solid, 90.03 mg, 94% yield; ^1^H NMR (400 MHz, CDCl_3_) *δ* 9.00 (d, *J* = 2.5 Hz, 2H), 8.21 (dd, *J* = 8.1, 1.7 Hz, 1H), 7.93 (d, *J* = 8.6 Hz, 1H), 7.85 (d, *J* = 8.0 Hz, 1H), 7.79 (d, *J* = 2.0 Hz, 1H), 7.52 (d, *J* = 5.4 Hz, 1H), 7.42–7.38 (m, 2H), 4.01 (s, 3H); ^13^C NMR (101 MHz, CDCl_3_) *δ* 165.22, 153.88, 147.12, 140.48, 138.93, 138.81, 134.34, 133.37, 131.41, 128.78, 127.93, 124.16, 123.17, 118.42, 116.61, 116.38, 115.84, 52.87; HRMS-ESI (*m/z*) [M + H]^+^ calculated for C_18_H_13_N_2_O_2_S 321.06530, found 321.06882.

**Methyl (E)-4-cyano-3-(((4-(thiophen-3-yl)phenyl)imino)methyl)benzoate (146)**

Red-brown solid, 94.99 mg, 91% yield; ^1^H NMR (400 MHz, CDCl_3_) *δ* 8.97 (d, *J* = 1.7 Hz, 1H), 8.94 (s, 1H), 8.21 (dd, *J* = 8.2, 1.7 Hz, 1H), 7.85 (d, *J* = 8.2 Hz, 1H), 7.68 (d, *J* = 8.6 Hz, 2H), 7.51 (dd, *J* = 2.8, 1.6 Hz, 1H), 7.44–7.37 (m, 4H), 4.00 (s, 3H); ^13^C NMR (101 MHz, CDCl_3_) *δ* 165.20, 153.81, 148.95, 141.47, 138.74, 135.23, 134.33, 133.36, 131.47, 128.78, 127.30, 126.45, 126.16, 121.90, 120.53, 116.66, 116.32, 52.87; HRMS-ESI (*m/z*) [M + H]^+^ calculated for C_20_H_15_N_2_O_2_S 347.08095, found 347.08430.

**Methyl (E)-4-cyano-3-(((2-methylbenzo[d]thiazol-5-yl)imino)methyl)benzoate (147)**

Yellow solid, 93.35 mg, 93% yield; ^1^H NMR (400 MHz, CDCl_3_) *δ* 8.97 (d, *J* = 1.7 Hz, 1H), 8.95 (s, 1H), 8.20 (dd, *J* = 8.0, 1.7 Hz, 1H), 7.88 (d, *J* = 2.0 Hz, 1H), 7.84 (dd, *J* = 8.3, 4.1 Hz, 2H), 7.37 (dd, *J* = 8.4, 2.1 Hz, 1H), 4.00 (s, 3H), 2.85 (s, 3H); ^13^C NMR (101 MHz, CDCl_3_) *δ* 168.62, 165.13, 154.72, 154.23, 148.83, 138.51, 134.63, 134.29, 133.34, 131.56, 128.80, 121.85, 118.90, 116.77, 116.19, 114.38, 52.85, 20.25; HRMS-ESI (*m/z*) [M + H]^+^ calculated for C_18_H_14_N_3_O_2_S 336.07620, found 336.08017.

**Methyl (E)-4-cyano-3-(((2-morpholinobenzo[d]thiazol-5-yl)imino)methyl)benzoate (148)**

Red-brown solid, 115.02 mg, 94% yield; ^1^H NMR (400 MHz, CDCl_3_) *δ* 8.97 (d, *J* = 1.7 Hz, 1H), 8.94 (s, 1H), 8.19 (dd, *J* = 8.0, 1.7 Hz, 1H), 7.83 (d, *J* = 8.1 Hz, 1H), 7.64 (d, *J* = 8.3 Hz, 1H), 7.54 (d, *J* = 2.0 Hz, 1H), 7.15 (dd, *J* = 8.3, 2.1 Hz, 1H), 4.00 (s, 3H), 3.87–3.83 (m, 4H), 3.68–3.63 (m, 4H); ^13^C NMR (101 MHz, CDCl_3_) *δ* 169.85, 165.19, 153.86, 153.42, 148.95, 138.74, 134.23, 133.29, 131.34, 129.80, 128.72, 121.15, 116.67, 116.37, 116.26, 110.85, 66.17, 52.81, 48.42; HRMS-ESI (*m/z*) [M + H]^+^ calculated for C_21_H_19_N_4_O_3_S 407.11332, found 407.11758.

**Methyl (E)-3-(((2-chlorobenzo[d]thiazol-6-yl)imino)methyl)-4-cyanobenzoate (149)**

Yellow solid, 100.59 mg, 94% yield; ^1^H NMR (400 MHz, CDCl_3_) *δ* 8.94 (d, *J* = 1.7 Hz, 1H), 8.91 (s, 1H), 8.22 (dd, *J* = 8.1, 1.7 Hz, 1H), 7.98 (d, *J* = 8.7 Hz, 1H), 7.85 (d, *J* = 8.0 Hz, 1H), 7.72 (d, *J* = 2.0 Hz, 1H), 7.48 (dd, *J* = 8.7, 2.1 Hz, 1H), 4.00 (s, 3H); ^13^C NMR (101 MHz, CDCl_3_) *δ* 165.04, 155.11, 153.50, 150.25, 148.00, 138.24, 137.16, 134.39, 133.46, 131.81, 128.95, 123.47, 120.86, 116.74, 116.20, 113.52, 52.91; HRMS-ESI (*m/z*) [M + H]^+^ calculated for C_17_H_11_ClN_3_O_2_S 356.02158, found 356.02506.

**Methyl (E)-3-((benzo[d]isothiazol-5-ylimino)methyl)-4-cyanobenzoate (150)**

Yellow solid, 90.35 mg, 94% yield; ^1^H NMR (400 MHz, CDCl_3_) *δ* 8.99 (s, 2H), 8.97 (d, *J* = 0.9 Hz, 1H), 8.24 (dd, *J* = 8.1, 1.7 Hz, 1H), 8.02 (dd, *J* = 8.6, 0.9 Hz, 1H), 7.97 (dd, *J* = 2.0, 0.6 Hz, 1H), 7.87 (d, *J* = 8.1 Hz, 1H), 7.58 (dd, *J* = 8.6, 1.9 Hz, 1H), 4.01 (s, 3H); ^13^C NMR (101 MHz, CDCl_3_) *δ* 165.09, 155.13, 155.10, 150.64, 147.85, 138.37, 137.06, 134.43, 133.47, 131.78, 128.95, 122.43, 120.37, 116.77, 116.25, 115.55, 52.92; HRMS-ESI (*m/z*) [M + H]^+^ calculated for C_17_H_12_N_3_O_2_S 322.06055, found 322.06348.

**Methyl (E)-4-cyano-3-(((3-(thiazol-4-yl)phenyl)imino)methyl)benzoate (151)**

Orange solid, 95.62 mg, 92% yield; ^1^H NMR (400 MHz, CDCl_3_) *δ* 8.96 (d, *J* = 1.7 Hz, 1H), 8.94 (s, 1H), 8.90 (d, *J* = 2.0 Hz, 1H), 8.21 (dd, *J* = 8.1, 1.7 Hz, 1H), 7.88 (dt, *J* = 3.8, 1.8 Hz, 2H), 7.84 (d, *J* = 8.1 Hz, 1H), 7.62 (d, *J* = 2.0 Hz, 1H), 7.51 (t, *J* = 8.0 Hz, 1H), 7.31 (ddd, *J* = 7.9, 2.1, 1.1 Hz, 1H), 4.00 (s, 3H); ^13^C NMR (101 MHz, CDCl_3_) *δ* 165.13, 155.63, 154.76, 152.98, 150.84, 138.60, 135.40, 134.35, 133.41, 131.59, 129.81, 128.95, 125.39, 120.83, 119.33, 116.68, 116.27, 113.29, 52.84; HRMS-ESI (*m/z*) [M + H]^+^ calculated for C_19_H_14_N_3_O_2_S 348.07620, found 348.08066.

**Methyl (E)-4-cyano-3-(((4-(thiazol-2-yloxy)phenyl)imino)methyl)benzoate (152)**

Yellow solid, 100.45 mg, 92% yield; ^1^H NMR (400 MHz, CDCl_3_) *δ* 8.93 (d, *J* = 1.7 Hz, 1H), 8.88 (s, 1H), 8.21 (dd, *J* = 8.1, 1.7 Hz, 1H), 7.85 (d, *J* = 8.1 Hz, 1H), 7.42–7.35 (m, 4H), 7.28–7.25 (m, 1H), 6.86 (d, *J* = 3.8 Hz, 1H), 4.00 (s, 3H); ^13^C NMR (101 MHz, CDCl_3_) *δ* 173.50, 165.06, 154.52, 154.43, 147.65, 138.42, 137.51, 134.27, 133.34, 131.55, 128.76, 122.70, 120.94, 116.65, 116.17, 113.16, 52.82; HRMS-ESI (*m/z*) [M + H]^+^ calculated for C_19_H_14_N_3_O_3_S 364.07112, found 364.07477.

**Methyl (E)-4-cyano-3-((2-(o-tolyl)hydrazineylidene)methyl)benzoate (153)**

Dark brown solid, 80.99 mg, 92% yield; ^1^H NMR (400 MHz, CDCl_3_) *δ* 8.69 (d, *J* = 1.6 Hz, 1H), 8.10 (d, *J* = 4.9 Hz, 2H), 7.95 (dd, *J* = 8.1, 1.7 Hz, 1H), 7.70 (d, *J* = 8.1 Hz, 1H), 7.64 (d, *J* = 8.1 Hz, 1H), 7.29–7.23 (m, 1H), 7.12 (d, *J* = 7.4 Hz, 1H), 6.90 (td, *J* = 7.3, 1.2 Hz, 1H), 4.00 (s, 3H), 2.30 (s, 3H); ^13^C NMR (101 MHz, CDCl_3_) *δ* 165.57, 141.29, 138.93, 134.01, 133.04, 131.90, 130.57, 128.08, 127.42, 126.63, 121.08, 120.82, 117.13, 113.38, 112.71, 52.74, 17.02; HRMS-ESI (*m/z*) [M + H]^+^ calculated for C_17_H_16_N_3_O_2_ 294.11978, found 294.12307.

**Methyl (E)-3-((2-(4-(tert-butyl)phenyl)hydrazineylidene)methyl)-4-cyanobenzoate (154)**

Yellow solid, 92.36 mg, 92% yield; ^1^H NMR (400 MHz, CDCl_3_) *δ* 8.63 (d, *J* = 1.6 Hz, 1H), 8.19 (s, 1H), 7.97 (s, 1H), 7.93 (dd, *J* = 8.1, 1.7 Hz, 1H), 7.69 (d, *J* = 8.1 Hz, 1H), 7.35 (d, *J* = 8.7 Hz, 2H), 7.14 (d, *J* = 8.7 Hz, 2H), 3.99 (s, 3H), 1.32 (s, 9H); ^13^C NMR (101 MHz, CDCl_3_) *δ* 165.64, 144.37, 141.04, 139.08, 133.94, 133.13, 130.52, 127.82, 126.66, 126.23, 117.20, 113.01, 112.46, 52.73, 34.19, 31.46; HRMS-ESI (*m/z*) [M + H]^+^ calculated for C_20_H_22_N_3_O_2_ 336.16673, found 336.17081.

**Methyl (E)-3-((2-(4-(benzyloxy)phenyl)hydrazineylidene)methyl)-4-cyanobenzoate (155)**

Red-brown solid, 105.37 mg, 91% yield; ^1^H NMR (400 MHz, CDCl_3_) *δ* 8.61–8.58 (m, 1H), 8.19 (s, 1H), 7.93–7.88 (m, 2H), 7.66 (d, *J* = 8.3 Hz, 1H), 7.44 (d, *J* = 7.0 Hz, 2H), 7.39 (t, *J* = 7.2 Hz, 2H), 7.33 (d, *J* = 7.4 Hz, 1H), 7.13 (d, *J* = 7.9 Hz, 2H), 6.96 (d, *J* = 8.4 Hz, 2H), 5.04 (s, 2H), 3.98 (s, 3H); ^13^C NMR (101 MHz, CDCl_3_) *δ* 165.64, 153.81, 139.14, 137.64, 137.20, 133.89, 133.11, 130.15, 128.53, 127.88, 127.62, 127.47, 126.54, 117.23, 116.04, 114.39, 112.24, 70.59, 52.70; HRMS-ESI (*m/z*) [M + H]^+^ calculated for C_23_H_20_N_3_O_3_ 386.14600, found 386.15051.

**Methyl (E)-4-cyano-3-((2-(2-(trifluoromethyl)phenyl)hydrazineylidene)methyl)benzoate (156)**

Yellow solid, 94.20 mg, 90% yield; ^1^H NMR (400 MHz, CDCl_3_) *δ* 8.62 (s, 1H), 8.46 (s, 1H), 8.10 (d, *J* = 1.6 Hz, 1H), 8.01 (dd, *J* = 7.6, 1.7 Hz, 1H), 7.90 (d, *J* = 8.3 Hz, 1H), 7.75 (d, *J* = 7.6 Hz, 1H), 7.54 (dd, *J* = 10.9, 7.8 Hz, 2H), 7.00 (t, *J* = 7.4 Hz, 1H), 3.99 (s, 3H); ^13^C NMR (101 MHz, CDCl_3_) *δ* 165.40, 141.09, 138.08, 134.19, 134.10, 133.51, 133.44, 128.83, 127.40, 126.20 (d, *J* = 5.2 Hz), 125.94, 120.43, 117.05, 115.45, 113.20, 112.89, 52.81; ^19^F NMR (376 MHz, CDCl_3_) *δ* -60.41; HRMS-ESI (*m/z*) [M + H]^+^ calculated for C_17_H_13_F_3_N_3_O_2_ 348.09152, found 348.09491.

**Methyl (E)-3-((2-(2-bromophenyl)hydrazineylidene)methyl)-4-cyanobenzoate (157)**

Yellow solid, 97.15 mg, 90% yield; ^1^H NMR (400 MHz, CDCl_3_) *δ* 8.64 (d, *J* = 1.6 Hz, 1H), 8.53 (s, 1H), 8.12 (d, *J* = 1.2 Hz, 1H), 7.99 (dd, *J* = 8.1, 1.6 Hz, 1H), 7.74 (d, *J* = 8.1 Hz, 1H), 7.70 (dd, *J* = 8.2, 1.6 Hz, 1H), 7.47 (dd, *J* = 8.0, 1.4 Hz, 1H), 7.33 (td, *J* = 7.8, 1.4 Hz, 1H), 6.82 (td, *J* = 7.7, 1.6 Hz, 1H), 4.00 (s, 3H); ^13^C NMR (101 MHz, CDCl_3_) *δ* 165.46, 140.33, 138.32, 134.04, 133.47, 133.36, 132.39, 128.77, 128.58, 127.13, 121.96, 117.08, 115.05, 113.07, 107.30, 52.80; HRMS-ESI (*m/z*) [M + H]^+^ calculated for C_16_H_13_BrN_3_O_2_ 358.01464, found 358.01860.

**Methyl (E)-4-cyano-3-((2-(2-fluorophenyl)hydrazineylidene)methyl)benzoate (158)**

Orange solid, 79.28 mg, 89% yield; ^1^H NMR (400 MHz, CDCl_3_) *δ* 8.63 (d, *J* = 1.7 Hz, 1H), 8.29 (s, 1H), 8.08 (d, *J* = 1.3 Hz, 1H), 7.98 (dd, *J* = 8.1, 1.7 Hz, 1H), 7.73 (d, *J* = 8.1 Hz, 1H), 7.71–7.66 (m, 1H), 7.16 (t, *J* = 7.7 Hz, 1H), 7.06 (ddd, *J* = 11.8, 8.2, 1.4 Hz, 1H), 6.88 (dddd, *J* = 8.2, 6.7, 5.0, 1.7 Hz, 1H), 4.00 (s, 3H); ^13^C NMR (101 MHz, CDCl_3_) *δ* 165.50, 151.01, 148.62, 138.42, 134.04, 133.26 (d, *J* = 16.7 Hz), 131.86 (d, *J* = 8.9 Hz), 128.48, 127.08, 125.06 (d, *J* = 3.6 Hz), 120.89 (d, *J* = 7.1 Hz), 117.05, 115.01 (d, *J* = 17.7 Hz), 115.00 (d, *J* = 2.1 Hz), 112.99, 52.78; ^19^F NMR (376 MHz, CDCl_3_) *δ* -136.50 (ddt, *J* = 12.0, 8.1, 4.4 Hz); HRMS-ESI (*m/z*) [M + H]^+^ calculated for C_16_H_13_FN_3_O_2_ 298.09471, found 298.09790.

**Methyl (E)-3-((2-(3-chlorophenyl)hydrazineylidene)methyl)-4-cyanobenzoate (159)**

Orange solid, 87.25 mg, 93% yield; ^1^H NMR (400 MHz, CDCl_3_) *δ* 8.64 (d, *J* = 1.7 Hz, 1H), 8.26 (s, 1H), 8.01 (s, 1H), 7.98 (dd, *J* = 8.1, 1.7 Hz, 1H), 7.72 (d, *J* = 8.1 Hz, 1H), 7.25–7.19 (m, 2H), 7.05–7.01 (m, 1H), 6.91 (ddd, *J* = 7.8, 2.1, 1.0 Hz, 1H), 4.00 (s, 3H); ^13^C NMR (101 MHz, CDCl_3_) *δ* 165.52, 144.59, 138.46, 135.28, 134.12, 133.20, 132.29, 130.44, 128.49, 126.83, 121.24, 117.00, 113.28, 112.99, 111.44, 52.82; HRMS-ESI (*m/z*) [M + H]^+^ calculated for C_16_H_13_ClN_3_O_2_ 314.06516, found 314.06923.

**Methyl (E)-4-cyano-3-((2-(2,3-dimethylphenyl)hydrazineylidene)methyl)benzoate (160)**

Red solid, 85.13 mg, 92% yield; ^1^H NMR (400 MHz, CDCl_3_) *δ* 8.65 (d, *J* = 1.7 Hz, 1H), 8.12 (s, 1H), 7.94 (s, 1H), 7.92 (dd, *J* = 8.1, 1.7 Hz, 1H), 7.68 (d, *J* = 8.1 Hz, 1H), 7.07 (d, *J* = 8.1 Hz, 1H), 6.99 (d, *J* = 2.4 Hz, 1H), 6.94 (dd, *J* = 8.0, 2.4 Hz, 1H), 3.99 (s, 3H), 2.27 (s, 3H), 2.22 (s, 3H); ^13^C NMR (101 MHz, CDCl_3_) *δ* 165.67, 141.39, 139.12, 137.71, 133.89, 133.08, 130.41, 130.29, 129.53, 127.69, 126.60, 117.20, 114.59, 112.41, 110.68, 52.73, 20.01, 18.98; HRMS-ESI (*m/z*) [M + H]^+^ calculated for C_18_H_18_N_3_O_2_ 308.13543, found 308.13992.

**Methyl (E)-3-((2-(2-chloro-4-fluorophenyl)hydrazineylidene)methyl)-4-cyanobenzoate (161)**

Yellow solid, 90.06 mg, 90% yield; ^1^H NMR (400 MHz, CDCl_3_) *δ* 8.59 (d, *J* = 1.6 Hz, 1H), 8.40 (s, 1H), 8.10 (s, 1H), 7.99 (dd, *J* = 8.0, 1.7 Hz, 1H), 7.74 (d, *J* = 8.1 Hz, 1H), 7.67 (dd, *J* = 9.1, 5.3 Hz, 1H), 7.11–7.00 (m, 2H), 3.99 (s, 3H); ^13^C NMR (101 MHz, CDCl_3_) *δ* 165.43, 156.84 (d, *J* = 242.7 Hz), 138.19, 136.20 (d, *J* = 2.9 Hz), 134.05, 133.57, 133.45, 128.61, 127.18, 117.41 (d, *J* = 10.3 Hz), 117.11, 116.31 (d, *J* = 26.0 Hz), 115.46 (d, *J* = 8.0 Hz), 115.25 (d, *J* = 22.2 Hz), 113.00, 52.82; ^19^F NMR (376 MHz, CDCl_3_) *δ* -121.75 (td, *J* = 8.0, 5.2 Hz); HRMS-ESI (*m/z*) [M + H]^+^ calculated for C_16_H_12_ClFN_3_O_2_ 332.05574, found 332.05927.

**Methyl (E)-3-((2-(2-chloro-4-methylphenyl)hydrazineylidene)methyl)-4-cyanobenzoate (162)**

Yellow solid, 90.25 mg, 92% yield; ^1^H NMR (400 MHz, CDCl_3_) *δ* 8.60 (d, *J* = 1.7 Hz, 1H), 8.46 (s, 1H), 8.06 (d, *J* = 1.3 Hz, 1H), 7.95 (dd, *J* = 8.1, 1.7 Hz, 1H), 7.70 (d, *J* = 8.1 Hz, 1H), 7.58 (d, *J* = 8.3 Hz, 1H), 7.11 (d, *J* = 1.9 Hz, 1H), 7.09–7.05 (m, 1H), 3.99 (s, 3H), 2.28 (s, 3H); ^13^C NMR (101 MHz, CDCl_3_) *δ* 165.49, 138.49, 137.07, 133.95, 133.27, 132.80, 131.21, 129.45, 128.79, 128.29, 126.97, 117.25, 117.09, 114.65, 112.85, 52.74, 20.37; HRMS-ESI (*m/z*) [M + H]^+^ calculated for C_17_H_15_ClN_3_O_2_ 328.08081, found 328.08445.

**Methyl (E)-4-cyano-3-((2-(2,4-dimethylphenyl)hydrazineylidene)methyl)benzoate (163)**

Brown-red oil, 87.35 mg, 95% yield; ^1^H NMR (400 MHz, CDCl_3_) *δ* 8.69 (d, *J* = 1.6 Hz, 1H), 8.07 (s, 1H), 7.99 (s, 1H), 7.94 (dd, *J* = 8.1, 1.6 Hz, 1H), 7.69 (d, *J* = 8.1 Hz, 1H), 7.51 (d, *J* = 8.2 Hz, 1H), 7.06 (dd, *J* = 8.2, 2.1 Hz, 1H), 6.94 (d, *J* = 2.0 Hz, 1H), 3.99 (d, *J* = 1.0 Hz, 3H), 2.29 (s, 3H), 2.26 (s, 3H); ^13^C NMR (101 MHz, CDCl_3_) *δ* 165.65, 139.09, 138.97, 134.00, 133.02, 131.30, 131.26, 130.57, 127.92, 126.58, 120.94, 117.18, 113.67, 112.62, 52.74, 20.58, 16.98; HRMS-ESI (*m/z*) [M + H]^+^ calculated for C_18_H_18_N_3_O_2_ 308.13543, found 308.13851.

**Methyl (E)-4-cyano-3-((2-(2,4-difluorophenyl)hydrazineylidene)methyl)benzoate (164)**

Yellow solid, 83.14 mg, 88% yield; ^1^H NMR (400 MHz, CDCl_3_) *δ* 8.60 (d, *J* = 1.6 Hz, 1H), 8.15 (s, 1H), 8.07 (d, *J* = 1.3 Hz, 1H), 7.99 (dd, *J* = 8.1, 1.7 Hz, 1H), 7.74 (d, *J* = 8.2 Hz, 1H), 7.65 (td, *J* = 9.1, 5.7 Hz, 1H), 6.95–6.84 (m, 2H), 4.00 (s, 3H); ^13^C NMR (101 MHz, CDCl_3_) *δ* 165.47, 138.26, 134.03, 133.45, 133.30, 128.54, 127.14, 117.10, 115.45 (d, *J* = 3.5 Hz), 115.36 (d, *J* = 3.4 Hz), 112.93, 111.84 (d, *J* = 3.6 Hz), 111.62 (d, *J* = 3.7 Hz), 103.71 (d, *J* = 5.1 Hz), 103.71 (d, *J* = 48.8 Hz), 52.82; ^19^F NMR (376 MHz, CDCl_3_) *δ* -120.64 – -120.77 , -132.56 (t, *J* = 10.6 Hz); HRMS-ESI (*m/z*) [M + H]^+^ calculated for C_16_H_12_F_2_N_3_O_2_ 316.08529, found 316.08945.

**Methyl (E)-4-cyano-3-((2-(2,5-dichlorophenyl)hydrazineylidene)methyl)benzoate (165)**

Yellow solid, 95.21 mg, 91% yield; ^1^H NMR (400 MHz, CDCl_3_) *δ* 8.65 (d, *J* = 1.6 Hz, 1H), 8.47 (s, 1H), 8.15 (s, 1H), 8.03 (dd, *J* = 8.1, 1.7 Hz, 1H), 7.75 (d, *J* = 8.1 Hz, 1H), 7.69 (d, *J* = 2.5 Hz, 1H), 7.23 (d, *J* = 8.5 Hz, 1H), 6.85 (dd, *J* = 8.5, 2.5 Hz, 1H), 4.01 (s, 3H); ^13^C NMR (101 MHz, CDCl_3_) *δ* 165.39, 140.23, 137.87, 134.80, 134.22, 134.13, 133.33, 130.09, 129.08, 127.12, 121.21, 116.91, 115.68, 114.61, 113.53, 52.87; HRMS-ESI (*m/z*) [M + H]^+^ calculated for C_16_H_12_Cl_2_N_3_O_2_ 348.02619, found 348.02931.

**Methyl (E)-3-((2-(2-chloro-5-(trifluoromethyl)phenyl)hydrazineylidene)methyl)-4-cyanobenzoate (166)**

Yellow solid, 10.3.25 mg, 90% yield; ^1^H NMR (400 MHz, CDCl_3_) *δ* 8.66 (d, *J* = 1.6 Hz, 1H), 8.57 (s, 1H), 8.20 (d, *J* = 1.2 Hz, 1H), 8.05 (dd, *J* = 8.1, 1.6 Hz, 1H), 7.96 (d, *J* = 2.2 Hz, 1H), 7.77 (d, *J* = 8.1 Hz, 1H), 7.44 (dd, *J* = 8.3, 1.0 Hz, 1H), 7.16–7.11 (m, 1H), 4.01 (s, 3H); ^13^C NMR (101 MHz, CDCl_3_) *δ* 165.33, 139.88, 137.66, 135.21, 134.23, 133.43, 130.72 (d, *J* = 32.9 Hz), 129.78, 129.24, 127.33, 122.38, 120.62, 117.67 (d, *J* = 3.9 Hz), 116.96, 113.57, 111.57 (d, *J* = 4.0 Hz), 52.89; ^19^F NMR (376 MHz, CDCl_3_) *δ* -62.74; HRMS-ESI (*m/z*) [M + H]^+^ calculated for C_17_H_12_ClF_3_N_3_O_2_ 382.05254, found 382.05627.

**Methyl (E)-4-cyano-3-((2-(3,5-dimethylphenyl)hydrazineylidene)methyl)benzoate (167)**

Red oil, 83.56 mg, 91% yield; ^1^H NMR (400 MHz, CDCl_3_) *δ* 8.67 (d, *J* = 1.7 Hz, 1H), 8.10 (s, 1H), 7.96 (s, 1H), 7.93 (dd, *J* = 8.1, 1.7 Hz, 1H), 7.69 (d, *J* = 8.1 Hz, 1H), 6.82 (s, 2H), 6.61 (s, 1H), 3.99 (s, 3H), 2.32 (s, 6H); ^13^C NMR (101 MHz, CDCl_3_) *δ* 165.67, 143.33, 139.27, 139.01, 133.97, 133.08, 130.73, 127.89, 126.67, 123.37, 117.15, 112.65, 111.11, 52.75, 21.47; HRMS-ESI (*m/z*) [M + H]^+^ calculated for C_18_H_18_N_3_O_2_ 308.13543, found 308.13841.

**Methyl (E)-3-((2-(4-bromo-2,6-dimethylphenyl)hydrazineylidene)methyl)-4-cyanobenzoate (168)**

Yellow solid, 104.55 mg, 90% yield; ^1^H NMR (400 MHz, CDCl_3_) *δ* 8.58 (d, *J* = 1.7 Hz, 1H), 7.94 (dd, *J* = 8.0, 1.7 Hz, 1H), 7.79 (s, 1H), 7.68 (d, *J* = 8.1 Hz, 1H), 7.60 (s, 1H), 7.26 (s, 2H), 3.96 (s, 3H), 2.34 (s, 6H); ^13^C NMR (101 MHz, CDCl_3_) *δ* 165.54, 139.19, 137.48, 134.00, 133.64, 132.94, 131.73, 130.50, 128.01, 126.23, 118.40, 116.93, 112.82, 52.69, 18.59; HRMS-ESI (*m/z*) [M + H]^+^ calculated for C_18_H_17_BrN_3_O_2_ 386.04594, found 386.04928.

**Methyl (E)-4-cyano-3-((2-mesitylhydrazineylidene)methyl)benzoate (169)**

Brown solid, 90.06 mg, 93% yield; ^1^H NMR (400 MHz, CDCl_3_) *δ* 8.57 (d, *J* = 1.7 Hz, 1H), 7.89 (dd, *J* = 8.1, 1.7 Hz, 1H), 7.63 (d, *J* = 8.0 Hz, 2H), 6.94 (s, 2H), 3.94 (s, 3H), 2.29 (s, 3H), 2.28 (s, 6H); ^13^C NMR (101 MHz, CDCl_3_) *δ* 165.64, 139.78, 136.19, 135.08, 133.80, 133.09, 132.86, 129.56, 128.88, 127.47, 126.17, 117.01, 112.51, 52.58, 20.84, 18.28; HRMS-ESI (*m/z*) [M + H]^+^ calculated for C_19_H_20_N_3_O_2_ 322.15108, found 322.15497.

**Methyl (E)-4-cyano-3-((2-(naphthalen-1-yl)hydrazineylidene)methyl)benzoate (170)**

Tan solid, 90.25 mg, 91% yield; ^1^H NMR (400 MHz, CDCl_3_) *δ* 8.78–8.73 (m, 2H), 8.24 (s, 1H), 7.97 (dt, *J* = 8.1, 1.4 Hz, 1H), 7.88 (td, *J* = 8.0, 2.4 Hz, 2H), 7.75–7.69 (m, 2H), 7.54–7.47 (m, 4H), 4.00 (s, 3H); ^13^C NMR (101 MHz, CDCl_3_) *δ* 165.56, 138.71, 138.07, 134.16, 134.06, 133.14, 133.07, 128.87, 128.36, 126.88, 126.55, 125.91, 125.54, 121.84, 121.48, 119.14, 117.15, 112.95, 109.42, 52.79; HRMS-ESI (*m/z*) [M + H]^+^ calculated for C_20_H_16_N_3_O_2_ 330.11978, found 330.12391.

**Methyl (E)-4-cyano-3-((2-(pyridin-2-yl)hydrazineylidene)methyl)benzoate (171)**

Yellow solid, 76.56 mg, 91% yield; ^1^H NMR (400 MHz, CDCl_3_) *δ* 8.72–8.67 (m, 1H), 8.61–8.55 (m, 1H), 8.47 (d, *J* = 1.7 Hz, 1H), 8.44–8.40 (m, 2H), 7.94–7.87 (m, 1H), 7.79–7.73 (m, 1H), 7.38 (ddd, *J* = 7.4, 4.9, 1.2 Hz, 1H), 4.02 (s, 3H); ^13^C NMR (101 MHz, CDCl_3_) *δ* 165.40, 158.68, 149.39, 149.29, 138.74, 137.97, 135.00, 132.15, 131.29, 129.47, 128.15, 127.79, 123.45, 121.16, 52.87; HRMS-ESI (*m/z*) [M + H]^+^ calculated for C_17_H_14_ClN_2_O_2_ 281.09938, found 281.10335.

**Methyl (E)-4-cyano-3-((2-(phenylsulfonyl)hydrazineylidene)methyl)benzoate (172)**

Light yellow solid, 95.66 mg, 93% yield; ^1^H NMR (400 MHz, CDCl_3_) *δ* 8.55 (d, *J* = 1.7 Hz, 1H), 8.17 (s, 1H), 8.04 (ddd, *J* = 10.1, 7.6, 1.7 Hz, 3H), 7.71 (d, *J* = 8.1 Hz, 1H), 7.62–7.50 (m, 3H), 3.98 (s, 3H); ^13^C NMR (101 MHz, CDCl_3_) *δ* 165.10, 141.29, 138.04, 136.56, 134.30, 133.53, 133.16, 130.49, 129.17, 127.93, 127.46, 116.17, 114.66, 52.93; HRMS-ESI (*m/z*) [M + H]^+^ calculated for C_16_H_14_N_3_O_4_S 344.06603, found 344.06941.

**Methyl (E)-4-cyano-3-((2-((4-methoxyphenyl)sulfonyl)hydrazineylidene)methyl)benzoate (173)**

Yellow solid, 102.33 mg, 92% yield; ^1^H NMR (400 MHz, CDCl_3_) *δ* 8.53 (d, *J* = 1.6 Hz, 1H), 8.15 (s, 1H), 8.04 (dd, *J* = 8.1, 1.7 Hz, 1H), 7.95 (d, *J* = 9.0 Hz, 2H), 7.70 (d, *J* = 8.0 Hz, 1H), 6.97 (d, *J* = 9.0 Hz, 3H), 3.97 (s, 3H), 3.83 (s, 3H). ^13^C NMR (101 MHz, CDCl_3_) *δ* 165.13, 163.57, 141.05, 136.67, 134.23, 133.16, 130.35, 130.20, 129.42, 127.48, 116.21, 114.59, 114.35, 55.60, 52.90; HRMS-ESI (*m/z*) [M + H]^+^ calculated for C_17_H_16_N_3_O_5_S 374.07660, found 374.07987.

**Methyl (E)-4-cyano-3-((2-((2,4,6-triisopropylphenyl)sulfonyl)hydrazineylidene)methyl)benzoate (174)**

Light yellow solid, 125.68 mg, 89% yield; ^1^H NMR (400 MHz, CDCl_3_) *δ* 9.31 (s, 1H), 8.61 (d, *J* = 1.7 Hz, 1H), 8.24 (s, 1H), 8.07 (dd, *J* = 8.1, 1.7 Hz, 1H), 7.72 (d, *J* = 8.1 Hz, 1H), 7.20 (s, 2H), 4.30 (p, *J* = 6.7 Hz, 2H), 3.94 (s, 3H), 2.90 (p, *J* = 6.9 Hz, 1H), 1.32 (d, *J* = 6.7 Hz, 12H), 1.25 (d, *J* = 6.9 Hz, 6H); ^13^C NMR (101 MHz, CDCl_3_) *δ* 165.07, 153.71, 151.48, 139.53, 137.05, 134.20, 132.83, 131.11, 130.32, 126.82, 123.96, 116.12, 114.73, 52.70, 34.15, 30.06, 24.84, 23.45; HRMS-ESI (*m/z*) [M + H]^+^ calculated for C_25_H_32_N_3_O_4_S 470.20688, found 470.21057.

**Methyl (E)-4-cyano-3-((2-(cyclopropanecarbonyl)hydrazineylidene)methyl)benzoate (175)**

Light yellow solid, 75.36 mg, 93% yield; ^1^H NMR (400 MHz, CDCl_3_) *δ* 10.19 (s, 1H), 8.57–8.53 (m, 1H), 8.16 (s, 1H), 8.08 (dd, *J* = 8.1, 1.7 Hz, 1H), 7.79 (d, *J* = 8.1 Hz, 1H), 3.98 (s, 3H), 2.81 (tt, *J* = 8.1, 4.6 Hz, 1H), 1.19 (dd, *J* = 4.5, 3.1 Hz, 2H), 1.05–1.00 (m, 2H); ^13^C NMR (101 MHz, CDCl_3_) *δ* 177.15, 165.19, 138.21, 137.07, 134.15, 133.85, 129.95, 128.27, 116.69, 114.19, 52.88, 10.45, 9.47; HRMS-ESI (*m/z*) [M + H]^+^ calculated for C_14_H_14_N_3_O_3_ 272.09905, found 272.10318.

**Methyl (E)-4-cyano-3-((2-(2-methoxybenzoyl)hydrazineylidene)methyl)benzoate (176)**

Light yellow solid, 91.05 mg, 90% yield; ^1^H NMR (400 MHz, CDCl_3_) *δ* 11.13 (s, 1H), 8.95 (d, *J* = 1.7 Hz, 1H), 8.64 (s, 1H), 8.33 (dd, *J* = 7.8, 1.9 Hz, 1H), 8.13 (dd, *J* = 8.1, 1.7 Hz, 1H), 7.77 (d, *J* = 8.1 Hz, 1H), 7.55 (ddd, *J* = 8.5, 7.3, 1.9 Hz, 1H), 7.19–7.13 (m, 1H), 7.06 (d, *J* = 8.3 Hz, 1H), 4.09 (s, 3H), 3.97 (s, 3H); ^13^C NMR (101 MHz, CDCl_3_) *δ* 165.17, 162.48, 157.31, 141.91, 137.46, 134.44, 134.06, 133.03, 132.92, 130.56, 127.75, 121.87, 119.76, 116.45, 114.98, 111.50, 56.33, 52.76; HRMS-ESI (*m/z*) [M + H]^+^ calculated for C_18_H_16_N_3_O_4_ 338.10961, found 338.11331.

**Benzyl (E)-2-(2-cyano-5-(methoxycarbonyl)benzylidene)hydrazine-1-carboxylate (177)**

Light yellow solid, 91.98 mg, 91% yield; ^1^H NMR (400 MHz, CDCl_3_) *δ* 9.13 (d, *J* = 14.1 Hz, 1H), 8.73 (s, 1H), 8.31 (s, 1H), 8.05 (dd, *J* = 8.2, 1.7 Hz, 1H), 7.68 (d, *J* = 8.0 Hz, 1H), 7.40 (d, *J* = 8.1 Hz, 2H), 7.34 (dtd, *J* = 6.8, 4.9, 2.9 Hz, 3H), 5.29 (s, 2H), 3.94 (s, 3H); ^13^C NMR (101 MHz, CDCl_3_) *δ* 165.16, 137.23, 135.47, 134.25, 132.83, 130.19, 128.55, 128.45, 128.34, 127.22, 116.25, 114.70, 67.84, 52.78; HRMS-ESI (*m/z*) [M + H]^+^ calculated for C_18_H_16_N_3_O_4_ 338.10961, found 338.11260.

**4-methoxybenzyl (E)-2-(2-cyano-5-(methoxycarbonyl)benzylidene)hydrazine-1-carboxylate (178)**

Light yellow solid, 102.32 mg, 93% yield; ^1^H NMR (400 MHz, CDCl_3_) *δ* 8.79–8.69 (m, 2H), 8.29 (s, 1H), 8.07 (dd, *J* = 8.1, 1.7 Hz, 1H), 7.71 (d, *J* = 8.1 Hz, 1H), 7.36 (d, *J* = 8.6 Hz, 2H), 6.89 (d, *J* = 8.7 Hz, 2H), 5.23 (s, 2H), 3.96 (s, 3H), 3.81 (s, 3H); ^13^C NMR (101 MHz, CDCl_3_) *δ* 165.16, 159.86, 137.18, 134.33, 132.86, 130.40, 130.26, 127.56, 127.36, 116.26, 114.78, 113.99, 67.81, 55.27, 52.77; HRMS-ESI (*m/z*) [M + Na]^+^ calculated for C_19_H_17_NaN_3_O_5_ 390.11682, found 390.10629.

**Methyl (E)-4-cyano-3-((2-methyl-2-phenylhydrazineylidene)methyl)benzoate (179)**

Orange solid, 80.36 mg, 91% yield; ^1^H NMR (400 MHz, CDCl_3_) *δ* 8.66 (d, *J* = 1.7 Hz, 1H), 7.91 (dd, *J* = 8.1, 1.7 Hz, 1H), 7.72–7.67 (m, 2H), 7.47–7.42 (m, 2H), 7.38 (dd, *J* = 8.8, 7.2 Hz, 2H), 7.04 (tt, *J* = 7.2, 1.3 Hz, 1H), 3.98 (s, 3H), 3.52 (d, *J* = 0.9 Hz, 3H); ^13^C NMR (101 MHz, CDCl_3_) *δ* 165.76, 147.06, 140.23, 133.89, 133.12, 129.16, 127.30, 126.56, 126.13, 122.16, 117.48, 116.19, 112.69, 52.68, 33.88; HRMS-ESI (*m/z*) [M + H]^+^ calculated for C_17_H_16_N_3_O_2_ 294.11978, found 294.12301.

**Methyl (E)-3-(((1H-indol-1-yl)imino)methyl)-4-cyanobenzoate (180)**

Orange solid, 85.23 mg, 94% yield; ^1^H NMR (400 MHz, CDCl_3_) *δ* 8.83 (d, *J* = 1.6 Hz, 1H), 8.63 (s, 1H), 8.10 (dd, *J* = 7.7, 1.6 Hz, 1H), 7.91 (d, *J* = 7.7 Hz, 1H), 7.80 (d, *J* = 7.7 Hz, 1H), 7.74 (d, *J* = 3.6 Hz, 1H), 7.59 (d, *J* = 7.5 Hz, 1H), 7.36 (t, *J* = 7.3 Hz, 1H), 7.22 (t, *J* = 7.2 Hz, 1H), 6.76 (d, *J* = 3.4 Hz, 1H), 4.02 (s, 3H); ^13^C NMR (101 MHz, CDCl_3_) *δ* 165.23, 137.41, 137.34, 136.79, 134.22, 133.54, 129.98, 127.77, 127.08, 124.11, 122.03, 121.08, 116.73, 116.45, 114.51, 111.04, 106.67, 52.89; HRMS-ESI (*m/z*) [M + H]^+^ calculated for C_18_H_14_N_3_O_2_ 304.10413, found 304.10757.

**Methyl (E)-4-cyano-3-(((2-methylindolin-1-yl)imino)methyl)benzoate (181)**

Orange solid, 90.35 mg, 94% yield; ^1^H NMR (400 MHz, CDCl_3_) *δ* 8.66 (d, *J* = 1.7 Hz, 1H), 7.91 (dd, *J* = 8.1, 1.7 Hz, 1H), 7.76 (d, *J* = 1.1 Hz, 1H), 7.71 (d, *J* = 8.0 Hz, 1H), 7.37 (d, *J* = 7.9 Hz, 1H), 7.29 (d, *J* = 9.3 Hz, 1H), 7.20 (d, *J* = 7.4 Hz, 1H), 6.95 (td, *J* = 7.3, 1.2 Hz, 1H), 4.68 (dddd, *J* = 11.9, 6.7, 3.3, 1.8 Hz, 1H), 4.03 (s, 3H), 3.58 (dd, *J* = 16.0, 9.4 Hz, 1H), 2.86 (dd, *J* = 16.0, 3.0 Hz, 1H), 1.45 (d, *J* = 6.2 Hz, 3H); ^13^C NMR (101 MHz, CDCl_3_) *δ* 165.78, 145.93, 140.14, 133.79, 133.13, 128.10, 126.91, 126.69, 126.21, 125.67, 125.20, 121.75, 117.53, 111.99, 109.99, 55.07, 52.63, 36.36, 17.37; HRMS-ESI (*m/z*) [M + H]^+^ calculated for C_19_H_18_N_3_O_2_ 320.13543, found 320.13876.

**Methyl (E)-4-cyano-3-(((2-cyano-1H-pyrrol-1-yl)imino)methyl)benzoate (182)**

Yellow solid, 76.28 mg, 91% yield; ^1^H NMR (400 MHz, CDCl_3_) *δ* 8.89 (d, *J* = 1.7 Hz, 1H), 8.84 (s, 1H), 8.24 (dd, *J* = 8.1, 1.7 Hz, 1H), 7.85 (d, *J* = 8.1 Hz, 1H), 7.50 (dd, *J* = 3.2, 1.5 Hz, 1H), 6.93 (dd, *J* = 4.2, 1.5 Hz, 1H), 6.42 (dd, *J* = 4.1, 3.2 Hz, 1H), 4.00 (s, 3H); ^13^C NMR (101 MHz, CDCl_3_) *δ* 164.80, 145.09, 135.38, 134.78, 133.34, 132.04, 127.99, 120.24, 116.26, 115.90, 115.55, 112.01, 111.12, 106.18, 53.03; HRMS-ESI (*m/z*) [M + H]^+^ calculated for C_15_H_11_N_4_O_2_ 279.08373, found 279.08859.

**Methyl (E)-4-cyano-3-(((3,4-dihydroquinolin-1(2H)-yl)imino)methyl)benzoate (183)**

Brown solid, 90.11 mg, 94% yield; ^1^H NMR (400 MHz, CDCl_3_) *δ* 8.64 (d, *J* = 1.7 Hz, 1H), 7.84 (ddd, *J* = 16.0, 8.2, 1.4 Hz, 2H), 7.69 (s, 1H), 7.65 (d, *J* = 8.2 Hz, 1H), 7.24 (td, *J* = 7.7, 1.6 Hz, 1H), 7.06–7.01 (m, 1H), 6.88 (td, *J* = 7.3, 1.2 Hz, 1H), 3.96 (s, 3H), 3.69 (t, *J* = 6.4 Hz, 2H), 2.75 (d, *J* = 6.2 Hz, 2H), 2.18 (p, *J* = 6.1 Hz, 2H); ^13^C NMR (101 MHz, CDCl_3_) *δ* 165.74, 142.00, 140.41, 133.81, 133.08, 128.31, 127.60, 127.09, 126.45, 124.78, 124.68, 121.10, 117.51, 115.20, 112.49, 52.63, 45.60, 26.82, 21.73; HRMS-ESI (*m/z*) [M + H]^+^ calculated for C_18_H_14_N_3_O_2_ 320.10159, found 320.10272.

**Methyl (E)-4-cyano-3-((phenoxyimino)methyl)benzoate (184)**

Light yellow solid, 78.15 mg, 93% yield; ^1^H NMR (400 MHz, CDCl_3_) *δ* 8.76 (s, 1H), 8.65 (d, *J* = 1.7 Hz, 1H), 8.15 (dd, *J* = 8.1, 1.7 Hz, 1H), 7.82 (d, *J* = 8.1 Hz, 1H), 7.37 (dd, *J* = 8.8, 7.1 Hz, 2H), 7.34–7.28 (m, 2H), 7.12–7.07 (m, 1H), 3.99 (s, 3H); ^13^C NMR (101 MHz, CDCl_3_) *δ* 164.95, 158.92, 147.11, 134.70, 134.28, 133.63, 130.86, 129.44, 128.26, 123.13, 116.25, 115.15, 114.62, 52.89; HRMS-ESI (*m/z*) [M + H]^+^ calculated for C_16_H_13_N_2_O_3_ 281.08815, found 281.09292.

**Methyl (E)-4-cyano-3-((fluoranthen-3-ylimino)methyl)benzoate (185)**

Yellow solid, 110.02 mg, 94% yield; ^1^H NMR (400 MHz, CDCl_3_) *δ* 8.99 (d, *J* = 1.5 Hz, 2H), 8.29 (d, *J* = 8.3 Hz, 1H), 8.21 (dd, *J* = 8.1, 1.7 Hz, 1H), 7.97 (d, *J* = 6.9 Hz, 1H), 7.93 (d, *J* = 7.4 Hz, 1H), 7.92–7.88 (m, 2H), 7.86 (d, *J* = 8.0 Hz, 1H), 7.69 (dd, *J* = 8.3, 6.9 Hz, 1H), 7.41–7.36 (m, 2H), 7.33 (d, *J* = 7.3 Hz, 1H), 4.03 (s, 3H); ^13^C NMR (101 MHz, CDCl_3_) *δ* 165.16, 154.80, 147.63, 139.71, 138.90, 138.60, 136.61, 136.41, 134.30, 133.69, 133.16, 131.59, 129.46, 128.29, 127.67, 127.54, 126.11, 123.69, 121.61, 121.51, 120.76, 120.59, 116.54, 116.42, 115.13, 52.92; HRMS-ESI (*m/z*) [M + H]^+^ calculated for C_26_H_17_N_2_O_2_ 389.12453, found 389.12810.

**Methyl (E)-4-cyano-3-((pyren-1-ylimino)methyl)benzoate (186)**

Orange solid, 110.89 mg, 95% yield; ^1^H NMR (400 MHz, CDCl_3_) *δ* 9.08 (s, 1H), 9.01 (d, *J* = 1.7 Hz, 1H), 8.81 (d, *J* = 9.1 Hz, 1H), 8.26–8.12 (m, 5H), 8.08–7.95 (m, 3H), 7.88 (dd, *J* = 15.0, 7.6 Hz, 1H), 7.81 (d, *J* = 8.0 Hz, 1H), 4.05 (s, 3H); ^13^C NMR (101 MHz, CDCl_3_) *δ* 165.20, 154.25, 143.32, 138.90, 134.20, 133.61, 131.41, 131.35, 131.26, 130.92, 129.31, 127.76, 127.55, 127.21, 126.56, 126.27, 125.56, 125.46, 125.38, 125.24, 124.64, 123.22, 116.69, 116.23, 114.83, 52.90; HRMS-ESI (*m/z*) [M + H]^+^ calculated for C_26_H_17_N_2_O_2_ 389.12453, found 389.12839.

**Methyl (E)-4-cyano-3-((phenanthren-1-ylimino)methyl)benzoate (187)**

Yellow solid, 100.28 mg, 92% yield; ^1^H NMR (400 MHz, CDCl_3_) *δ* 9.09–9.05 (m, 2H), 8.74–8.65 (m, 2H), 8.47 (dd, *J* = 8.0, 1.5 Hz, 1H), 8.25 (dd, *J* = 8.1, 1.7 Hz, 1H), 7.93 (dd, *J* = 7.3, 1.9 Hz, 1H), 7.89 (d, *J* = 8.1 Hz, 1H), 7.75–7.60 (m, 4H), 7.38 (s, 1H), 4.03 (s, 3H); ^13^C NMR (101 MHz, CDCl_3_) *δ* 165.18, 154.65, 146.79, 138.68, 134.44, 133.67, 132.02, 131.68, 130.81, 129.94, 129.49, 128.94, 128.69, 127.39, 127.05, 126.82, 126.47, 124.65, 122.67, 116.60, 116.48, 112.16, 52.92; HRMS-ESI (*m/z*) [M + H]^+^ calculated for C_24_H_17_N_2_O_2_ 365.12453, found 365.12836.

**Methyl (E)-4-cyano-3-(((4-((2-(methylcarbamoyl)pyridin-4-yl)oxy)phenyl)imino)methyl)benzoate (188)**

Orange solid, 115.29 mg, 93% yield; ^1^H NMR (400 MHz, CDCl_3_) *δ* 8.92 (d, *J* = 1.8 Hz, 1H), 8.87 (s, 1H), 8.38 (d, *J* = 5.6 Hz, 1H), 8.20 (dd, *J* = 8.1, 1.7 Hz, 1H), 8.02 (s, 1H), 7.83 (d, *J* = 8.1 Hz, 1H), 7.74 (d, *J* = 2.6 Hz, 1H), 7.39 (d, *J* = 8.7 Hz, 2H), 7.15 (d, *J* = 8.8 Hz, 2H), 6.98 (dd, *J* = 5.5, 2.6 Hz, 1H), 3.99 (s, 3H), 3.00 (d, *J* = 5.1 Hz, 3H); ^13^C NMR (101 MHz, CDCl_3_) *δ* 165.95, 165.06, 164.38, 154.45, 153.06, 152.30, 149.73, 147.62, 138.41, 134.29, 133.37, 131.58, 128.82, 123.14, 121.60, 116.66, 116.17, 114.16, 110.38, 52.82, 26.08; HRMS-ESI (*m/z*) [M + H]^+^ calculated for C_23_H_19_N_4_O_4_ 415.13616, found 415.13971.

**Methyl (E)-4-cyano-3-(((1,5-dimethyl-3-oxo-2-phenyl-2,3-dihydro-1H-pyrazol-4-yl)imino)methyl)benzoate (189)**

Yellow solid, 103.59 mg, 92% yield; ^1^H NMR (400 MHz, CDCl_3_) *δ* 9.91 (s, 1H), 8.52 (d, *J* = 1.7 Hz, 1H), 8.05 (dd, *J* = 8.0, 1.7 Hz, 1H), 7.79 (d, *J* = 8.1 Hz, 1H), 7.52–7.46 (m, 2H), 7.41–7.35 (m, 3H), 3.96 (s, 3H), 3.24 (s, 3H), 2.58 (s, 3H); ^13^C NMR (101 MHz, CDCl_3_) *δ* 165.43, 160.07, 152.94, 150.80, 140.62, 134.58, 134.28, 133.71, 130.12, 129.73, 129.31, 127.46, 125.00, 117.75, 117.36, 113.74, 52.69, 35.24, 10.26; HRMS-ESI (*m/z*) [M + H]^+^ calculated for C_21_H_19_N_4_O_3_ 375.14125, found 375.14511.

**Methyl (E)-3-(((4-chloro-3-((3-chloro-5-(trifluoromethyl)pyridin-2-yl)oxy)phenyl)imino)methyl)-4-cyanobenzoate (190)**

Yellow solid, 136.66 mg, 92% yield; ^1^H NMR (400 MHz, CDCl_3_) *δ* 8.93 (d, *J* = 1.7 Hz, 1H), 8.89 (s, 1H), 8.27 (dd, *J* = 2.2, 1.1 Hz, 1H), 8.23 (dd, *J* = 8.1, 1.7 Hz, 1H), 8.03 (d, *J* = 2.2 Hz, 1H), 7.85 (d, *J* = 8.1 Hz, 1H), 7.56 (dt, *J* = 8.8, 1.2 Hz, 1H), 7.27 (s, 1H), 7.25 (d, *J* = 2.4 Hz, 1H), 4.00 (s, 3H); ^13^C NMR (101 MHz, CDCl_3_) *δ* 165.01, 160.13, 155.43, 149.63 (d, *J* = 55.0 Hz), 142.54 (d, *J* = 4.5 Hz), 138.10, 136.61 (d, *J* = 3.5 Hz), 134.40, 133.48, 131.94, 131.06, 129.00, 126.01, 124.09, 123.16, 122.83, 121.38, 120.12, 119.00, 116.81, 116.13, 52.91; ^19^F NMR (376 MHz, CDCl_3_) *δ* -61.61; HRMS-ESI (*m/z*) [M + H]^+^ calculated for C_22_H_13_Cl_2_F_3_N_3_O_3_ 494.02414, found 494.02758.

**Methyl (E)-4-cyano-3-(((4-(3-ethyl-2,6-dioxopiperidin-3-yl)phenyl)imino)methyl)benzoate (191)**

Yellow solid, 112.18 mg, 93% yield; ^1^H NMR (400 MHz, CDCl_3_) *δ* 8.92 (d, *J* = 1.7 Hz, 1H), 8.85 (s, 1H), 8.27 (s, 1H), 8.20 (dd, *J* = 8.0, 1.7 Hz, 1H), 7.84 (d, *J* = 8.1 Hz, 1H), 7.40–7.28 (m, 4H), 3.99 (s, 3H), 2.68–2.58 (m, 1H), 2.50–2.36 (m, 2H), 2.28 (dd, *J* = 14.1, 4.8 Hz, 1H), 2.08 (dd, *J* = 14.3, 7.3 Hz, 1H), 1.95 (dd, *J* = 14.2, 7.3 Hz, 1H), 0.89 (t, *J* = 7.4 Hz, 3H); ^13^C NMR (101 MHz, CDCl_3_) *δ* 175.00, 172.24, 165.08, 154.94, 149.61, 138.42, 137.96, 134.33, 133.38, 131.65, 128.85, 127.25, 121.72, 116.73, 116.18, 52.85, 50.86, 32.87, 29.23, 27.06, 8.99; HRMS-ESI (*m/z*) [M + H]^+^ calculated for C_23_H_22_N_3_O_4_ 404.15656, found 404.15997.

**Methyl (E)-4-cyano-3-(((3-fluoro-4-morpholinophenyl)imino)methyl)benzoate (192)**

Brown solid, 100.42 mg, 91% yield; ^1^H NMR (400 MHz, CDCl_3_) *δ* 8.92 (d, *J* = 1.7 Hz, 1H), 8.86 (s, 1H), 8.19 (dd, *J* = 8.1, 1.7 Hz, 1H), 7.83 (d, *J* = 8.1 Hz, 1H), 7.16 (d, *J* = 1.4 Hz, 1H), 7.16–7.11 (m, 1H), 6.98 (t, *J* = 9.0 Hz, 1H), 3.99 (s, 3H), 3.92–3.87 (m, 4H), 3.17–3.13 (m, 4H); ^13^C NMR (101 MHz, CDCl_3_) *δ* 165.17, 155.60 (d, *J* = 248.0 Hz), 152.80, 144.47 (d, *J* = 8.3 Hz), 139.58, 138.66, 134.30, 133.35, 131.35, 128.66, 118.69 (d, *J* = 4.1 Hz), 118.13 (d, *J* = 2.9 Hz), 116.50, 116.31, 109.71 (d, *J* = 22.0 Hz), 66.89, 52.86, 50.76 (d, *J* = 3.5 Hz); ^19^F NMR (376 MHz, CDCl_3_) *δ* -121.00 (dd, *J* = 13.3, 9.2 Hz); HRMS-ESI (*m/z*) [M + H]^+^ calculated for C_20_H_19_FN_3_O_3_ 368.13657, found 368.14069.

**Methyl (E)-4-cyano-3-(((4-methyl-3-((4-(pyridin-3-yl)thiazol-2-yl)amino)phenyl)imino)methyl)benzoate (193)**

Yellow solid, 127.11 mg, 93% yield; ^1^H NMR (400 MHz, CDCl_3_) *δ* 9.10–9.07 (m, 1H), 8.96 (d, *J* = 1.7 Hz, 1H), 8.93 (s, 1H), 8.56–8.52 (m, 1H), 8.21 (dd, *J* = 8.1, 1.7 Hz, 1H), 8.19–8.14 (m, 1H), 7.85 (dd, *J* = 8.1, 0.6 Hz, 1H), 7.77 (d, *J* = 2.1 Hz, 1H), 7.38–7.29 (m, 2H), 7.07 (dd, *J* = 8.0, 2.1 Hz, 1H), 6.95 (s, 1H), 4.00 (s, 3H), 2.40 (s, 3H); ^13^C NMR (101 MHz, CDCl_3_) *δ* 165.97, 165.15, 154.25, 149.48, 148.53, 148.35, 147.34, 139.30, 138.59, 134.30, 133.44, 133.36, 131.80, 131.51, 130.40, 128.87, 127.88, 123.56, 116.92, 116.67, 116.26, 112.94, 103.57, 52.85, 17.60; HRMS-ESI (*m/z*) [M + H]^+^ calculated for C_25_H_20_N_5_O_2_S 454.12930, found 454.13330.

**Methyl (E)-4-cyano-3-((2-(perfluorophenyl)hydrazineylidene)methyl)benzoate (194)**

Yellow solid, 100.65 mg, 91% yield; ^1^H NMR (400 MHz, CDCl_3_) *δ* 8.67 (d, *J* = 1.8 Hz, 1H), 8.20 (s, 1H), 8.04 (dd, *J* = 8.1, 1.7 Hz, 1H), 7.91 (s, 1H), 7.74 (d, *J* = 8.1 Hz, 1H), 3.98 (s, 3H); ^13^C NMR (101 MHz, CDCl_3_) *δ* 164.30, 138.60, 136.54, 136.14, 135.99, 135.59, 133.32, 131.92, 128.49, 125.73, 118.35, 115.48, 112.88, 51.87; ^19^F NMR (376 MHz, CDCl_3_) *δ* -155.20 (dd, *J* = 22.5, 5.3 Hz), -162.64 (td, *J* = 21.8, 5.4 Hz), -164.84–165.02 . HRMS-ESI (*m/z*) [M + H]^+^ calculated for C_16_H_9_F_5_N_3_O_2_ 370.05702, found 370.06140.

**Methyl (E)-4-cyano-3-(((4-(2-oxopyrrolidin-1-yl)phenyl)imino)methyl)benzoate (195)**

Red solid, 95.10 mg, 91% yield; ^1^H NMR (400 MHz, CDCl_3_) *δ* 8.94 (d, *J* = 1.7 Hz, 1H), 8.89 (s, 1H), 8.21–8.18 (m, 1H), 7.83 (dd, *J* = 8.1, 0.6 Hz, 1H), 7.74–7.70 (m, 2H), 7.42–7.36 (m, 2H), 4.00 (s, 3H), 3.91 (t, *J* = 7.0 Hz, 2H), 2.65 (dd, *J* = 8.5, 7.6 Hz, 2H), 2.20 (p, *J* = 7.5 Hz, 2H); ^13^C NMR (101 MHz, CDCl_3_) *δ* 174.37, 165.19, 153.45, 146.14, 138.99, 138.78, 134.31, 133.35, 131.38, 128.73, 121.92, 120.46, 116.59, 116.32, 52.84, 48.77, 32.74, 17.94; HRMS-ESI (*m/z*) [M + H]^+^ calculated for C_20_H_18_N_3_O_3_ 348.13035, found 348.13351.

**Methyl (E)-3-((benzo[d][**1,3**]dioxol-5-ylimino)methyl)-4-cyanobenzoate (196)**

Yellow solid, 86.23 mg, 93% yield; ^1^H NMR (400 MHz, CDCl_3_) *δ* 8.89 (d, *J* = 1.9 Hz, 1H), 8.83 (s, 1H), 8.16 (dd, *J* = 8.1, 1.7 Hz, 1H), 7.80 (d, *J* = 8.1 Hz, 1H), 6.92 (d, *J* = 7.5 Hz, 2H), 6.90–6.82 (m, 1H), 3.98 (s, 3H); ^13^C NMR (101 MHz, CDCl_3_) *δ* 165.19, 151.89, 148.51, 147.53, 144.39, 138.83, 134.22, 133.28, 131.10, 128.57, 116.48, 116.37, 116.34, 108.42, 101.65, 52.79; HRMS-ESI (*m/z*) [M + H]^+^ calculated for C_17_H_13_N_2_O_4_ 309.08306, found 309.08707.

**Methyl (E)-4-cyano-3-(((2,3-dihydrobenzo[b][1,4]dioxin-6-yl)imino)methyl)benzoate (197)**

Orange solid, 90.25 mg, 93% yield; ^1^H NMR (400 MHz, CDCl_3_) *δ* 8.89 (d, *J* = 1.7 Hz, 1H), 8.83 (s, 1H), 8.15 (dd, *J* = 8.1, 1.7 Hz, 1H), 7.80 (d, *J* = 8.0 Hz, 1H), 6.96–6.89 (m, 3H), 4.28 (s, 4H), 3.98 (s, 3H); ^13^C NMR (101 MHz, CDCl_3_) *δ* 165.18, 152.19, 143.83, 143.67, 143.58, 138.87, 134.18, 133.23, 131.05, 128.51, 117.64, 116.39, 116.31, 115.29, 110.20, 64.36, 64.26, 52.75; HRMS-ESI (*m/z*) [M + H]^+^ calculated for C_18_H_15_N_2_O_4_ 323.09871, found 323.10175.

**Methyl (E)-3-(((4-(benzyloxy)phenyl)imino)methyl)-4-cyanobenzoate (198)**

Red solid, 103.58 mg, 93% yield; ^1^H NMR (400 MHz, CDCl_3_) *δ* 8.94 (d, *J* = 1.8 Hz, 1H), 8.89 (s, 1H), 8.17 (dd, *J* = 8.1, 1.7 Hz, 1H), 7.81 (d, *J* = 8.0 Hz, 1H), 7.48–7.35 (m, 7H), 7.04 (d, *J* = 8.9 Hz, 2H), 5.11 (s, 2H), 3.99 (s, 3H); ^13^C NMR (101 MHz, CDCl_3_) *δ* 165.26, 158.70, 151.79, 143.20, 139.06, 136.63, 134.25, 133.28, 131.05, 128.62, 128.54, 128.07, 127.48, 122.93, 116.42, 116.37, 115.49, 70.25, 52.81; HRMS-ESI (*m/z*) [M + H]^+^ calculated for C_23_H_19_N_2_O_3_ 371.13510, found 371.13898.

**Methyl (E)-4-cyano-3-(((3-iodo-1H-indazol-5-yl)imino)methyl)benzoate (199)**

Yellow solid, 120.05 mg, 93% yield; ^1^H NMR (400 MHz, CDCl_3_) *δ* 8.98 (d, *J* = 4.0 Hz, 2H), 8.22 (dd, *J* = 8.1, 1.7 Hz, 1H), 7.86 (d, *J* = 8.1 Hz, 1H), 7.56 (d, *J* = 1.3 Hz, 2H), 7.47 (t, *J* = 1.3 Hz, 1H), 4.02 (s, 3H); ^13^C NMR (101 MHz, CDCl_3_) *δ* 165.21, 153.67, 144.96, 139.90, 138.73, 134.42, 133.47, 131.48, 128.91, 128.32, 123.09, 116.57, 116.40, 113.47, 110.86, 94.68, 52.88; HRMS-ESI (*m/z*) [M + H]^+^ calculated for C_17_H_12_IN_4_O_2_ 430.99602, found 430.99937.

**Methyl (E)-4-cyano-3-(((4'-cyano-[1,1'-biphenyl]-4-yl)imino)methyl)benzoate (200)**

Orange solid, 100.12 mg, 91% yield; ^1^H NMR (400 MHz, CDCl_3_) *δ* 8.97 (d, *J* = 1.7 Hz, 1H), 8.92 (s, 1H), 8.23 (dd, *J* = 8.0, 1.7 Hz, 1H), 7.86 (d, *J* = 8.1 Hz, 1H), 7.73 (d, *J* = 3.0 Hz, 3H), 7.67 (d, *J* = 8.5 Hz, 2H), 7.63 (d, *J* = 11.0 Hz, 1H), 7.43 (d, *J* = 8.3 Hz, 2H), 4.01 (s, 3H); ^13^C NMR (101 MHz, CDCl_3_) *δ* 165.08, 154.93, 150.58, 144.67, 138.41, 138.09, 134.37, 133.42, 132.64, 132.47, 131.73, 128.89, 128.19, 128.16, 127.50, 126.53, 121.98, 118.85, 116.76, 116.21, 115.33, 110.98, 52.89; HRMS-ESI (*m/z*) [M + H]^+^ calculated for C_23_H_16_N_3_O_2_ 366.11978, found 366.12405.

## Data Availability

The data are provided in electronic supplementary material [78].
